# A New Water-Soluble Copper(II) Coordination Polymer for Electrocatalytic Oxygen Evolution Reaction

**DOI:** 10.1021/acsorginorgau.5c00077

**Published:** 2026-01-27

**Authors:** Renata P. P. Macedo, Joice L. Carvalho, P.R.A de Oliveira, Lorenzo C. Visentin, Nakédia M. F. Carvalho

**Affiliations:** a Instituto de Química, 28130 Universidade do Estado do Rio de Janeiro, Rua São Francisco Xavier, 524, Maracanã, Rio de Janeiro, RJ 20550-900, Brazil; b Instituto de Física, 28125 Universidade Federal do Rio de Janeiro, Av. Athos da Silveira Ramos, 149, Cidade Universitária, Rio de Janeiro, RJ 21941-909, Brazil; c Nanobusiness Informação e Inovação Ltda, Avenida das Américas, 2480, Barra da Tijuca, Rio de Janeiro, RJ 22640-101, Brazil

**Keywords:** copper(II) coordination polymer, carboxylate-appended ligand, single-crystal X-ray diffraction, water oxidation reaction, electrocatalysis

## Abstract

The development of efficient and low-cost catalysts to promote the oxygen evolution reaction (OER) is a challenge for the generation of green hydrogen through water splitting electrolysis. This work describes the synthesis of a new water-soluble one-dimensional copper­(II) coordination polymer for application as an earth-abundant OER molecular electrocatalyst. The ligand was designed to have nitrogenated and carboxylate groups, inspired by the amino acid residues of the Mn_4_CaO_5_ cluster of photosystem II (PSII). The multiple coordination sites with different Lewis basicity were envisioned to infer structural stability and modulated electronic structure of the Cu­(II) center allowing redox processes to occur during the OER. Furthermore, a *N*-propanoate linker allows structural flexibility for the formation of carboxylate bridges and the coordination polymer. The single-crystal diffraction structure reveals an unusual monatomic bridge involving the chelation of the carboxylate group to two adjacent Cu­(II) ions. The electrocatalysis of the OER was conducted in a phosphate buffer at pH 12.5, and an onset overpotential of 394 mV was achieved at a current density of 0.1 mA cm^–2^, comparable to those reported for Cu^II^ molecular catalysts. Kinetic data show first-order electron transfer with a turnover frequency of 2.1 s^–1^. Stability tests by chronopotentiometry and cyclic voltammetry showed the formation of a CuOx-OH film at the working electrode surface, indicating the contribution of heterogeneous electrocatalysis for the OER.

## Graphical abstract

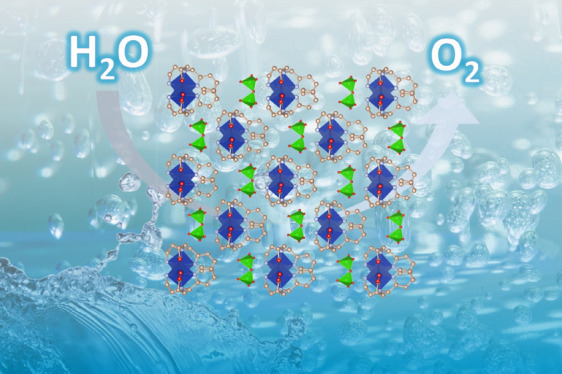

## 1 Introduction

The implementation of water splitting into hydrogen and oxygen to produce renewable sources of energy and fuels is one of the biggest challenges in modern science. Green hydrogen is considered the most promising fuel for transport and chemical industry feedstock, and its production by water electrolysis is still far from practical applications in the artificial photosynthesis scenario. The oxygen evolution reaction (OER) is the most thermodynamically and kinetically demanding half-reaction and requires high overpotential to transfer four protons and four electrons and form the O–O bond.^1^

The Mn_4_CaO_5_ cluster of the oxygen evolving complex (OEC) present in the natural photosystem PSII oxidizes water with low overpotential at the expense of solar energy and has been the inspiration for tailoring water oxidation catalysts (WOCs). Heterogeneous or homogeneous WOCs based on noble metals such as iridium and ruthenium have shown the highest activities; however, intensive efforts have been dedicated to the development of efficient, stable, and low-cost catalysts based on earth-abundant first-row transition metals such as manganese, iron, cobalt, nickel, and copper.^1^ Furthermore, the electronic structure, geometry, and coordination sphere of the active site in homogeneous electrocatalysts can be finely tuned to improve the performance toward the OER while allowing a more straightforward correlation between structure and activity and investigation of the catalytic mechanism. Although the limited long-term stability of the homogeneous catalysts is a huge drawback, their intrinsic activity usually surpasses that of heterogeneous catalysts and has motivated the improvement of these classes of compounds through rational ligand design. On the other hand, ligand oxidation and release of metallic species can take place during the water electrolysis, resulting in the formation of metal oxides that can present distinguished properties such as surface area, morphology, porosity, and consequently high activity toward OER.

The first copper­(II) complex reported for water oxidation electrocatalysis, [(bpy)­Cu­(μ-OH)]_2_^2+^, operated at high turnover frequency, 100 s^–1^, but at a considerably high overpotential, 750 mV, at a current density of 1 mA cm^–2^ at pH 12.5.^2^ Since then, molecular copper complexes with different ligands have been described for both electrocatalytic OER and hydrogen evolution reaction (HER).^3–15^ Some important features of the copper complexes are diverse redox properties, well-defined coordination chemistry, low cost, high earth abundance, and relatively low toxicity to the environment.

Bridging ligands based on pyridine-carboxylate groups have been extensively used in the synthesis of copper­(II) coordination polymers.^16–21^ This type of ligand has properties similar to those of the amino acid residues present in natural OEC and can participate in redox steps and improve the catalytic efficiency of multielectron reactions.^22^ The chemistry of the coordination polymers is of great interest because of the structural diversity and unique functionalities such as host–guest chemistry, magnetism, electronic conductivity, and optical property. The one-dimensional copper­(II) coordination polymer has been described as a precatalyst due to the structural transformation that occurs during OER. This in situ transformation leads to the formation of metal oxides or hydroxides, which become the active catalysts.^23^ The one-dimensional copper­(II) coordination polymer, based on paddle-wheel units connected through phosphine dioxide ligands, was described as a heterogeneous electrocatalyst for water oxidation, exhibiting an onset overpotential of 509 mV at a current density of 0.1 mA cm^–2^ at pH 10.2 and an overpotential of 563 mV at a current density of 1 mA cm^–2^.^24^ The copper­(II) coordination polymer with imidazolate derivatives as ligands delivered overpotentials of 500, 520, and 560 mV in OER for Cu-imidazole, Cu-2-methylimidazole, and Cu-benzimidazole, respectively, at pH 14 at 10 mA cm^–2^.^25^ Although there has been progress in the area, challenges remain toward the development of efficient, stable, and water-soluble molecular catalysts.

In the present work, we investigated the activity of a new amino-acid-based copper­(II) coordination polymer with the ligand *N*-(2-pyridylmethyl)­ethylenediamine-*N*-propanoate (PEP) for water oxidation electrocatalysis. The ligand design was inspired by the OEC in PSII, with the pyridine- and carboxylate-based tetradentate ligand resembling the histidine and glutamic acid residues. Furthermore, coordination sites with different Lewis basic character can infer structural stability while at the same time modulating the electronic structure of the catalytic site and allowing redox processes to occur at the Cu­(II) center. The introduction of a *N*-propanoate linker to the ligand was envisioned to allow structural flexibility for the formation of carboxylate bridges and the coordination polymer. The single-crystal structure of the coordination polymer {[CuPEP]­ClO_4_}_*n*_ shows a one-dimensional array, whose carboxylate group bridges the copper­(II) centers in an unusual monatomic bridge involving chelation. The coordination polymer is soluble in water, and the OER electrocatalytic activity was explored in a phosphate buffer at pH 12.5. An onset overpotential of 394 mV was achieved at a current density of 0.1 mA cm^–2^, which is comparable to other copper­(II) molecular catalysts reported in the literature. Stability tests were conducted and revealed the formation of a CuOx-OH film at the working electrode surface, indicating the contribution of heterogeneous electrocatalysis for the OER.

## 2 Experimental Section

### 2.1 Synthesis of the Ligand NaPEP (Sodium *N*-(2-Pyridylmethyl)­ethylenediamine-*N*-propanoate)

#### 2.1.1 Synthesis of *N*-*tert*-Butoxycarbonylethylenediamine (**1**)

To a solution of 1,2-ethylenediamine (0.86 mol, 57.41 mL) in 160 mL of THF and 20 mL of H_2_O under ice bath, 0.107 mol of Boc_2_O (23.45 g) in 400 mL of THF was slowly added in a rate of roughly 3.3 mL h^–1^. Afterward, the reaction proceeded for another 48 h at room temperature. The solvent was removed in a rotatory evaporator, and 200 mL of H_2_O was added to the residue, forming a white precipitate from the diprotected 1,2-ethylenediamine. The solid was filtered under reduced pressure, and the aqueous phase was extracted with 10 × 50 mL of CH_2_Cl_2_. The organic phase was dried over anhydrous MgSO_4_ and filtered, and the solvent was removed in a rotatory evaporator and then in an alto-vacuum pump to produce a yellowish viscous oil. Yield: 14.43 g (84%).

^1^H NMR (200 MHz, CDCl_3_), δ (ppm): 3.11–3.20 (qr, 2H, −*CH*_*2*_N-Boc); 2.75–2.81 (t, 2H, H_2_N-*CH*_*2*_CH_2_N-Boc); 1.43 (s, 9H, −*CH*_3(Boc)_). ^13^C­{^1^H} NMR (50 MHz, CDCl_3_), δ (ppm): 156.3 (1C, −*C=O*_(Boc)_); 79.3 (1C, −*C*(CH_3_)_(Boc)_); 43.4 (1C, H_2_N*CH*_*2*_CH_2_N-Boc); 41.9 (1C, −*CH*_*2*_N-Boc); 28.5 (3C, −*CH*_3(Boc)_). FTIR (film, cm^–1^): 3361, 3307, 3047, 3004, 2977, 2933, 2872, 1694, 1525, 1455, 1392, 1366, 1252, 1172, 956. Spectra are shown in Figure S1.

#### 2.1.2 Synthesis of *N*-*tert*-Butoxycarbonyl-*N*′-(2-pyridylmethyl)­ethylenediamine (**2**)

To a solution of **1** (90.1 mmol, 14.41 g) in methanol (40 mL) was added an equimolar amount of 2-pyridinecarboxaldehyde (8.60 mL) under stirring at room temperature. After 1 h, NaBH_4_ (120 mmol, 4.54 g) was slowly added over a period of 2 h, and the reaction was stirred for another 2 h. The solvent was then evaporated, and the residue was treated with saturated aqueous NH_4_Cl. The aqueous phase was extracted with CHCl_3_ (4 × 50 mL), the combined organic layers were dried over anhydrous MgSO_4_ and filtered, and the solvent was removed. The product was obtained as a yellowish oil. The reaction was followed by thin-layer chromatography (TLC), eluted with *n*-hexane/EtOAc (1:1); *R*_f_ = 0.10. Yield: 22.39 g (99%).

^1^H NMR (200 MHz, CDCl_3_), δ (ppm): 8.52–8.54 (d, 1H, H_6‑Py_); 7.58–7.66 (t, 1H, H_4‑Py_); 7.24–7.27 (d, 1H, H_3‑Py_); 7.11–7.17 (t, 1H, H_5‑Py_); 3.88 (s, 2H, −N*CH*_*2*_Py); 3.19–3.27 (qr, 2H, −*CH*_*2*_N-Boc); 2.73–2.79 (t, 2H, −N-*CH*_*2*_CH_2_N-Boc); 1.42 (s, 9H, −*CH*_3(Boc)_). ^13^C­{^1^H} NMR (50 MHz, CDCl_3_), δ (ppm): 159.1 (1C, C_2‑Py_); 156.2 (1C, −*C=O*_(Boc)_); 149.3 (1C, C_6‑Py_); 136.6 (1C, C_4‑Py_); 122.1–122.4 (2C, C_3‑Py_ and C_5‑Py_); 79.2 (1C, −*C*(CH_3_)_(Boc)_); 54.5 (1C, −N*CH*_*2*_Py); 48.8 (1C, −N*CH*_*2*_CH_2_N-Boc); 40.3 (1C, −*CH*_*2*_N-Boc); 28.4 (3C, −*CH*_3(Boc)_). FTIR (film, cm^–1^): 3329, 3052, 3006, 2976, 2932, 1701, 1593, 1571, 1523, 1477, 1435, 1392, 1366, 1252, 1172, 998, 760. Spectra are shown in Figure S2.

#### 2.1.3 Synthesis of *N*-*tert*-Butoxycarbonyl-*N*′-(2-pyridylmethyl)­ethylenediamine-*N*′-methylpropanoate (**3**)

A solution of **2** (89.01 mmol, 22.34 g) in methanol (30 mL) and an excess of methyl acrylate (267.0 mmol, 24.07 mL) were refluxed under stirring for 8 h. The solvent and residual methyl acrylate were evaporated, and the product was obtained as a brown viscous oil without further purification. The reaction was followed by TLC, eluted with MeOH/*n*-hexane/EtOAc (1:2:2); *R*_f_ = 0.75. Yield: 30.00 g (100%).

^1^H NMR (200 MHz, CDCl_3_), δ (ppm): 8.52–8.54 (d, 1H, H_6‑Py_); 7.63–7.70 (t, 1H, H_4‑Py_); 7.30–7.38 (d, 1H, H_3‑Py_); 7.15–7.21 (t, 1H, H_5‑Py_); 3.78 (s, 2H, −N*CH*_*2*_Py); 3.69 (s, 3H, −CO_2_ *CH*_*3*_); 3.16–3.24 (qr, 2H, −*CH*_*2*_N-Boc); 2.87–2.94 (t, 2H, −N*CH*_*2*_CH_2_CO_2_CH_3_); 2.60–2.66 (t, 2H, −N*CH*_*2*_CH_2_N-Boc); 2.49–2.55 (t, 2H, −*CH*_*2*_CO_2_CH_3_); 1.44 (s, 9H, −*CH*_3(Boc)_). ^13^C­{^1^H} NMR (50 MHz, CDCl_3_), δ (ppm): 173.0 (1C, −*CO*_*2*_CH_3_); 159.2 (1C, C_2‑Py_); 156.1 (1C, −*C=O*_(Boc)_); 149.1 (1C, C_6‑Py_); 136.5 (1C, C_4‑Py_); 123.0 (1C, C_3‑Py_); 122.2 (1C, C_5‑Py_); 78.9 (1C, −*C*(CH_3_)_(Boc)_); 60.0 (1C, −N*CH*_*2*_Py); 53.4 (1C, −*CH*_*2*_CO_2_CH_3_); 51.6 (1C, −CO_2_ *CH*_*3*_); 49.7 (1C, −N*CH*_*2*_CH_2_N-Boc); 38.3 (1C, −*CH*_*2*_N-Boc); 32.7 (1C, −N*CH*_*2*_CH_2_CO_2_CH_3_); 28.5 (3C, −*CH*_3(Boc)_). FTIR (film, cm^–1^): 3388, 3052, 3003, 2976, 2952, 2933, 2827, 1736, 1712, 1591, 1570, 1514, 1475, 1456, 1436, 1391, 1365, 1250, 1174, 995, 760. Spectra are shown in Figure S3.

#### 2.1.4 Synthesis of the Ligand Sodium *N*-(2-Pyridylmethyl)­ethylenediamine-*N*-propanoate (NaPEP, **4**)

A solution of **3** (88.7 mmol, 29.90 g) in 200 mL of ethanol/HCl_(c)_ (1:1) was stirred for 24 h at room temperature. The ethanol was evaporated, and the resulting aqueous phase was reacted with NaOH_(s)_ until pH 10. The water was removed under reduced pressure, and the product was extracted from the solid residue several times with 50 mL of a 1:1 CHCl_3_/MeOH solution. The combined organic layers were dried over anhydrous MgSO_4_, the solvent was removed, and the product was obtained as a brown viscous oil. The reaction was followed by TLC, eluted with MeOH/*n*-hexane/EtOAc (1:2:2); *R*_f_ = 0.21. Yield: 17.29 g (83%). The global yield based on Boc_2_O was 66%.

^1^H NMR (200 MHz, D_2_O), δ (ppm): 8.62–8.65 (d, 1H, H_6‑Py_); 8.31–8.39 (t, 1H, H_4‑Py_); 7.78–7.87 (m, 2H, H_5‑Py_ and H_3‑Py_); 4.24 (s, 2H, −N*CH*_*2*_Py); 3.22–3.28 (t, 2H, −*CH*_*2*_CH_2_NH_2_); 3.04–3.10 (t, 2H, −N*CH*_*2*_CH_2_CO_2_^–^); 2.95–3.02 (t, 2H, −*CH*_*2*_NH_2_); 2.45–2.52 (t, 2H, −*CH*_*2*_CO_2_^–^). ^13^C­{^1^H} NMR (50 MHz, CD_3_OD), δ (ppm): 181.3 (1C, −*CO*_*2*_^–^); 160.9 (1C, C_2‑Py_); 149.4 (1C, C_6‑Py_); 138.8 (1C, C_4‑Py_); 125.1 (1C, C_3‑Py_); 123.8 (1C, C_5‑Py_); 60.8 (1C, −N*CH*_*2*_Py); 55.0 (1C, −*CH*_*2*_CO_2_^–^); 51.9 (1C, −N*CH*_*2*_CH_2_NH_2_); 39.2 (1C, −*CH*_*2*_NH_2_); 36.8 (1C, −N*CH*_*2*_CH_2_CO_2_^–^). FTIR (film, cm^–1^): 3354, 3285, 3062, 3009, 2942, 2837, 1591, 1580 (broad), 1570, 1474, 1434, 1403, 760. Spectra are shown in Figure S4.

### 2.2 Synthesis of {[CuPEP]­ClO_4_}_*n*_

The coordination polymer was synthesized from the reaction of an ethanolic solution of the ligand NaPEP (0.5 mmol, 0.13 g) with an ethanolic solution of Cu­(ClO_4_)_2_·6H_2_O (0.5 mmol, 0.19 g) at room temperature and under stirring. A thin blue solid precipitated, and after 1 h of stirring, the reaction mixture was kept at −5 °C for 1 day. The solid was filtered off, resulting in 0.12 g of a blue powder. Blue single crystals of the coordination polymer suitable for X-ray analysis were obtained from recrystallization in CH_3_CN/CH_2_Cl_2_/H_2_O. When the coordination polymer was not kept at a low temperature before the filtration, a colloidal solid was obtained that formed a film after drying. Further analysis showed that the film and the powder were the same compound. Yield: 0.118 g (61%).

Anal. for C_11_H_16_N_3_O_6_Cl_1_Cu·1H_2_O: C, 32.76; H, 4.50; N, 10.42. Found: C, 32.81; H, 4.11; N, 10.07. FTIR (CsI pellet, cm^–1^): 3342, 3296, 3182, 3075, 2988, 2961, 2896, 1609, 1599, 1542, 1481, 1435, 1385, 1317, 1100, 774. UV–Vis in CH_3_CN; λ/nm (ε/dm^3^ mol^–1^ cm^–1^): 258 (7.83 × 10^3^); 284 (3.00 × 10^3^); 706 (1.31 × 10^2^). Λ_M_ (CH_3_CN) = 129.5 Ω^–1^ cm^2^ mol^–1^.

### 2.3 Electrochemical Measurements

The electrochemical experiments were carried out using an Autolab PGSTAT302N or an Autolab PGSTAT204potentiostat/galvanostat (Metrohm, Switzerland), controlled by the NOVA software (Metrohm), using a three-electrode system composed of a platinum auxiliary electrode, Ag|AgCl (3.0 mol L^–1^ KCl) as a reference electrode for buffer and Pt as pseudoreference electrode for acetonitrile solutions, and a glassy carbon disk (area 0.0314 cm^2^) or FTO (area 1 cm^2^) as a working electrode, at 25 °C. Electrochemical data were collected in acetonitrile using 0.1 mol L^–1^ tetrabutylammonium perchlorate as a supporting electrolyte or at 0.1 mol L^–1^ phosphate-buffered solutions at pH 12.5 using 1.0 × 10^–3^ mol L^–1^ of catalyst under an argon atmosphere. Measured potentials were adjusted to NHE by adding 0.210 V for buffered solutions or were adjusted by the use of ferrocene as an internal standard (0.4 V vs NHE) in acetonitrile.^26^ All cyclic voltammograms are plotted according to the IUPAC convention.

Fluorine tin oxide (FTO) glass plates with 13 Ω per square surface resistivity were purchased from Sigma-Aldrich. FTO glass plates were previously cut into slides of 1 × 3.5 cm (*W* × *H*). Before use, the FTO slides were first sonicated in soap water then in ethanol and acetone for 10 min and finally rinsed with deionized water before use. The glassy carbon electrodes were polished before use using a water paste of alumina (MicroPolish, Buehler) of 1.0 and 0.05 μm over a polishing cloth (Microcloth, Buehler).

OER electrocatalytic tests were conducted using FTO glass plate of 1 cm^2^ active area as a working electrode at 0.1 mol L^–1^ phosphate-buffered solutions at pH 12.5, using 5 × 10^–3^ mol L^–1^ of catalyst under an argon atmosphere, at 25 °C. Electrocatalytic activity for OER was assessed by cyclic voltammetry. All cyclic voltammograms are plotted according to the IUPAC convention, and the start point and sweep direction are indicated in all CVs by arrows.

The applied potential was expressed versus the normal hydrogen electrode (*E*_NHE_) according to eq 1, and the overpotential (η) for the OER was calculated according to eq 2.^27^

$$E_{\mathrm{NHE}}=E_{\mathrm{Ag}|\mathrm{AgCl}}+0.210\;V$$ 1

$$\eta =E_{\mathrm{NHE}}-(1.23\;V-0.059\;V\times \mathrm{pH})$$ 2

Stability tests were conducted by two methods, cyclic voltammetry and chronopotentiometry, under the same conditions of the OER tests. For the CV, 100 cycles at 100 mV s^–1^ were carried out in two potential windows: −1.25 to 1.74 V vs NHE and −0.44 to 1.44 V vs NHE. The chronopotentiometry was carried out at a current density of 5 mA cm^–2^. Faradaic efficiency was evaluated using the same experimental procedure in the first 3 h of chronopotentiometry using a dissolved oxygen probe in a sealed cell.

## 3 Results and Discussion

### 3.1 Synthesis and Structural Characterization of {[CuPEP]­ClO_4_}_*n*_

The new ligand NaPEP was synthesized through a four-step route, as shown in Scheme 1. The first step consisted of the monoprotection of 1,2-ethylenediamine with Boc_2_O to form **1**, as described in the literature.^28,29^ However, it used a more diluted Boc_2_O solution and a longer addition time into the 1,2-ethylenediamine solution to reach 84% yield. The reductive amination of **1** with 2-pyridinecarboxaldehyde in the presence of NaBH_4_ afforded **2** with 99% yield. The synthesis of **2** was previously described using NaH_3_B­(CN)/NaHCO_3_, with 27% yield.^30^ Here, the yield increased considerably by following a different procedure.^31^ In the third step, a Michael reaction of **2** and methyl acrylate leads to the formation of **3**. The experimental procedure of this synthesis was based on a published work;^32^ however, a modification on the reaction temperature, from room temperature to reflux, improved the reaction time from 1 week to 8 h. The last step consisted of the deprotection of the Boc group in HCl/ethanol solution.^33^ In this step, the methyl ester group was also hydrolyzed, and after treatment with NaOH and extraction with a methanol/chloroform solution, the sodium salt of the ligand, NaPEP (**4**), was obtained with 83% yield and 66% global yield in relation to Boc_2_O.

**Scheme 1:**
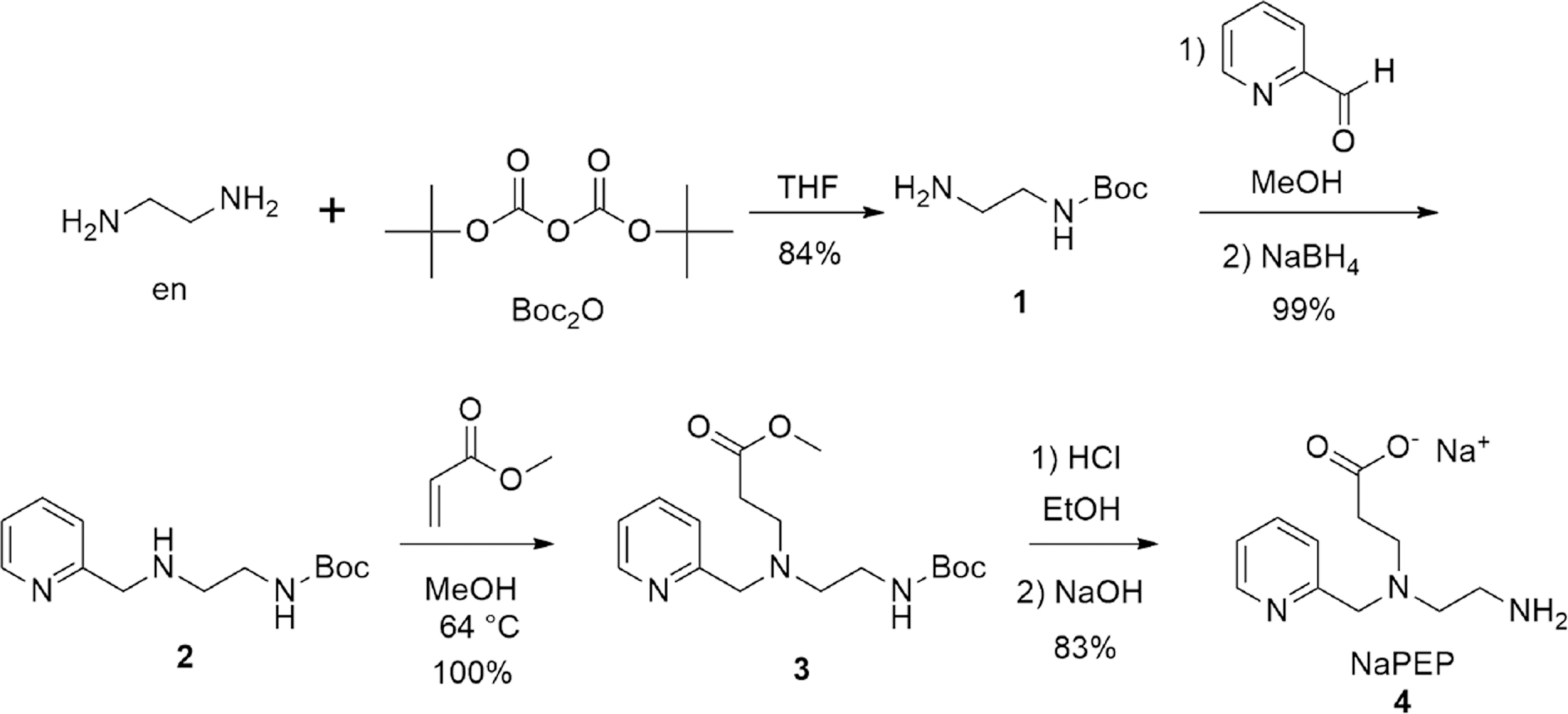
Synthetic Route of the Ligand NaPEP

The self-assembling reaction of Cu­(ClO_4_)_2_·6H_2_O and NaPEP shown in Scheme 2 produced the coordination polymer {[CuPEP]­ClO_4_}_*n*_ with carboxylate bridges linking the Cu^II^ ions in a one-dimensional array. Attempts to synthesize a complex starting from CuCl_2_·2H_2_O and NaPEP produced a green solid that decomposed after filtration, which upon addition of NaClO_4_ to the reaction also produced {[CuPEP]­ClO_4_}_*n*_, indicating that a larger counterion is necessary to stabilize the coordination polymer.

**Scheme 2:**
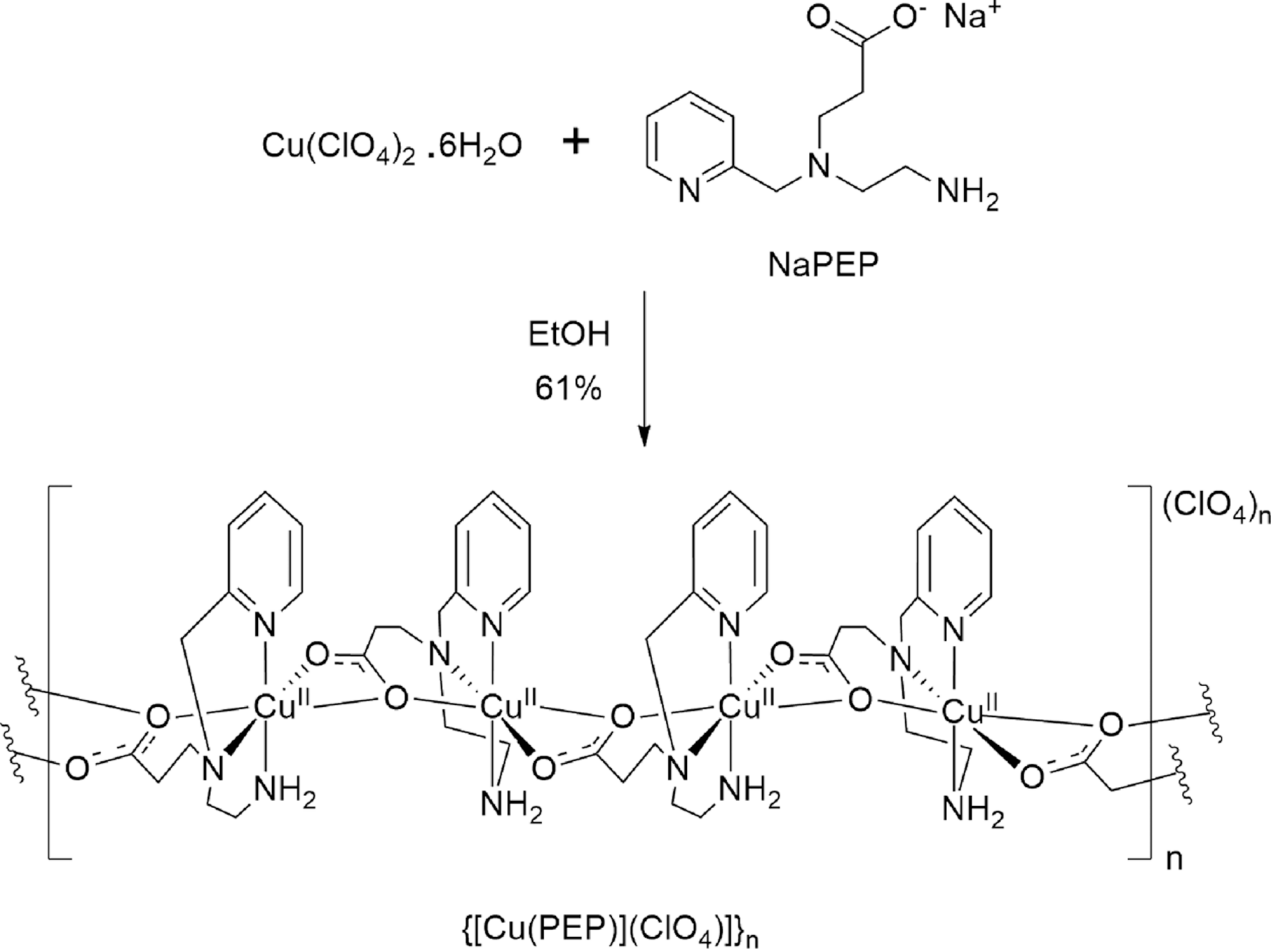
Synthesis of the Coordination Polymer {[CuPEP]­ClO_4_}_*n*_

#### 3.1.1 Single-Crystal and Polycrystalline X-ray Diffraction

X-ray crystallography analysis revealed a one-dimensional coordination polymer with one perchlorate counterion per Cu^II^ unit. The ORTEP plot and 1D polymeric chain are shown in Figures 1 and 2, respectively. Selected bond lengths and angles are listed in Table 1.

**Figure 1:**
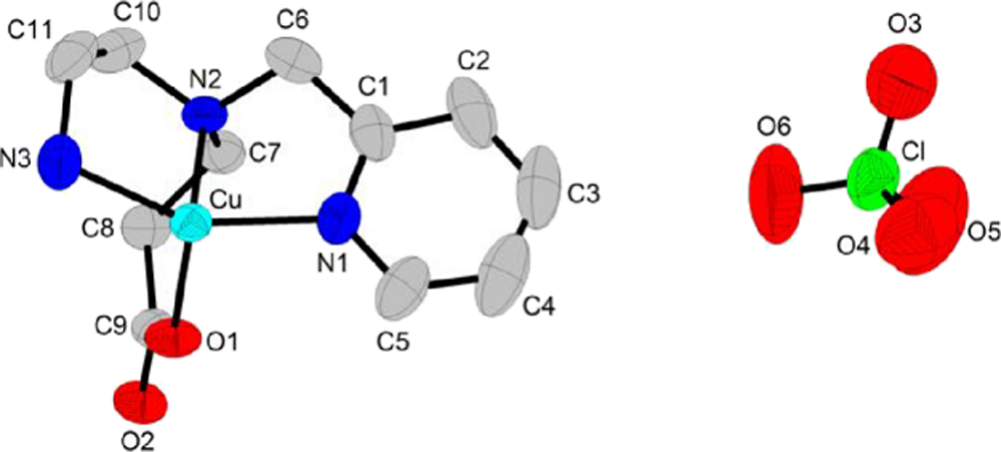
ORTEP plot of the asymmetric unit of {[CuPEP]­ClO_4_}_*n*_. Thermal ellipsoids are shown at 50% probability level. Hydrogen atoms were omitted for clarity.

**Figure 2:**
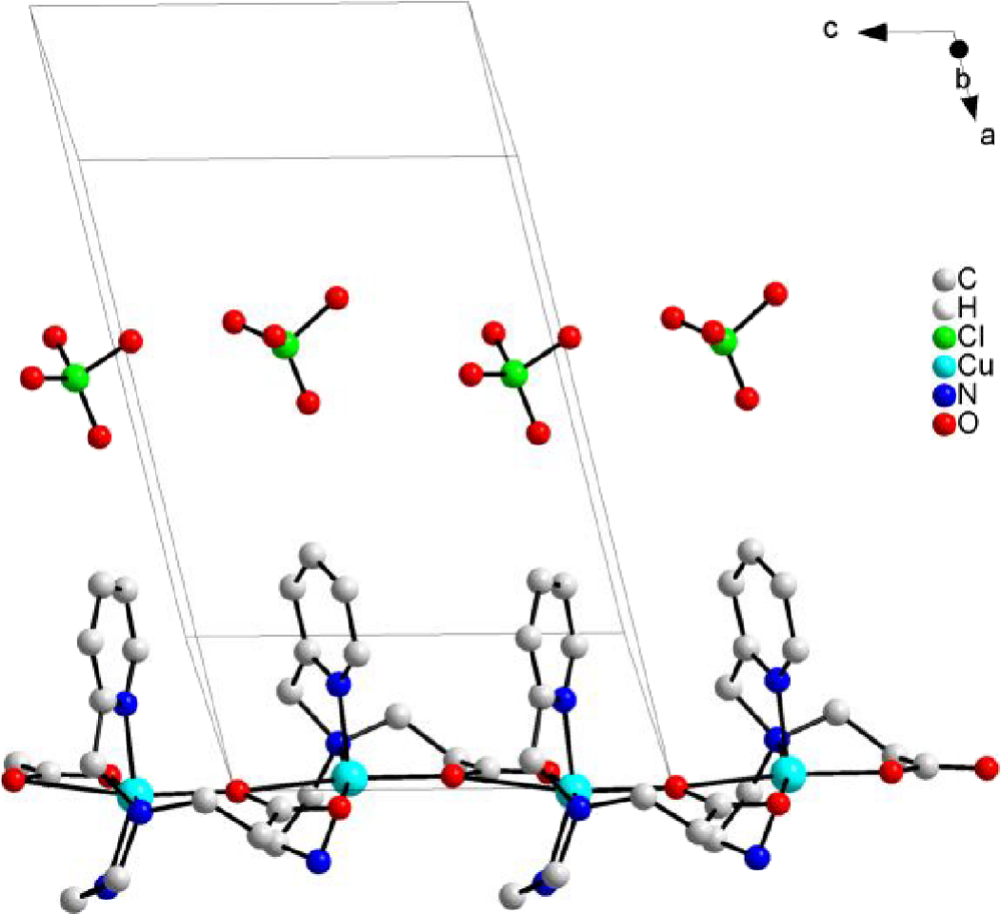
One-dimensional polymeric chain of {[CuPEP]­ClO_4_}_*n*_ along the [001] crystallographic direction.

**Table 1:** Selected Bond Lengths (Å) and Angles (°) for {[CuPEP]­ClO_4_}*n* ^a^

bond lengths		bond angles			
O2–Cu#2	1.979(2)	O2#1–Cu–N3	93.13(13)	N3–Cu–O1	108.81(15)
Cu–O1#1	2.696(1)	O2#1–Cu–N1	94.45(11)	N1–Cu–O1	95.82(13)
Cu–N3	2.006(3)	N3–Cu–N1	152.73(15)	N2–Cu–O1	93.66(12)
Cu–N1	2.023(3)	O2#1–Cu–N2	167.19(12)	C9–O1–Cu	127.4(2)
Cu–N2	2.061(2)	N3–Cu–N2	85.09(15)	Cu–O1–Cu#2	156.75(1)
Cu–O1	2.154(2)	N1–Cu–N2	81.76(13)	O1#1–Cu-O1	152.80(1)
C9–O1	1.259(4)	O2#1–Cu–O1	98.92(8)	C9–O2–Cu#2	108.16(18)
C9–O2	1.277(4)	O2#1–Cu–O1#1	54.17(0)	O1–C9–O2	122.4(3)

a Symmetry transformations used to generate equivalent atoms: {[CuPEP]­ClO_4_}_*n*_: #1 *x*, −*y* + 2, *z* + 1/2; #2 *x*, −*y* + 2, *z* – 1/2.

In {[CuPEP]­ClO_4_}_n_, each Cu^II^ ion is meridionally coordinated to the three nitrogen atoms of the ligand, forming two five-membered chelate rings. The carboxylate O1 is coordinated intramolecularly to Cu^II^ in monodentate mode (I, Scheme S1) and forms a six-membered chelate ring. Additionally, both oxygen atoms (O1 and O2) are intermolecularly coordinated to the adjacent Cu^II^ atom and form a chelate (II, Scheme S1), growing the polymeric chain along the crystallographic axis *c*, as represented in Scheme S2. The coordination mode of the carboxylate in relation to both Cu^II^ ions can be described as a monatomic bridge involving chelation (VI, Scheme S1). Briefly, each molecule of the ligand PEP acts as a tetradentate ligand toward the intramolecular Cu^II^ and bidentate toward the neighbor Cu^II^ center.

Copper­(II) can be considered as six-coordinated with a tetragonal elongated geometry due to Jahn–Teller distortion, typical of a d^9^-Cu^II^ complex. The equatorial plane comprises the three nitrogen atoms and the O2 atom from the neighbor center. Both intra- and intermolecular O1 atoms are in the axial positions. The average of the bond lengths in the equatorial positions is 2.017 Å, while the bonds in the axial positions are 2.154(2) Å (intramolecular Cu-O1) and 2.696(1) Å (intermolecular Cu–O1). The latter is a very weak bond, but its length is still in the range of tetragonal structures.^34^ Some authors also described these complexes as pentacoordinated with square pyramidal geometries.^35^ The distortion around the Cu^II^ ion is also evidenced by the wide range of angles from 54.17 to 108.81°. The smallest is the bite angle of the carboxylate group to the adjacent Cu^II^ ion, O1–Cu–O2.

In the equatorial plane, the pyridine is *trans* to the primary amine and the tertiary amine is *trans* to the oxygen O2 and presented similar Cu–N bond lengths (Table 1). The close values of the C–O bonds of the carboxylate group (C9–O1 1.259(4) Å; C9–O2 1.277(4) Å) are typical of bridges, where the double bond is delocalized between the two C–O single bonds. Complexes with carboxylate in monodentate mode present a larger difference between these bonds, indicating localization of the double bond. For the mononuclear iron­(III) complex with monodentate mode [Fe­(PBMPA)­Cl_2_] (PBMPA: *N*-propanoate-*N*,*N*-bis­(2-pyridylmethyl)­amine),^32^ the difference is 0.043 Å, while for {[CuPEP]­ClO_4_}_*n*_, this difference is only 0.018 Å. The deviation of the angles Cu–O1–Cu (156.75 °) and O1–Cu–O1 (−152.80 °) from 180 ° results in a wave-like structure for the ···Cu–O–Cu–O-Cu··· core (Figure S5). In consequence, the pyridine rings are not coplanar to each other in the polymeric chain.

The hydrogen bonds formed in {[CuPEP]­ClO_4_}_*n*_ are presented in Figure S6, and the geometric parameters are shown in Table S2. Oxygen atoms of the perchlorate ions interact with both the NH_2_ group and the C–H bonds of the pyridine ring. From Figure S7 it is possible to observe the polymeric chain packing along the crystallographic axis *c*. Each group of four polymeric chains forms a hole in the center that is filled with perchlorate ions. The hydrogen bonds as well as the electrostatic interactions orient the perchlorate ions in such cavities and consequently obstruct them. Figure 3 shows a perspective view of the tubes formed along the *c* axis.

**Figure 3:**
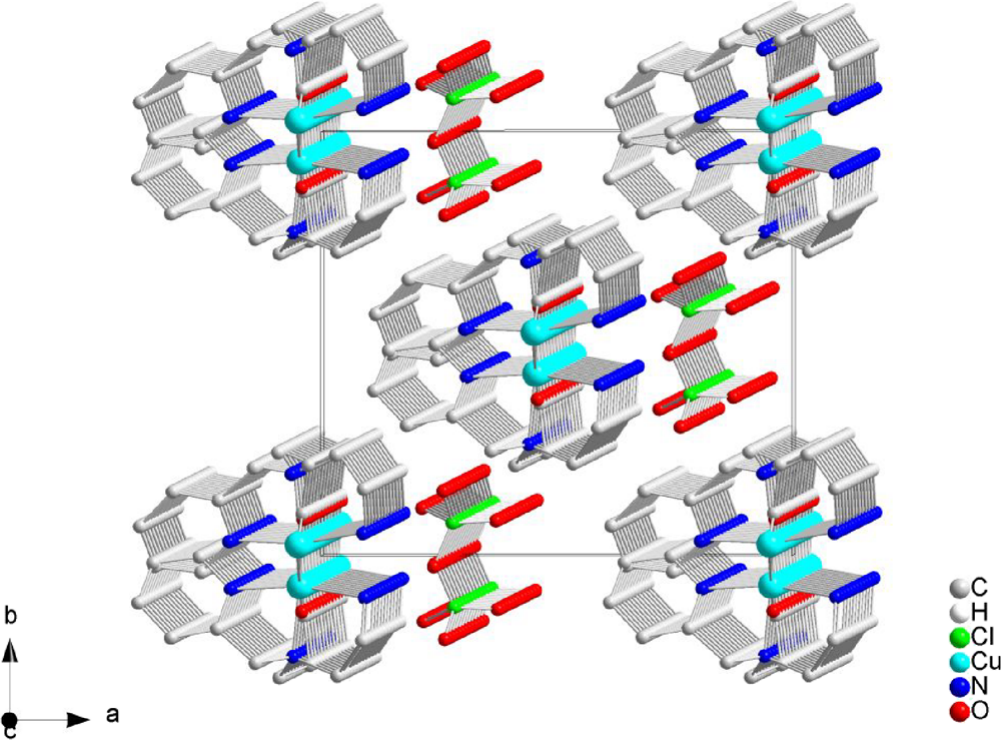
View of the tubes formed by packing of the polymeric chain of {[CuPEP]­ClO_4_}_*n*_.

One-dimensional copper­(II) coordination polymers with similar structures have been described with ligands possessing a pyridyl group as well as carboxylate pendant armies. Table S3 summarizes some important crystallographic data for these compounds. The coordination mode of the carboxylate to the metal seems to be related to the size of the carboxylate army. Carboxylate with one (*n* = 1) or two (*n* = 2) methylene groups usually bridges the two Cu^II^ centers through a *syn–anti* bridge (III, Scheme S1). With longer methylene chains (*n* = 3, 4, or 5), only one oxygen from the carboxylate coordinates to the adjacent copper atom, while the second oxygen remains uncoordinated, i.e., monodentate mode (I, Scheme S1). The Cu···Cu distance reflects such a behavior. In the *syn–anti* bridge, the Cu···Cu distance is around 4.5–5.8 Å, while in monodentate mode, the Cu···Cu distance stays in the range of 9.3–10.4 Å. All of these previously reported coordination polymers possess the copper center pentacoordinated and are involved in a distorted square-pyramid geometry, different from the tetragonal elongated geometry of {[CuPEP]­ClO_4_}_*n*_. In the case of the monodentate carboxylate groups, a water molecule or a perchlorate ion occupies the fifth position of the square pyramidal copper center.

The copper­(II) coordination polymer {[Cu­(L^3^OO)]­[CF_3_SO_3_]}_*n*_·H_2_O (L^3^OO^–^: 3-[(2-(pyridine-2-yl)­ethyl)­{(dimethylamino)­ethyl}­amino]­propionate) possesses a very similar ligand to PEP, where a −N­(CH_3_)_2_ group is present instead of the −NH_2_ of PEP.^36^ Despite this similarity, the coordination polymers present some differences on the geometry. {[Cu­(L^3^OO)]­[CF_3_SO_3_]}_*n*_·H_2_O possesses a square-pyramidal geometry, while {[CuPEP]­ClO_4_}_*n*_ is tetragonal elongated. The Cu^II^ ion in {[CuPEP]­ClO_4_}_*n*_ is additionally coordinated to a carboxylate oxygen intermolecularly, completing the sixth coordination position. The Cu···Cu distance in {[Cu­(L^3^OO)]­[CF_3_SO_3_]}_*n*_·H_2_O (5.281 Å) is considerably longer than that in {[CuPEP]­ClO_4_}_*n*_ (4.751 Å). Nevertheless, the Cu–O1 intermolecular bond in {[CuPEP]­ClO_4_}_*n*_ is 2.696 Å, while in {[Cu­(L^3^OO)]­[CF_3_SO_3_]}_*n*_·H_2_O, the analogous unbounded distance is 3.137 Å. Among these coordination polymers, {[CuPEP]­ClO_4_}_*n*_ is the only one with tetragonal elongated geometry and also shows one of the smallest Cu···Cu distances, 4.751(1) Å, which is coherent with a monatomic bridging involving chelation.

The XRD polycrystalline diffraction pattern of {[CuPEP]­ClO_4_}_*n*_ is shown in Figure S8. The diffractogram calculated by the Rietveld method from a single crystal is shown in Figure S9 and the result of the refinement in shown in Table S4. Both diffractograms are equivalent, allowing a well-defined qualitative characterization that shows 100% purity in the powder sample. For indexing, a pseudo-Voigt peak profile function was used. The refinement of the peaks shows the equivalence between both primitive cells and the overlapping of all diffraction peaks in both experimental and calculated diffraction patterns.

#### 3.1.2 FTIR Spectroscopy

The FTIR spectrum (Figure 4A, Figure S10, Table S5) of {[CuPEP]­ClO_4_}_*n*_ presents the C=C and C=N vibrations in the region of 1609–1435 cm^–1^, characteristic of the coordinated pyridine ring. The difference Δν_CO2_ (ν_as_ – ν_as_) between the symmetric (ν_s_) and asymmetric (ν_as_) stretching of the carboxylate group (1542 – 1385 = 157 cm^–1^) is smaller than in the free carboxylate ligand (1591 – 1403 = 188 cm^–1^), and it is characteristic of a coordination in monatomic bridging involving the chelation mode (VI, Scheme S1),^37,38^ in agreement with the crystal structure. The perchlorate counterion is observed around 1100 cm^–1^.

**Figure 4:**
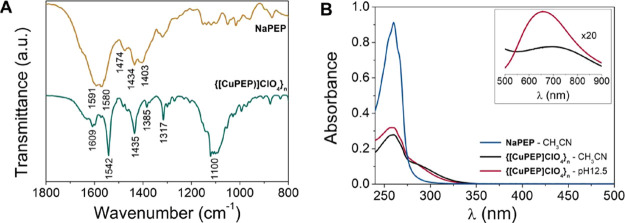
(A) FTIR spectra of the ligand NaPEP and the coordination polymer {[CuPEP]­ClO_4_}_*n*_ in KBr. (B) UV–vis spectrum of {[CuPEP]­ClO_4_}_*n*_ at 1 × 10^–5^ mol L^–1^ in CH_3_CN and aqueous buffered solution at pH 12.5 (inlet: 5 × 10^–3^ mol L^–1^).

#### 3.1.3 UV–Vis Electronic Spectroscopy

The electronic spectra of {[CuPEP]­ClO_4_}_*n*_ in CH_3_CN (Figure 4B, Figure S11) shows a band at 258 nm characteristic of the ligand-centered (LC) π → π* transition of the pyridine ring of NaPEP and a band around 284 nm that can be attributed to metal to ligand charge transfer (MLCT).^39^ The metal-centered transition (MC) T_2g_ ← E_g_ was observed as a weak band in the visible region at 706 nm^39^. The UV–vis spectrum of {[CuPEP]­ClO_4_}_n_ acquired in buffer at pH 12.5 (Figure 4B, inset) is qualitatively similar to that in acetonitrile, and no difference in the λ_max_ was observed for the LC and MLCT transitions. The MC transition at pH 12.5 is blue-shifted compared to CH_3_CN because of the solvatochromic effect. Water (18.0) presents a higher Gutmann’s donor number than CH_3_CN (14.1);^40^ thus, it increases the basicity of the ligand and consequently the MC energy.

#### 3.1.4 ESI-MS

The ESI-MS spectrum of {[CuPEP]­ClO_4_}_*n*_ was acquired in positive ion mode and is shown in Figure S12, together with the assignment to the corresponding peaks. The spectrum is characterized by the presence of monomeric (*m*/*z* 285.0527) and dimeric (*m*/*z* 671.0523) species formed from the dissociation of the polymeric chain during the analysis. The most abundant monomeric species represents the minor integer fragment of the polymeric chain, without suffering any fragmentation. In the protonated dimeric species, the copper center was reduced to Cu^I^, a common process in transition metal coordination compound electrospray analysis.^41^ The presence of the dimeric species corroborates the polymeric structure found in the single-crystal X-ray analysis. Both monomeric and dimeric species possess unsaturated Cu^II^ centers that could bind to water in the OER electrocatalysis.

### 3.2 Cyclic Voltammetry

First, the electrochemical properties of {[CuPEP]­ClO_4_}_*n*_ were investigated by cyclic voltammetry in CH_3_CN, where two electrochemical events occur (Figure 5). Process **a** shows only the anodic peak potential at *E*_pa_(a) = +1.50 V vs NHE at 100 mV s^–1^, assigned to the Cu^II^/Cu^III^ redox couple, typical of an electrochemical irreversible process (eq S2). The high current observed for peak **a** was investigated to check if this was associated with the decomposition of the coordination polymer. CVs with anodic or cathodic sweep show the same behavior (Figure S13), indicating no degradation of {[CuPEP]­ClO_4_}_*n*_. The high current is probably from moisture in CH_3_CN and a contribution from both the Cu^II^/Cu^III^ redox couple and the OER catalytic process.

**Figure 5:**
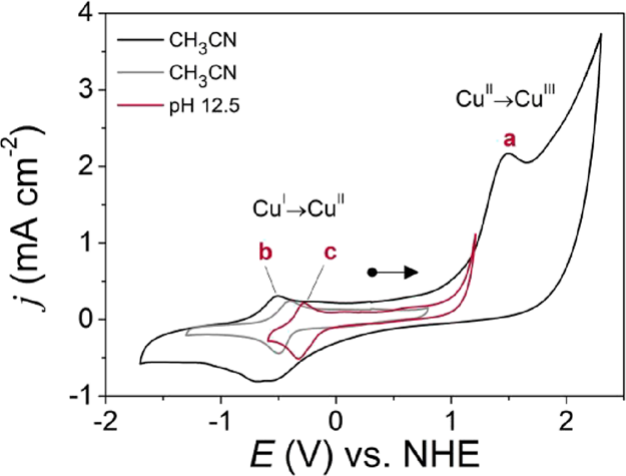
Cyclic voltammograms of {[CuPEP]­ClO_4_}_*n*_ at 1 × 10^–3^ mol L^–1^ and 100 mV s^–1^ in CH_3_CN (black and gray curves measured in different potential window) and phosphate buffer at pH 12.5.

Peak **a** was investigated in varying scan rates (Figure S14, Table S6). As shown in Figure 6A, the variation of *E*_pa_(a) with the scan rate is typical of an irreversible process. The *i*_pa_ vs *v* ^1/2^ plot (Figure 6B) is linear and suggests a diffusion-controlled process governed by the Randles–Sevcik equation (eq S5), with the electrochemically active species freely diffusing in solution^42,43^ and with a diffusion coefficient *D* of 7.96 × 10^–4^ cm^2^ s^–1^. This is confirmed by the log­(*i*) vs log­(*v*) plot that gives a slope of 0.6 (Figure 6C), close to the theoretical slope for reaction controlled by diffusion (0.5).

**Figure 6:**
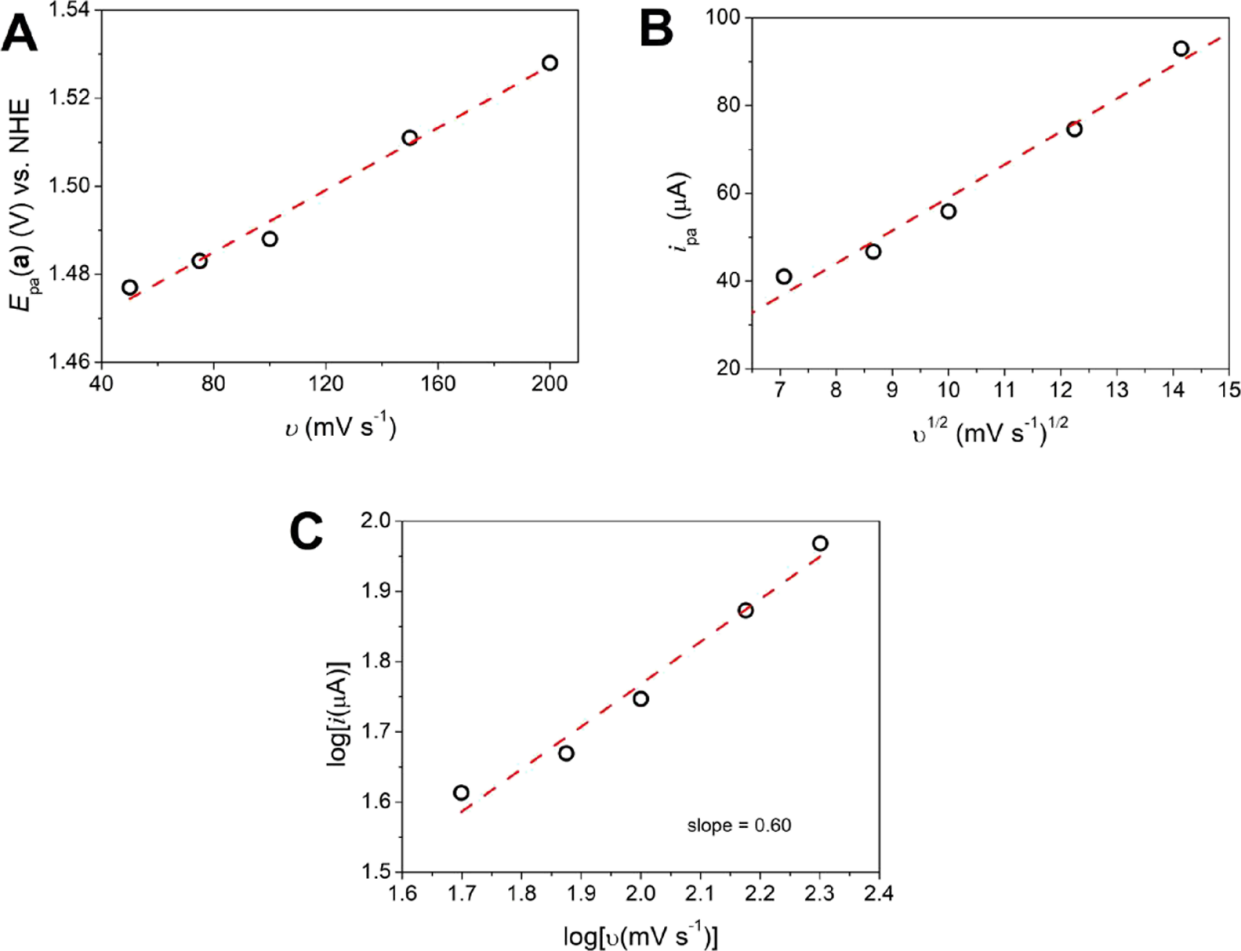
Electrochemical data for process **a** of {[CuPEP]­ClO_4_}_*n*_ in CH_3_CN. (A) Plot of *E*_pa_ versus *v*. (B) Randles–Sevcik plot of *i*_pa_ versus *v* ^1/2^. Electrode area = 0.0314 cm^2^, *C* ^0^ = 1 × 10^–3^ mol dm^–3^, *n* = 1. The diffusion constant was calculated from the slope of the plot *i*_pa_ (A) vs *v* ^1/2^ (V s^–1^): slope = 2.38 × 10^–4^ V^1/2^ s^–1/2^, *D* = 7.96 × 10^–4^ cm^2^ s^–1^. (C) Plot of log­(*i*_pa_) versus log­(*v*).

For process **b**, the anodic and cathodic peaks were observed, and they are assigned to the Cu^I^/Cu^II^ redox couple, which occurs at a more negative potential than process **a** (Figure 5,gray line). *E*_1/2_(b) is independent of the scan rate, with *E*_1/2_(b) = −0.456 V vs NHE at 100 mV s^–1^ (Figure 7A, Figure S15, Table S7). The observed Δ*E* = 110 mV is very close to that of the reversible Fc/Fc^+^ redox couple. The Randles–Sevcik plot shows that the relation *i*_p_ vs *v* ^1/2^ is not linear (Figure 7B), which indicates that the redox species are not freely diffusing in the solution but adsorb at the electrode surface and give a typical linear plot of *i*_p_ vs *v* (Figure 7C).^44^ The ratio *i*_pc_/*i*_pa_ (reverse/forward) increases with scan rates (Figure 7D); this behavior is typical of a chemical reversible reaction coupled to a reversible electron transfer, C_r_E_r_ (eqs S1 and S3).^45,46^ The Cu^I^ species first reacts, probably suffers a geometrical or coordination transformation, and then is subsequently oxidized to Cu^II^. A similar behavior was described for the Cu^II^ complex with the *N*,*N*′-bis­(3-aminopropyl)­oxamido ligand (oxpn) Cu­(oxpn)­Cu­(OH)_2_, which shows the Cu^I^/Cu^II^ redox couple at −0.04 V vs NHE.^47^ When the scan goes up to 2.3 V vs NHE (Figure 5, black line; Figure S14), it is possible to observe a second wave for process **b** at higher scan rates. This is probably due to a Cu^I^/Cu^II^ redox process of a second species that presented a slower redox process and is only observed at higher scan rates behaving as a quasi-reversible process.

**Figure 7:**
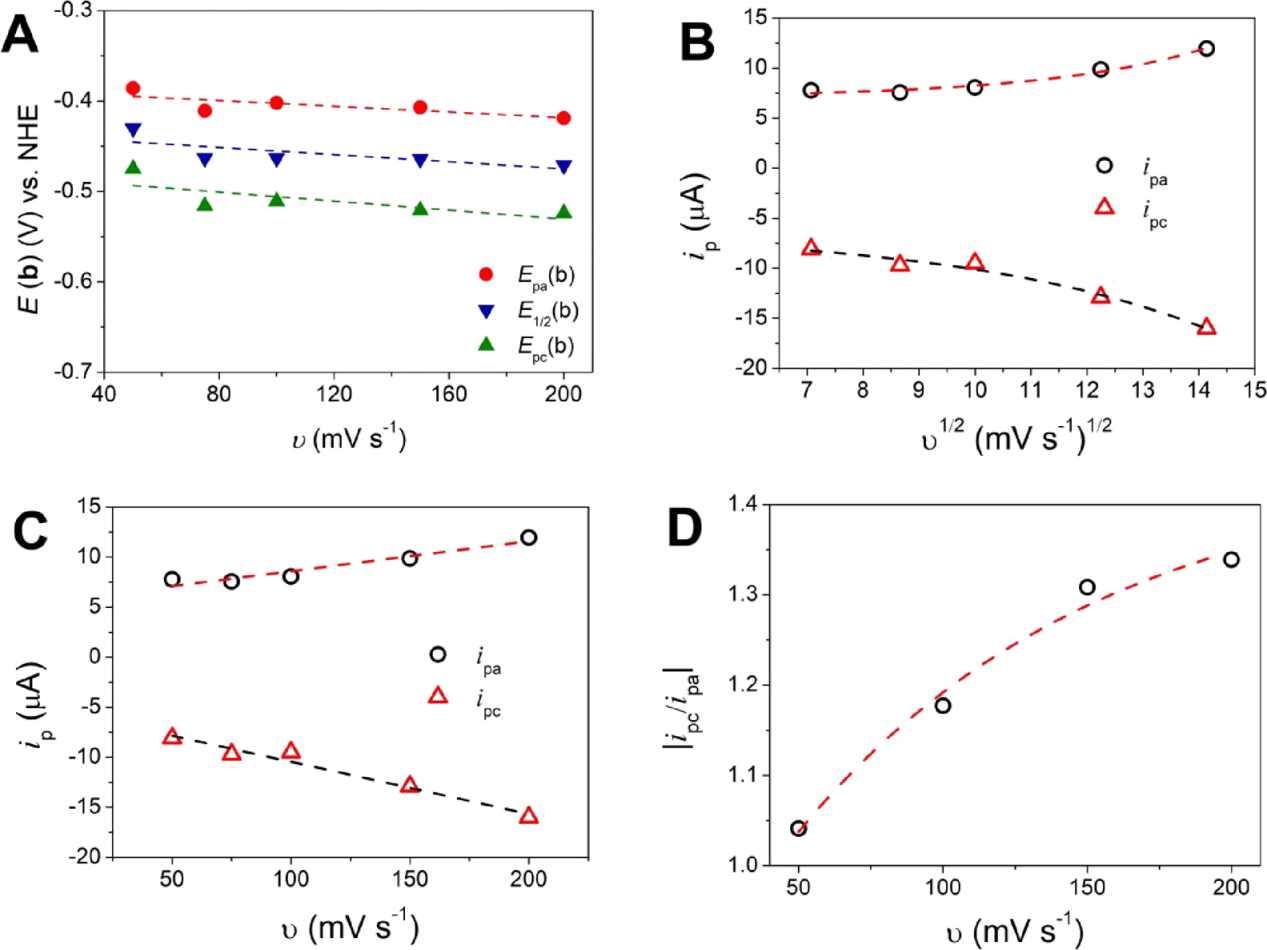
Electrochemical data for process **b** of {[CuPEP]­ClO_4_}_*n*_ in CH_3_CN. (A) Plot of *E* versus *v*. (B) Randles–Sevcik plot of |*i*_p_| versus *v* ^1/2^. (C) Plot of |*i*_p_| versus *v*. (D) Plot of |*i*_pc_/*i*_pa_| versus *v*.

Cyclic voltammograms of {[CuPEP]­ClO_4_}_*n*_ were also acquired in phosphate buffer at pH 12.5 (Figure 5) to investigate its electrochemical behavior in the electrolyte used for the OER tests in the potential window from −0.6 to +1.2 V vs NHE. Process **c** shows *E*_1/2_ = −0.323 V typical of Cu^I^/Cu^II^, and the Δ*E*_p_ of 75 mV represents a reversible system (Figure S16, Table S8). Process **c** is 0.133 V more positive in buffer than **b** in CH_3_CN. The anodic shift of *E*_1/2_ can be associated with the higher Gutmann’s acceptor number of H_2_O in relation to CH_3_CN,^40^ which results in a partial withdrawal of electron density from the ligand by the solvent and increase of the Lewis acidity of the Cu^II^ center.^48^

*E*_pa_(c) and *E*_pc_(c) did not change with the scan rate (Figure 8A), typical of a reversible behavior. The *i*_pa_ and *i*_pc_ values vary linearly with *v* ^1/2^ (Figure 8B), and the slope of log­(*i*) vs log­(*v*) is lower than 1 (Figure S17), indicating a diffusion-controlled process and hence that a homogeneous species is present in solution. The |*i*_pc_/*i*_pa_| ratio stays close to 1 at all scan rates (Figure 8C). The diffusion constant is 1.49 × 10^–5^ cm^2^ s^–1^ for *i*_pa_ and 1.02 × 10^–5^ cm^2^ s^–1^ for *i*_pc_ (Table S9). Table S10 shows the *E*_1/2_ reported for similar copper­(II) compounds attributed to the Cu^I^/Cu^II^ process and matches our findings for process **c**. Processes **b** and **c** are probably related to the similar Cu^I^/Cu^II^ process; however, they are quasi-reversible in CH_3_CN and reversible in buffer, indicating a faster kinetics in the later.

**Figure 8:**
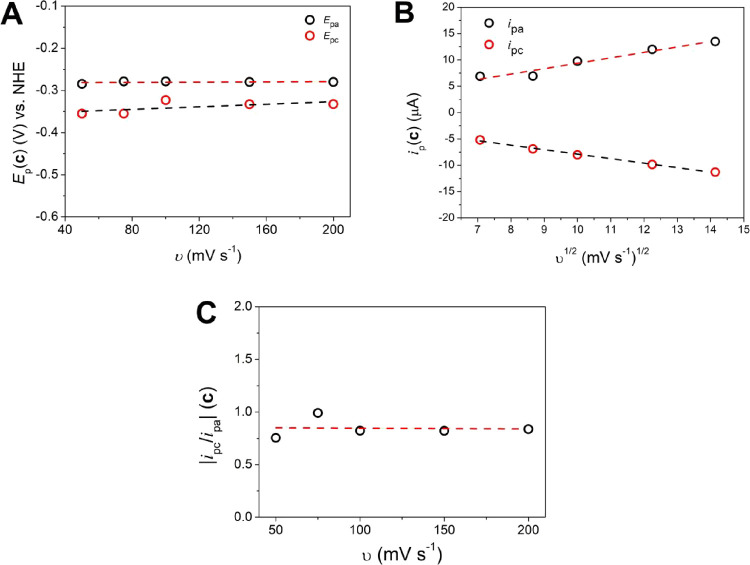
Electrochemical data for process **c** of {[CuPEP]­ClO_4_}_*n*_ in buffer at pH 12.5. (A) Plot of *E*_pa_ and *E*_pc_ versus *v*. (B) Randles–Sevcik plot of *i*_pa_ versus *v* ^1/2^. (C) Plot of *i*_pa_/*i*_pc_ versus *v* ^1/2^.

### 3.3 Oxygen Evolution Reaction (OER)

Electrocatalytic OER tests were carried out by cyclic voltammetry in phosphate buffer at pH 12.5 using FTO as the working electrode and {[CuPEP]­ClO_4_}_*n*_ as the electrocatalyst. Initially, only the OER was investigated in the potential window from 0.4 to 1.4 V vs NHE at 100 mV s^–1^. Compared with the bare FTO electrode, a higher current density was observed for {[CuPEP]­ClO_4_}_*n*_ (Figure 9A). The onset overpotential measured at *j* = 0.1 mA cm^–2^ is η = 394 mV (Table S11), while the overpotential is η = 543 mV at *j* = 1 mA cm^–2^ and η = 721 mV at *j* = 4 mA cm^–2^. Comparing with copper­(II) compounds from the literature summarized in Table S12, {[CuPEP]­ClO_4_}_*n*_ presents lower overpotentials in all current densities

**Figure 9:**
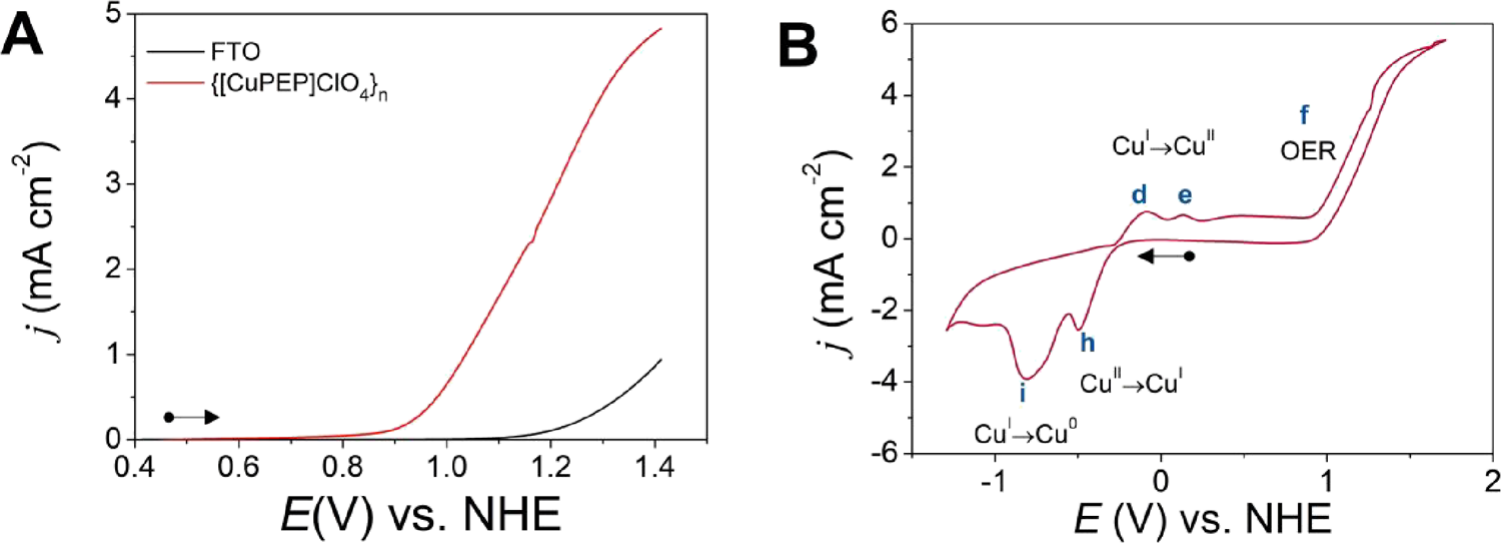
Electrocatalytic OER activity catalyzed by {[CuPEP]­ClO_4_}_*n*_ at 5 × 10^–3^ mol L^–1^ using FTO as WE in 0.1 mol L^–1^ in phosphate buffer at pH 12.5 at 100 mV s^–1^. (A) Cyclic voltammetry (forward scan) was performed from 0.4 to 1.4 V vs NHE. (B) Cyclic voltammogram from −1.3 to 1.7 V vs NHE.

Cyclic voltammetry was also carried out in the potential window from −1.3 to 1.7 V vs NHE, and the corresponding overpotentials are shown in Table S14, where the OER and the redox processes of {[CuPEP]­ClO_4_}_*n*_ were observed simultaneously to evaluate the OER kinetics (Figure 9B). Differently from the CV in the range of 0.4 to 1.4 V vs NHE discussed above (Figure 9A), more oxidation and reduction peaks appeared. Six redox peaks could be identified. The anodic peaks **d** and **e** were attributed to Cu^I^/Cu^II^, and **f** is due to OER catalytic current. The cathodic peaks **h** and **i** can be ascribed to Cu^II^/Cu^I^ and Cu^I^/Cu^0^, respectively, which were also described previously.^7^

The CV was carried out in different potential windows to elucidate the origin of the higher current associated to the peaks **h** and **i** (Figure S18). At the lowest potential range, a pair of anodic/cathodic waves of *E*_1/2_ around 0 V vs NHE with a very low relative current were observed. The Δ*E* of 300 mV is characteristic of a quasi-reversible redox process. As the potential window becomes larger, new peaks appeared and the current rose. When the potential is scanned above 0.8 V vs NHE, the OER starts and a current crossover is observed on the reverse scan, which can be associated with heterogeneous film formation at the surface of the FTO.^7^ According to the literature, the peaks formed from the copper electrode in the alkaline electrolyte in the range between OER and HER are characteristic of copper oxides and hydroxides,^49^ which agree with the voltametric profiles described for {[CuPEP]­ClO_4_}_*n*_ and confirm the formation of heterogeneous CuO_*x*_-OH at the FTO surface.

Cyclic voltammograms were recorded in dependence of the scan rates (Figure S19). The normalized peak current by the square root of the scan rate (*i*/*v* ^1/2^) increases as the *v* decreases only for the OER catalytic peak **f** (Figure S19), indicating that a catalytic process contributed to the oxidation peak because a chemical step and rate-limiting O–O bond formation are taking place before the electron transfer at the electrode.^10^ The *E*_p_ of processes **d**, **e**, **h**, and **i** varies with the scan rate, typical of an irreversible process (Figure 10A); the respective diffusive peak current *i*_d_ varies linearly with *v* ^1/2^ (Figure 10B); and the slope of log­(*i*) vs log­(*v*) is lower than 1 (Figure S20), indicating a diffusion-controlled process.^50^ Diffusion constants are in the range of 3.1 × 10^–6^ to 6.8 × 10^–5^ cm^2^ s^–1^ (Table S15).

**Figure 10:**
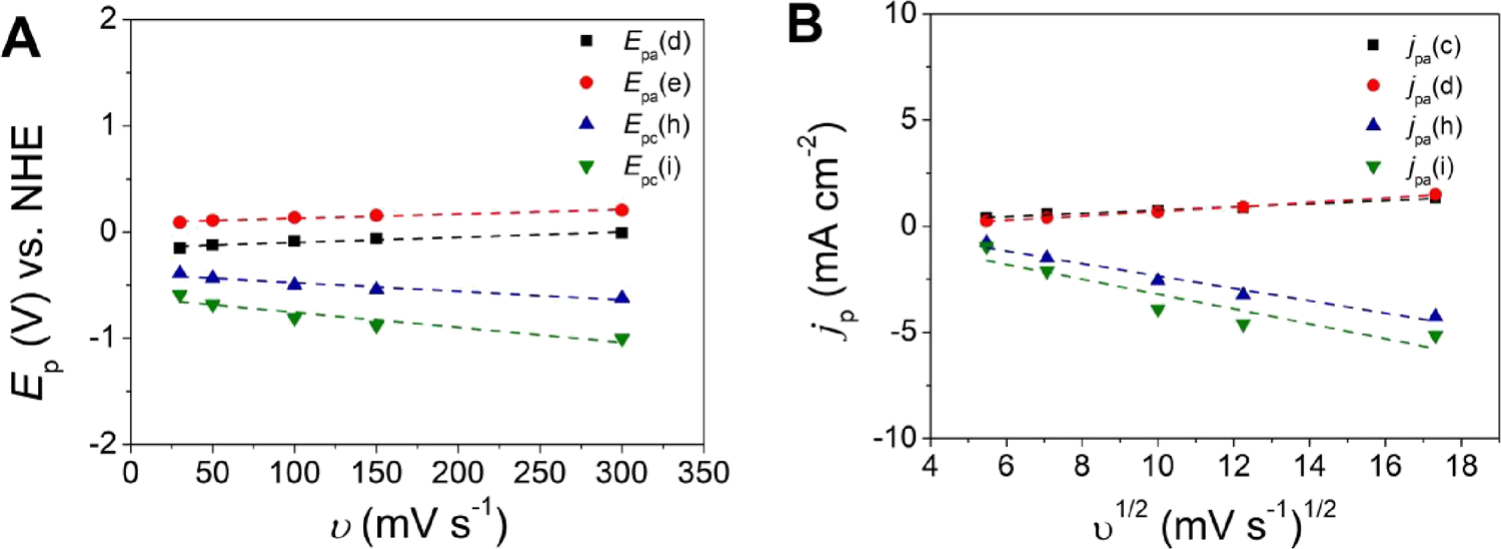
Electrochemical data for processes **d, e, h**, and **i** of {[CuPEP]­ClO_4_}_*n*_ in buffer at pH 12.5 at FTO. (A) Plot of *E*_p_ versus *v*. (B) Randles–Sevcik plot of *i*_pa_ versus *v* ^1/2^.

The OER was measured at different concentrations of catalyst, and the plot of log­[*j*] versus log­[concentration] indicates a first-order catalytic reaction, as shown in Figure 11A. The catalytic peak current *i*_cat_ in CV can be described by the model E_r_C_cat_ (eqs S1 and S4), a reversible electrochemical reaction coupled to a catalytic reaction, as shown in eq S6.^8^

**Figure 11:**
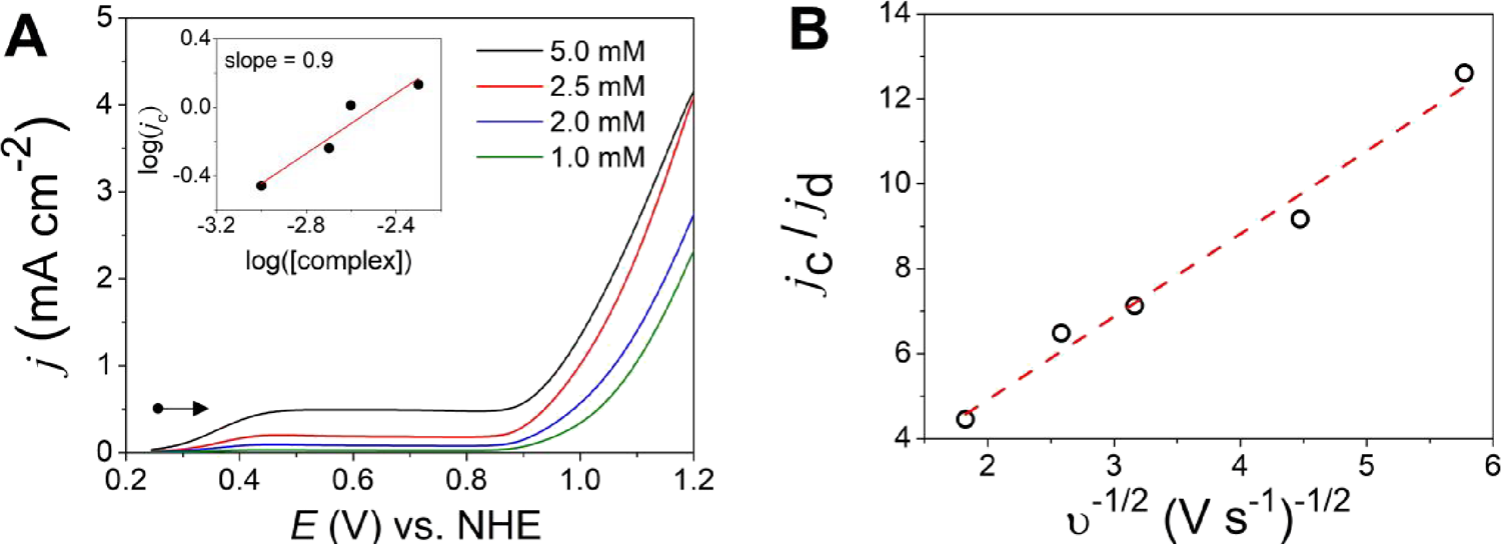
Electrocatalytic OER activity was catalyzed by {[CuPEP]­ClO_4_}_*n*_ using FTO as WE in 0.1 mol L^–1^ in phosphate buffer at pH 12.5 at 100 mV s^–1^. (A) Cyclic voltammetry (forward scan) at different catalyst concentrations (inset: plot of log­(*j*) versus log­[{[CuPEP]­ClO_4_}_*n*_]). (B) Plot of *j*_c_/*j*_d_ versus *v* ^–1/2^. *j*_c_ was taken from the OER catalytic process **f** at *E* = 1.6 V, and *j*_d_ was taken at the peak potential from process **d** (Figure S19). Slope = 2.019 and *k*_cat_ = 2.1 s^–1^, assuming *n* = 4 e^–^.

The apparent first-order rate constant of the catalytic OER, *k*_cat_, is also referred to as the turnover frequency (TOF) of the catalyst and was calculated by eq S7, where *i*_d_ is the plateau current density of the noncatalytic wave Cu^I^/Cu^II^ (process **d**) and *v* is the scan rate in V s^–1^.^8^

The plot *i*_cat_/*i*_d_ versus *v* ^–1/2^ is linear and matches with the trend from a pure diffusive behavior region to a pure kinetic behavior region as the scan rates decrease (Figure 11B).^8^ From the slope, *k*_cat_ was calculated to be 2.1 s^–1^. Considering the simplified model E_r_C_cat_, the calculated *k*_cat_ can serve only as an estimate of the complex four-electron OER catalytic rate.^8^ The *k*_cat_ reported for [(TGG^4–^)­Cu^II^-OH_2_]^2–^ was 33 s^–1^ (TGG = triglycylglycine) at pH 11.^10^ The *k*_cat_ reported for Cu-L (H_2_L = 6,6′-dihydroxy-2,2′-bipyridine) was 0.44 s^–1^ at pH 12.4,^8^ showing the high reaction rate executed by {[CuPEP]­ClO_4_}_n_.

Stability tests of {[CuPEP]­ClO_4_}_*n*_ in the OER electrocatalytic conditions were carried out to investigate whether the catalysis proceeded via homogeneous or heterogeneous catalysis. Some studies have reported that copper­(II) molecular catalysts suffered decomposition at the WE surface, forming heterogeneous metal oxide/hydroxide films, which are the real OER catalytic species.^7,51–53^ First, 100 cycles of CV were performed in a wide potential window of −1.3 to +1.7 V vs NHE as shown in Figure 12A, and a small increase in the current density of the peaks in the region of −1.3 to +1 V vs NHE was observed. A current crossover in the CV reverse scan, highlighted in Figure S21, is indicative of film formation in the WE surface,^7^ which is also evidenced by the brown film observed in the FTO (Figure S21). The structure of the ligand possesses groups that are susceptible to oxidative deamination and cleavage under the strong alkaline and oxidative conditions of the OER,^7^ which upon release of the Cu^II^ ions generate the heterogeneous CuO_*x*_-OH OER catalyst.^52^ The electrode was rinsed after the OER and tested again in pure electrolyte without {[CuPEP]­ClO_4_}_*n*_ (Figure 12B), and the current density is higher than that of the pure FTO, indicating that the film is active for the OER. The UV–vis spectrum after OER showed that a decrease of 15% in the absorbance accounted for the film deposition. To further investigate the stability of {[CuPEP]­ClO_4_}_*n*_ for a higher number of cycles, 1000 cycles of CV were carried out as shown in Figure S24. A similar behavior was observed in comparison with the test of 100 cycles; however, after the 200th cycle, a decrease in the current was observed, as shown in Figure S24A, inset, which can be associated with the higher thickness of the film and higher resistance to the charge transfer process of the FTO/film/electrolyte interfaces. The UV–vis of the electrolyte solution containing the complex shows a lower absorbance, indicating more consumption of the complex and the formation of a thicker film.

**Figure 12:**
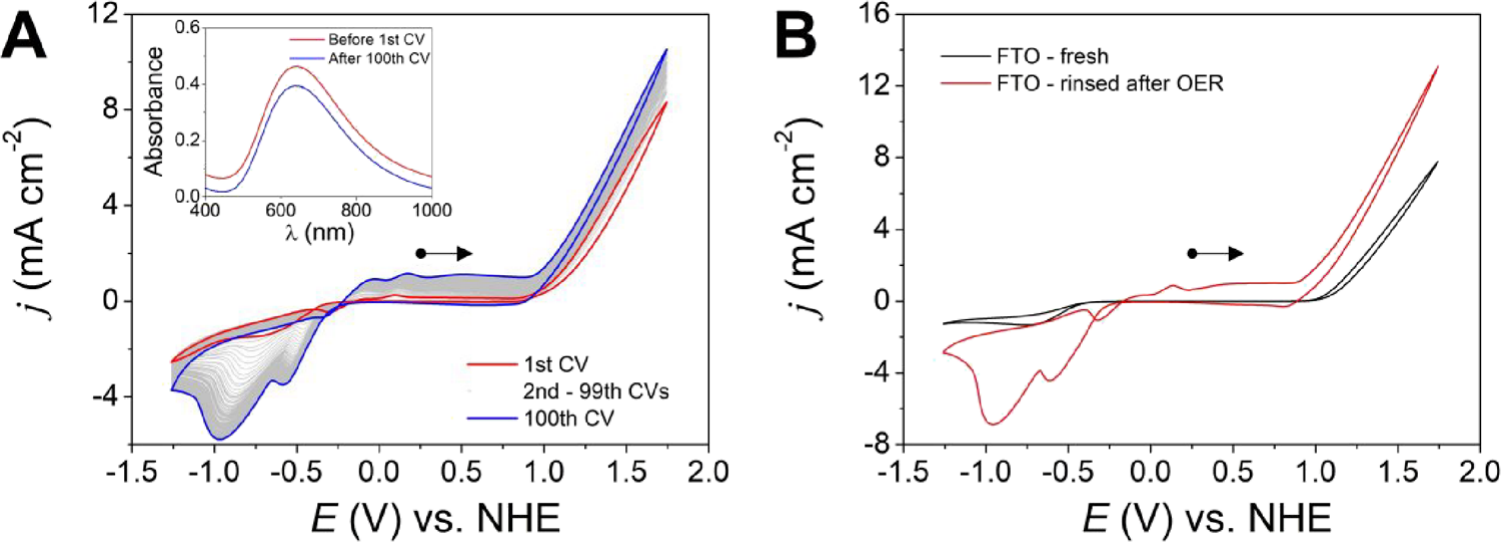
Cyclic voltammetry in 0.1 mol L^–1^ phosphate buffer at pH 12.5 and 100 mV s^–1^ at a wide potential window. (A) One hundred cycles of CV of OER activity catalyzed by 5.0 × 10^–3^ mol L^–1^ {[CuPEP]­ClO_4_}_*n*_ and FTO WE; inset: UV–vis of the electrolyte solution after and before 100 CV cycles. (B) OER test was performed in the pure electrolyte using the rinsed FTO WE after 100 CV cycles.

The contribution of homogeneous and heterogeneous OER electrocatalysis from both {[CuPEP]­ClO_4_}_*n*_ and the formed film was estimated taking into account the current density at 1.3 V versus NHE of the first cycle as 100% of homogeneous contribution. After the 100th cycle, the homogeneous catalysis contribution would also suffer a decrease of 15% in current density, from 3.2 to 2.7 mA cm^–2^. Therefore, the current density of 3.94 mA cm^–2^ in the 100th cycle would be composed of 69% and 35% homogeneous and heterogeneous catalysis, respectively. However, when the film was washed and immersed in pure electrolyte, as shown in Figure 12B, the current density was 5.71 mA cm^–2^, higher than in the solution with {[CuPEP]­ClO_4_}_n_ at the 100th cycle, which indicates that the heterogeneous activity of the film is higher in the pure electrolyte. This can be attributed to the fact that the current density applied at the WE was partially spent to evolve oxygen and also to produce the film, but in the pure electrolyte, the current is only directed to OER.

A similar behavior was observed when the stability test was carried out by CV in a narrower potential window of +0.4 to +1.4 V vs NHE, as shown in Figure S22. Copper­(II) salts are known to form CuO_*x*_-OH film at the electrode in alkaline pH and play a role in the OER electrocatalysis.^54^

For comparison, 100 cycles of CV using Cu­(ClO_4_)_2_ instead of {[CuPEP]­ClO_4_}_*n*_ were carried out, and negligible film formation was observed (Figure S23), probably because of the precipitation of Cu­(OH)_2_ at pH 12.5 and lower accessibility to the FTO WE, which is not the case for {[CuPEP]­ClO_4_}_*n*_ since it is highly soluble in water and readily accessible to the WE.

Chronopotentiometry of {[CuPEP]­ClO_4_}_*n*_ at 5 mA cm^–2^ is shown in Figure 13A. The overpotential started at 1088 mV, but after 45 min, it decreased to 735 mV and stayed at this value up to 3 h. This increase in activity is assigned to the formation of a film. Figure 13A, inset, shows the UV–vis spectra before and after 3 h of electrolysis and the decreased concentration of {[CuPEP]­ClO_4_}_*n*_ of 19% in the electrolyte. CV confirmed the decrease in the catalyst concentration by the lower current associated with the copper redox processes, as well as the lower OER current due to homogeneous catalysis, tested by a fresh FTO in the remaining catalyst electrolyte (Figure S25). Figure 13B and Figure S26 show the LSV of the FTO WE after the rinsing test in a pure electrolyte and shows a higher OER activity than the pure FTO, proving the heterogeneous catalytic effect exhibited by the film. The dissolved oxygen was measured for the first 3 h, and 38% Faradaic efficiency was achieved, as shown in Figure S27. It is possible to observe that in the first 30 min, the Faradaic efficiency was 100% when only the homogeneous catalysis was taking place. After 30 min, part of the current was consumed by the deposition of the film, and the Faradaic efficiency decreased. After 3 h of chronopotentiometry, the overpotential rose slowly until a sudden increase to 2170 mV at around 16 h caused by the film detachment from the FTO (Figure S28A). An even lower concentration of the catalyst in the electrolyte was observed by CV (Figure S25) and UV–vis (Figure 28B) after 16 h, indicating that after 3 h, the film was still growing but the higher thickness increased the resistance of charge transfer until complete detachment. A similar behavior was described for a Robson-type macrocyclic dinuclear copper complex that also had been electrodeposited over a FTO substrate and suffered a sudden decrease of current at 80 min during a chronoamperometry experiment at −1.20 V vs Ag/AgCl.^53^

**Figure 13:**
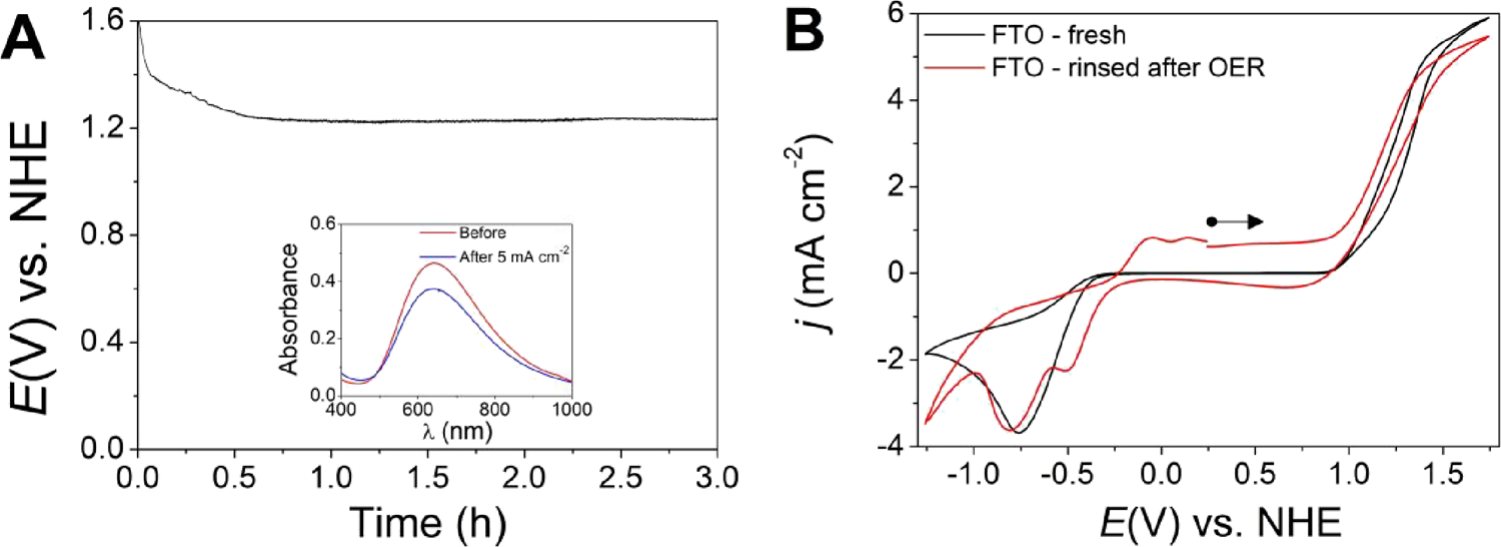
(A) Chronopotentiometry at 5 mA cm^–2^ for 3 h catalyzed by 5.0 mol L^–1^ {[CuPEP]­ClO_4_}_*n*_, FTO WE, and 0.1 mol L^–1^ phosphate buffer at pH 12.5; inset: UV–vis of the electrolyte solution after and before the chronopotentiometry. (B) CV at 100 mV s^–1^ of the OER test in the pure electrolyte using the rinsed FTO WE after the chronopotentiometry.

AAS analysis of the film after 3 h of chronopotentiometry at 5 mA cm^–2^ shows 0.264 mg of deposited Cu in FTO. Furthermore, the ECSA of the film was 101.9 cm^2^, calculated from the doble-layer capacitance (*C*_dl_) measured by the CVs in the non-Faradaic region, as shown in Figure S29 and described in Section S4.4. From these parameters, the mass activity of 15.6 A g^–1^ was calculated in relation to the mass of Cu deposited on the film. A specific activity of 43 μA cm^–2^ was obtained. The Tafel slope of the films was calculated as 448 mV dec^–1^ for the film after 100 CVs (Figure S22D) and 278 mV dec^–1^ for the film after chronopotentiometry (Figure S26B), showing superior kinetics for the OER for the latter. Comparison with the literature shows Tafel slopes in the range of other heterogeneous copper catalysts (Table S13).

The films deposited over FTO were characterized after stability tests. XRD analysis of the film formed after 100 cycles of CV showed the formation of peaks related to Cu­(OH)_2_ (JCPDS no. 13-420),^55^ Cu_3_(PO_4_)_2_,^8^ and other unidentified peaks that could be related to copper oxides, hydroxides, or phosphates (Figure 14A), as described previously for the copper­(II) complex.^7^ The diffractogram after 3 h of chronopotentiometry at 5 mA cm^–2^ shows peaks attributed to CuO. The diffractogram of the film after 1000 cycles of CV shown in Figure S24C show the formation of Cu(0) evidenced by the planes (111) and (200) (JCPDS no. 003-1018), as also evidenced by the red metallic color of the film. Figure 14B shows the Raman spectra, and it is evident that the film after chronopotentiometry presents a purer and more homogeneous phase than the film after 100 CVs. The band at 291 cm^–1^ is assigned to Cu­(OH)_2_, that at 310 cm^–1^ to copper phosphate, that at 582 cm^–1^ to CuO, and that at 610 cm^–1^ can be associated to CuO or Cu^III^ oxides.^56^ Figure S30 compares the Raman spectra for the films, where the band at 1375 cm^–1^ corresponds to the pure FTO and the decrease of the band by the coverage of the film. The film after 1000 CVs showed a different profile, where the FTO was also fully covered and the bands related to CuO_*x*_ and Cu­(OH)_2_ are of very low intensity, corroborating the major formation of Cu(0).

**Figure 14:**
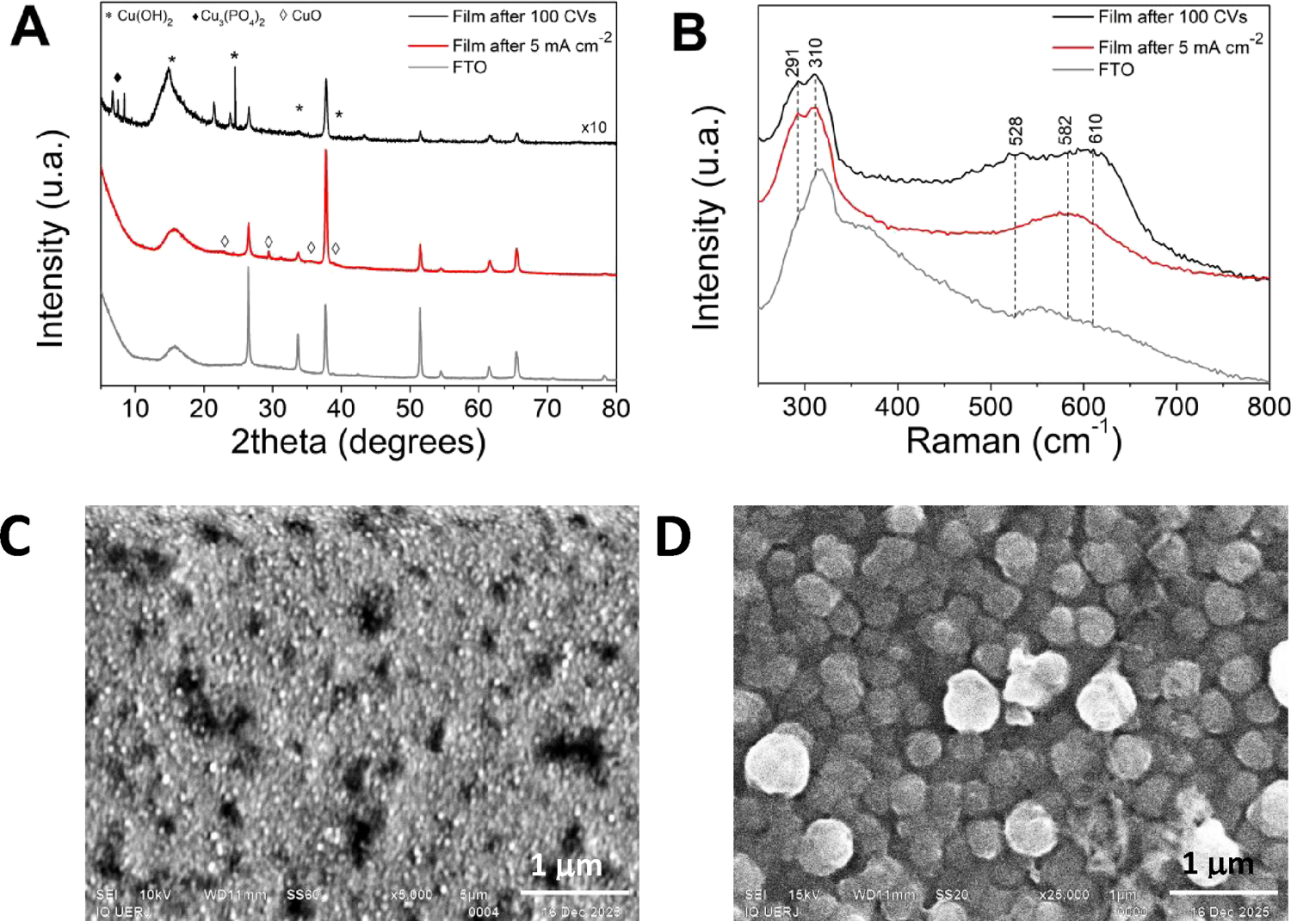
(A) XR diffractograms; (B) Raman spectra; and (C: CV and D: chrono) SEM of the FTO films obtained after 100 cycles of cyclic voltammetry and chronopotentiometry at 5 mA cm^–2^ for 2 h 10 min of OER activity catalyzed by 5.0 mM {[CuPEP]­ClO_4_}_*n*_ and 0.1 mol L^–1^ phosphate buffer at pH 12.5.

SEM and SEM-EDS analyses of the films after 100 CV cycles (Figure 14C and Figure S31) show the formation of a homogeneous, porous, amorphous film majorly composed of Cu and with minor quantities of O and P, in agreement with the phases Cu­(OH)_2_ and Cu_3_(PO_4_)_2_ shown in XRD. Similar morphology and elemental composition are observed after the chronopotentiometry for 1.5 h at 5 mA cm^–2^ (Figure S32); however, after a longer period of 2.2 h, a change in morphology was observed with the formation of spheres around 500 nm, homogeneously distributed (Figure 14D andFigure S33), majorly composed of Cu and O, and with minor quantities of P, in agreement with the CuO phase shown in XRD. Nitrogen was not detected, indicating no remnant of the ligand. After 16 h of chrono, the film completely detached from the FTO substrate, leaving no trace of copper compounds (Figure S34) and assuming the same morphology and surface composition of the pristine FTO with the major elements Sn and O (Figure S36).

Electronic and chemical modifications in the samples after stability tests were revealed by XPS measurements, as shown in Figure 15, after 100 CV cycles (top panels) and after 3 h of chronopotentiometry at 5 mA cm^–2^ (bottom panels). The Cu 2p_3/2_ line reveals that both samples are predominantly composed of Cu­(II) species, as depicted in Figure 15a,c. In addition to the remarkable high binding energy peak around 934–935 eV, indicated as C2 in each figure, one can observe a well-defined satellite contribution in the 941–944 eV region. This satellite arises due to shakeup effects related to modification in the Cu electronic structure distribution, ultimately related to the Cu­(II) chemical state.^57^ Despite the similarity of the satellite region, subtle but reproducible differences in the shape are observed between the two electrochemical scenarios. Modifications in the shape of satellite region are commonly associated with changes in surface homogeneity and local coordination environment, in addition to reflecting differences between oxide- and hydroxide-dominated Cu­(II) phases.^57,58^ For instance, while CuO-like species often display a well-separated shoulder, Cu­(OH)_2_-like structures yield broader, more convoluted satellite envelopes.^58,59^ In the sample after 100 CVs, the Cu 2p_3/2_ line requires two main components for an adequate description, as shown in Figure 15a. The labels C2 and C1 stand for a dominant component centered at 935.9 ± 0.6 eV (C2) and a weaker contribution at 932.03 ± 0.5 eV, respectively. While the former is addressed to Cu­(II) species, such as CuO/Cu­(OH)_2_ and Cu_3_(PO_4_)_2_ species, the latter is often attributed to reduced Cu environments.^57^ The coexistence of multiple chemical states suggests a chemically heterogeneous surface, consistent with the dynamic redox conditions imposed by cyclic voltammetry. In contrast, in the sample after chrono, the Cu 2p_3/2_ envelope is well described by a single Cu­(II) component (C2), as depicted in Figure 15c. This component arises centered at 934.28 ± 0.6 eV. This observation suggests that prolonged operation under constant anodic bias promotes the formation of a more uniform Cu­(II) layer, suppressing reduced copper domains (like Cu_2_O). These differences indicated that the electronic structure of the films is sensitively dependent on the electrochemical protocol employed during the OER operation. It is worth noting that absolute binding energy values can vary significantly depending on several factors, especially the calibration procedure. In the literature, for instance, the main Cu­(II) peak at full CuO samples has been reported at values ranging from 933.76 to 935.16 eV.^58,60^ Such variations might lead to ambiguous assignments if the absolute binding energies alone are considered. On the other hand, Cu 2p and satellite regions’ peak shape, and notably relative binding energy, might be a reliable criterion for correctly identifying chemical states. The shape of the satellite region of both samples is not fully consistent with either a CuO or Cu­(OH)_2_ structure. Although the shakeup presented in both spectra arises from Cu­(II) species, the coexistence of both structures cannot be ruled out. This aspect is emphasized when computing the C* parameter, defined as the ratio of the Cu­(II) main peak area (C2) to the satellite area. Usually, for fully individual CuO or Cu­(OH)_2_ structures, the C* parameter spans from 1.57 to 1.89, respectively.^58,60^ In our case, this parameter is 1.1, which is quite lower than the results previously reported. This finding might be explained due to the coexistence of both structures, increasing the satellite area and then decreasing the C* parameter.

**Figure 15:**
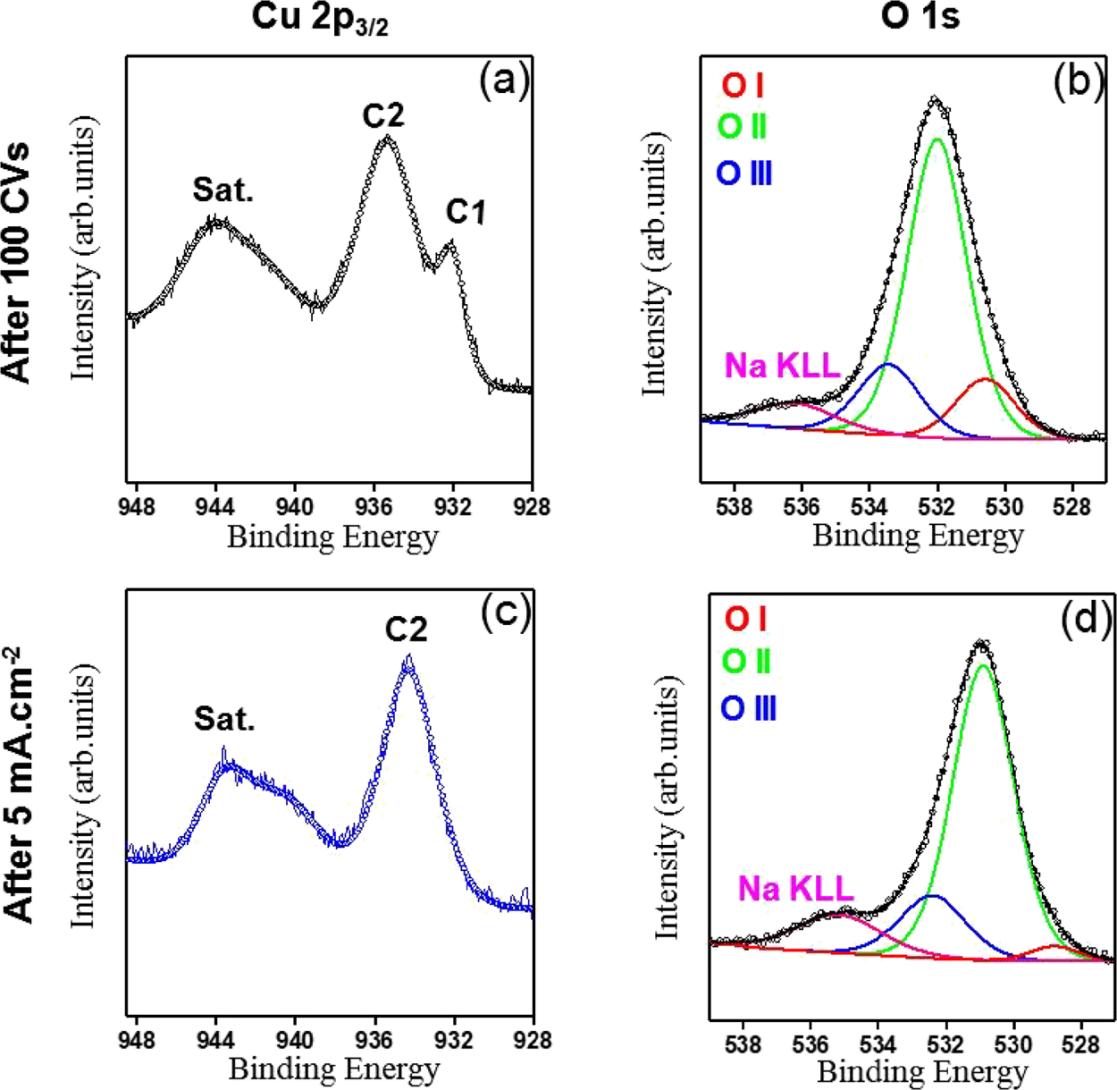
XPS analysis of the films after the OER stability tests. Top panels correspond to the sample after 100 CVs. Bottom panels correspond to the sample after chronopotentiometry at 5 mA cm^–2^ for 3 h. High-resolution spectra of the Cu 2p_3/2_ region are shown in panels a and c, and the spectra of the O 1s region are shown in panels b and d.

Further insights into the surface chemistry of the films are obtained from the spectral data of the O 1s region depicted in Figure 15b,d. In both scenarios, the O 1s envelope is dominated by a component assigned to hydroxyl groups (O II), indicating extensive surface hydroxylation after OER. A low binding energy component (O I) around 530.5 ± 0.7 eV is also observed and is commonly attributed to lattice oxygen contributions. The high binding energy component (O III) arises from oxygen in dative bonding configurations, such as carbonyl/carbonate-like, or copper phosphate species derived from surface residues, electrolyte exposure,^60,61^ and/or film growth environment. The additional high binding energy component around 535.5 ± 0.6 eV is attributed to the Na KLL auger line, consistent with the presence of Na species identified in the survey spectra of each sample (see Figure S36).

Notably, the relative intensity of the lattice oxygen component is significantly reduced in the film after chrono compared with the film after CV. This finding corroborates the interpretation derived from the Cu 2p analysis. While the surface after CV cycling comprises a mixed environment in which Cu­(II) and reduced copper species coexist, the constant applied current favors a more homogeneous Cu­(II) surface. Conversely to absolute binding energy analysis, relative binding energies allow us to make some speculations. For instance, although the presence of hydroxyl groups reported in the O 1s spectra might suggest the formation of Cu­(OH)_2_, the separation Δ*E*(Cu 2p – Cu LMM) is 16.57 eV, which agrees very well with the separation in CuO structures.^58^ Indeed, when CuO species are dominant, the relative binding energies are slightly lower than those of hydroxide or other Cu­(II) structures. The apparent shift to low binding energy of sample B with respect to sample A, suggested in Figure 15, might also indicate the prevalence of CuO after CP cycles. In this scenario, we might speculate that CuO and Cu­(OH)_2_ coexist, with hydroxide shaping the satellite envelope and oxide domains contributing to more homogeneous, conductive media after CP. Differences in the shape of the VB, indicated by the black and red arrow in Figure S37, further support that the electrochemical protocol plays a crucial role on the chemical composition of the catalyst surface as well as modulates its electronic structure. It is worth noting that although CP treatment initially produces a highly homogeneous surface, excessively long electrochemical operation may destabilize the film, ultimately leading to a vanishing Cu 2p signal, as illustrated in Figure S38 for the film after chronopotentiometry at 5 mA cm^–2^ for 12 h.

## Conclusions

A new water-soluble copper­(II) coordination polymer is described with a four-dentate nitrogenated and carboxylate derivative ligand, which presents a one-dimensional chain revealed by an X-ray single crystal. The coordination polymer was thoroughly characterized structurally and electrochemically. The coordination polymer was tested as an electrocatalyst in the oxygen evolution reaction at buffer pH 12.5, and an onset overpotential of 394 mV was delivered at 0.1 mA cm^–2^, comparable to copper­(II) catalysts reported to date. The first-order rate constant of 2.1 s^–1^ revealed favored kinetics for the OER and promising molecular catalytic activity. Stability tests of the catalyst at OER conditions revealed the formation of a film at the FTO surface composed of copper oxides, hydroxides, or phosphates, resulting from the coordination polymer decomposition. The film was active in OER by a heterogeneous pathway; however, an optimum growth time was observed, after which film detachment was observed. Furthermore, the film reached higher current densities in the OER than the homogeneous coordination polymer electrolyte, indicating that although the homogeneous catalysis is taking place, the coordination polymer can be considered as a precursor for a more active heterogeneous catalyst.

## Supplementary Material



## Data Availability

The data underlying this study are available in the published article and its Supporting Information.

## Notes

^1^H and ^13^C­{^1^H} NMR and FTIR spectra of the ligand and precursors; single-crystal structure, FTIR, UV–vis, ESI-MS, and CV data of the coordination polymer; OER electrocatalysis and stability data; and XRD, Raman, SEM-EDS, ECSA, and XPS data of the film after the stability test (PDF)
