# Supplementary material for: A New Water-Soluble Copper(II) Coordination Polymer for Electrocatalytic Oxygen Evolution Reaction
Source: ACS Org Inorg Au. 2026 Jan 27;6(2):209–24. doi: 10.1021/acsorginorgau.5c00077 (PMC13047454; doi:10.1021/acsorginorgau.5c00077)
Supplement: Supplementary file 1 [file gg5c00077_si_001.pdf]

Supporting Information

A new water-soluble copper(II) coordination polymer for electrocatalytic oxygen evolution reaction

Renata P. P. Macedo,[a] Joice L. Carvalho,[a] P.R.A de Oliveira,[b] Lorenzo C. Visentin,[c] Nakédia M. F. Carvalho[a,*]

a Universidade do Estado do Rio de Janeiro, Instituto de Química, Rua São Francisco Xavier, 524, Maracanã, Rio de Janeiro, RJ, 20550-900, Brazil.

b Universidade Federal do Rio de Janeiro, Instituto de Física, Av. Athos da Silveira Ramos, 149, Cidade Universitária, Rio de Janeiro, RJ, 21941-909, Brazil

c Nanobusiness Informação e Inovação Ltda, Avenida das Américas, 2480, Barra da Tijuca, Rio de Janeiro, RJ, 22640-101, Brazil

*nakedia@uerj.br

S1. Materials and measurements

The reagents and solvents were used as received from commercial sources.

S1.1. Characterization techniques

^{1}H NMR and $^{13}C\{^{1}H\}$ NMR spectra were acquired in a Bruker DRX-200 spectrometer and the chemical shifts were referenced to the solvent. Infrared spectra were collected on a FTIR Nicolet Magna-IR 760 spectrophotometer, with the samples dispersed in KBr disks or as a film on NaCl window. Elemental analysis was carried out in a Perkin-Elmer 2400 CHN, with AD-4 Autobalance in tin capsules. UV-Vis spectra were recorded on a Shimadzu 1601PC spectrophotometer in acetonitrile. Conductivity measurements were carried out with a 1×10^{-3} mol dm^{3} solution in acetonitrile using a BioCristal NT CVM conductivimeter, employing a conductivity cell CA150 of 1 cm^{2}. The pH was measured using a Metrohm 827 pH Lab pH meter. Electrospray ionization time-of-flight (ESI-TOF) mass spectrometer was acquired in a micrOTOF instrument, Bruker Daltonics, Bremen, Germany, using methanol/water as solvent, at capillary voltage of 5.5 kV in positive ion mode, and capillary exit of 100 V. Polycrystalline X-ray diffraction (XRD) analysis was performed in a Bruker D8 ADVANCE (Bruker®, Germany) using Cu-Ka radiation (k = 1.5406 Å). Raman spectroscopy was acquired in a confocal Raman microscope XploRA™ PLUS (Horiba, France). Dissolved oxygen was determined using a Hanna moledo HI6421P meter equipped with a HI7631233 polarographic probe. The probe was calibrated using a zero-oxygen standard solution (HI7040-2) provided by the manufacturer. X-ray photoelectron spectroscopy (XPS) experiments were carried out in an ultra-high-vacuum system operating at a base pressure of approximately 8×10^{-10} mbar. The measurements were performed using a PHOIBOS 150 hemispherical electron analyzer (SPECS), providing an overall energy resolution of

about 0.7 eV. Photoemission was excited by a non-monochromated Al Kα X-ray source (hν = 1486.6 eV) operated at 100 W, resulting in a sample current below 1 nA. All spectra were collected at room temperature with a photoelectron take-off angle of 60° to increase surface sensitivity, probing an effective analysis area of approximately 1–2 mm^2. Wide-scan survey spectra and high-resolution core-level spectra were acquired using pass energies of 50 eV and 30 eV, respectively. The spectrometer energy scale was calibrated using the Au $4f_{7/2}$ reference line at 84.0 eV. Charge correction was applied by referencing the C 1s signal to 285.0 eV. Peak fitting and quantitative analysis were performed using CasaXPS software [S1]. A Shirley background was employed, and the photoemission lines were fitted using mixed Gaussian–Lorentzian functions (GL(x)), with x=50 providing the lowest residual across all analyzed regions. Elemental quantification was based on Scofield photoionization cross-sections combined with inelastic mean free path (IMFP) values appropriate for the corresponding kinetic energies and core levels [S2,S3].

S1.2. Single crystal and polycrystalline X-ray diffraction

Single crystal X-ray diffraction data of the coordination polymer $\{[CuPEP]ClO_4\}_n$ was collected using a Bruker KAPPA CCD diffractometer [S4] at 295 K and Mo graphite monochromatic radiation. The cell parameters were obtained from reflections of net planes with random orientation in the Ewald Sphere using the program PHICHI [S5], and were refined using the program DIRAX [S6]. The procedures involved the use of independent reflections for the determination of the space group, solution and refinement. Data reduction was achieved using the program EvalCCD [S7]. Intensities were corrected by Lorentz polarization and absorption with SADABS [S8]. The structure was solved using *SHELXS*-97 [S9], *via Patterson*, and refined with *SHELXL*-97 [S10]. **Table S1** contains the intensities collection and refinement data of the crystalline/molecular structure. *ORTEP-3* [S11] for Windows was used to draw the **Fig. 1**. The positional parameters of the H atoms bonded to C atoms in the pyridine ring were obtained geometrically, with the C-H bond distances fixed at 0.93 Å for Csp^2 and refined as riding on their respective C atoms with U_{iso}(H) = 1.2Ueq(Csp^2). H atoms bonded to C atoms in the methylene group were located geometrically and with the C-H bond distances fixed at 0.97 Å for Csp^3 and also with U_{iso}(H) = 1.2Ueq(Csp^3). The positional parameters of the atoms H1N and H2N bonded to N3 in $\{[CuPEP]ClO_4\}_n$ were obtained from a Fourier difference map and refined freely with an isotropic displacement parameter.

S2. Characterization of the ligand NaPEP and precursors

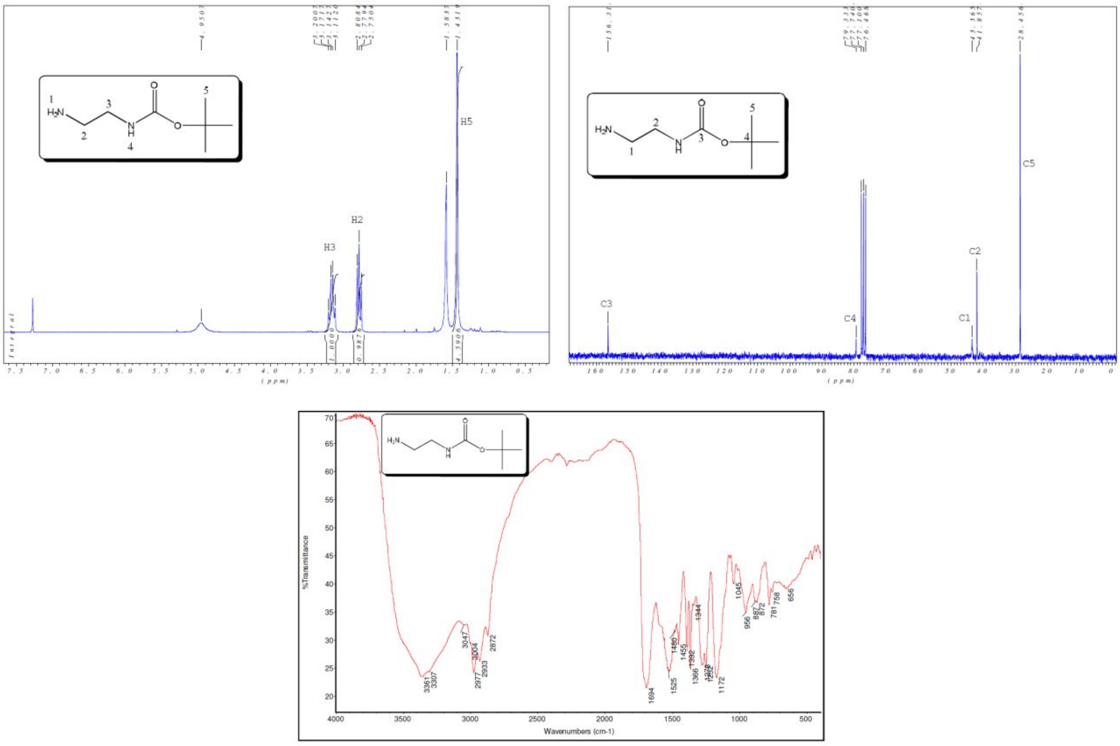

Fig. S1. ^{1}H NMR and $^{13}C\{^{1}H\}$ NMR spectra in $CDCl_3$, FTIR spectrum in KBr pellets, of *N*-tert-butoxycarbonylethylenediamine **1**.

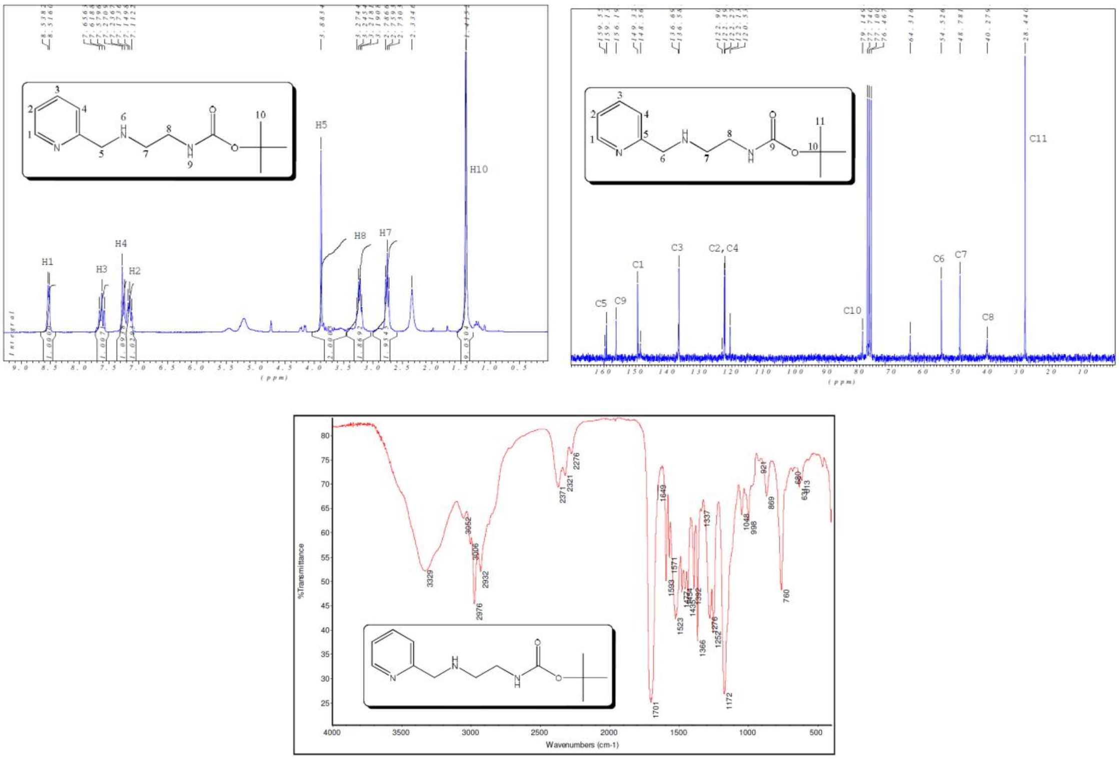

Fig. S2. ^{1}H NMR and $^{13}C\{^{1}H\}$ NMR spectra in $CDCl_3$, FTIR spectrum in KBr pellets, of *N*-tert-butoxycarbonyl-*N'*-(2-pyridylmethyl)ethylenediamine **2**.

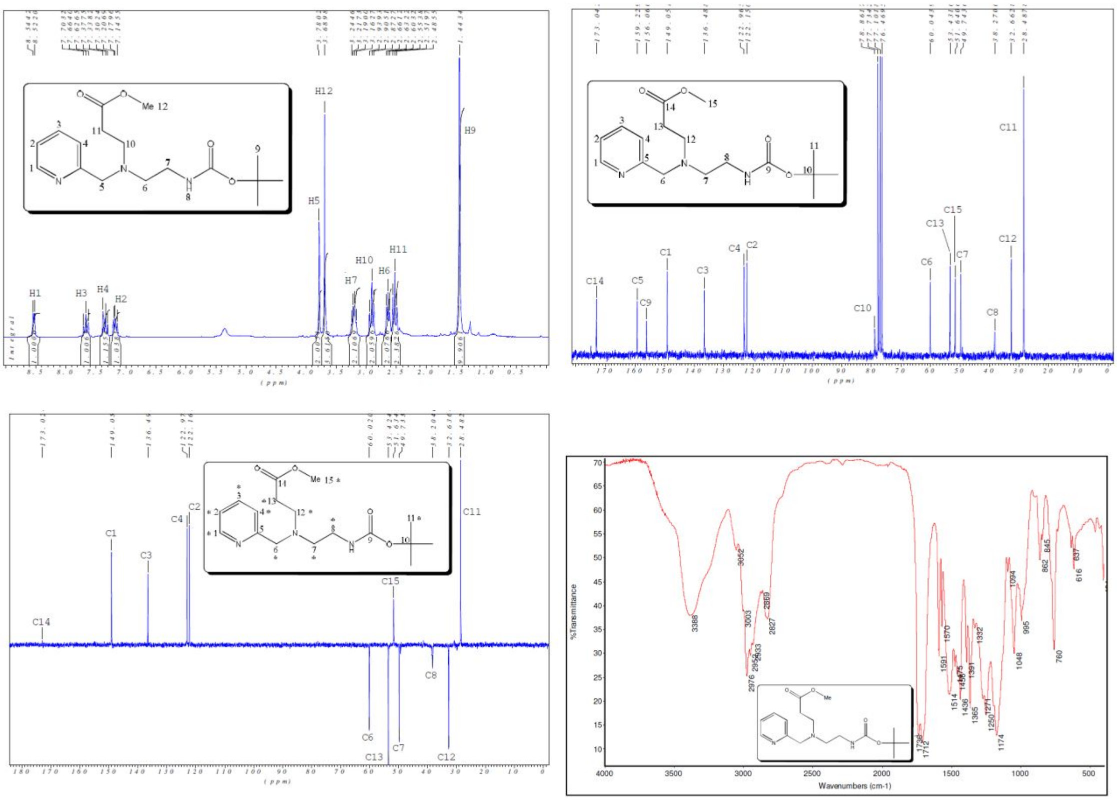

Fig. S3. ^{1}H NMR and ^{13}C NMR{^{1}H} spectra in $CDCl_3$, FTIR spectrum in KBr pellets, of *N*-tert-butoxycarbonyl-*N'*-(2-pyridylmethyl)ethylenediamine-*N'*-methylpropanoate **3**.

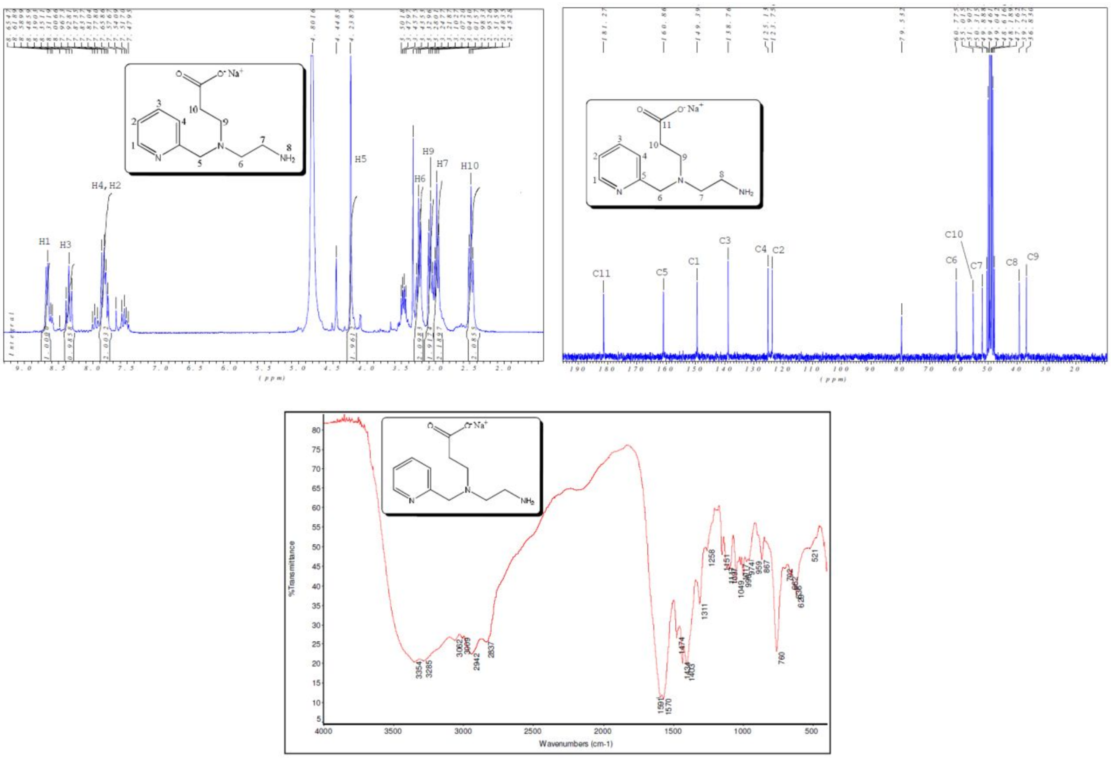

Fig. S4. ^{1}H NMR (D_2O) and ^{13}C{^{1}H} NMR (CD_3OD) spectra, FTIR spectrum in KBr pellets, of *N*-(2-pyridylmethyl)ethylenediamine-*N*-propanoate **NaPEP 4**.

S3. Characterization of $\{[CuPEP]ClO_4\}_n$

S3.1. Single crystal X-ray crystallography

Table S1. Crystallographic data for $\{[CuPEP]ClO_4\}_n$.

Empirical formula	$C_{11}H_{16}ClCuN_3O_6$
Formula weight	385.26
Temperature	295(2) K
Wavelength	0.71073 Å
Crystal system	monoclinic
Space group	*C* 1 *c* 1
Unit cell dimensions	a = 14.092(3) Å<br>b = 12.318(3) Å β = 104.15(3)°.<br>c = 9.1844(18) Å
Volume	1545.9(5) Å^3
Z	4
Density (calculated)	1.655 Mg/m^3
Absorption coefficient	1.617 mm^{-1}
F(000)	788
Crystal size	0.62 × 0.23 × 0.20 mm^3
Theta range for data collection	3.31 to 25.48°.
Index ranges	-17<=h<=17, -14<=k<=14, -11<=l<=10
Reflections collected	17322
Independent reflections	2831 [R(int) = 0.0315]
Completeness to theta	= 25.48° => 99.8 %
Max. and min. transmission	0.7381 and 0.4339
Refinement method	Full-matrix least-squares on F^2
Data / restraints / parameters	2831 / 2 / 248
Goodness-of-fit on F^2	1.087
Final R indices [I>2sigma(I)]	R_1 = 0.0287, wR_2 = 0.0738
R indices (all data)	R_1 = 0.0309, wR_2 = 0.0758
Largest diff. peak and hole	0.506 and -0.304 e.Å^{-3}

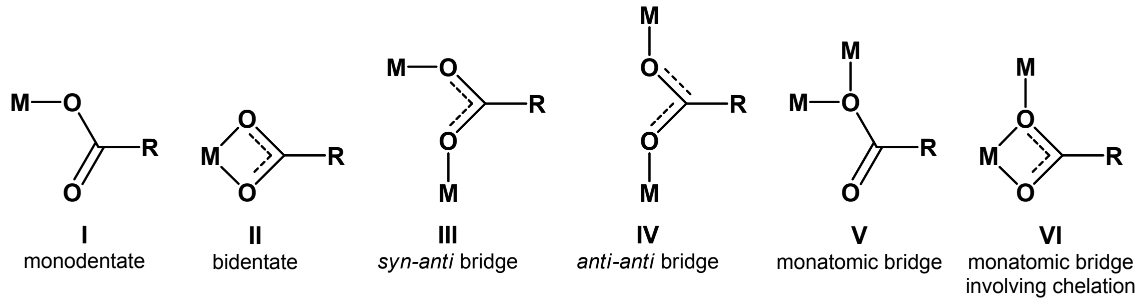

Scheme S1. Examples of carboxylate coordination modes [S12-S13].

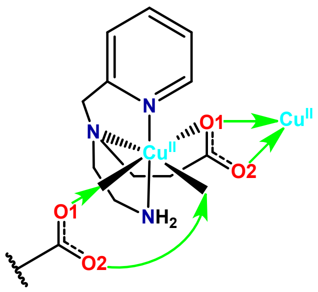

Scheme S2. Representation of the polymeric chain growth in $\{[CuPEP]ClO_4\}_n$.

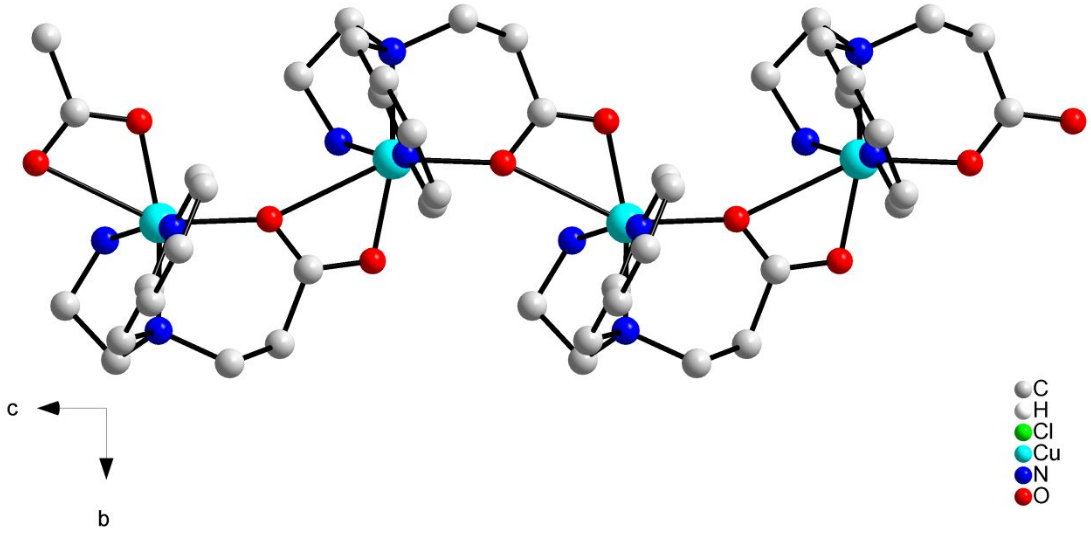

Fig. S5. One-dimensional polymeric chain of $\{[CuPEP]ClO_4\}_n$ along the [001] crystallographic direction. View along the crystallographic axis *a*. Perchlorate ions were omitted for clarity.

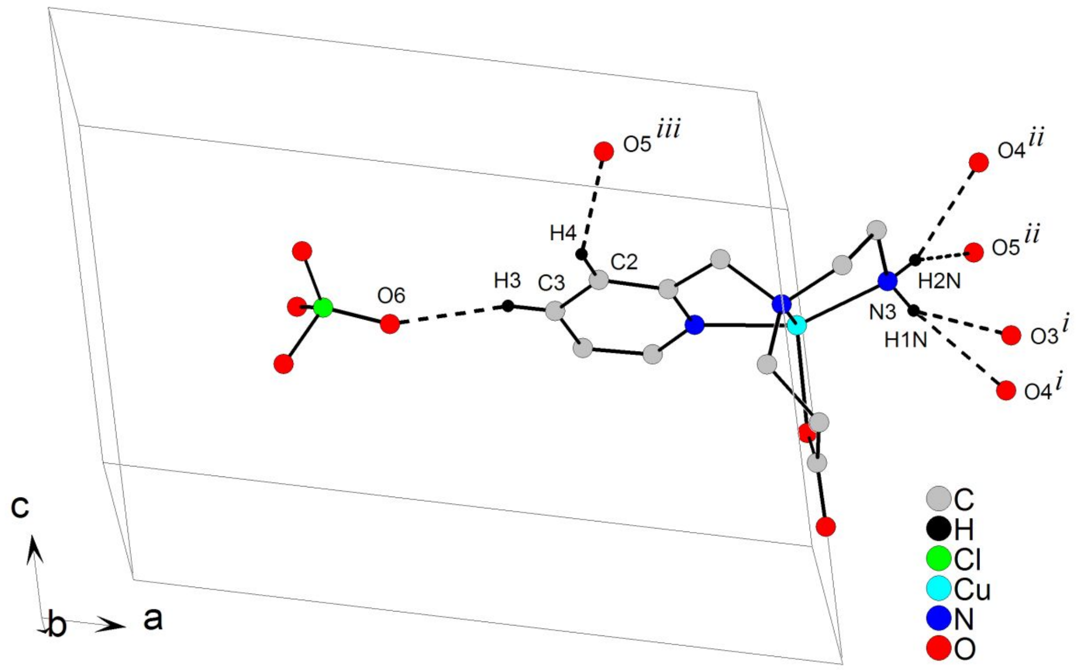

Fig. S6. Hydrogen bonds formed in $\{[CuPEP]ClO_4\}_n$.

Table S2. Geometric parameters for H-bonds in the crystal structure of $\{[CuPEP]ClO_4\}_n$.

D-H…A	d(D-H) (Å)	d(H…A) (Å)	d(D…A) (Å)	∠(D-H…A) (º)
N(3)-H(1N)…O(3)^{i}	0.78(4)	2.47(5)	3.085(7)	137(4)
N(3)-H(1N)…O(4)^{i}	0.78(4)	2.47(4)	3.211(7)	159(4)
N(3)-H(2N)…O(4)^{ii}	1.10(5)	2.32(5)	3.208(7)	137(4)
N(3)-H(2N)…O(5)^{ii}	1.10(5)	2.15(5)	3.211(9)	161(4)
C(2)-H(4)…O(5)^{iii}	0.93	2.42	3.197(10)	141
C(3)-H(3)…O(6)	0.93	2.36	3.263(7)	164

Symmetry code: (i): (1+x, y, z); (ii): (1+x, 2-y, 0.5+z); (iii): (0.5+x, 1.5-y, 0.5+z).

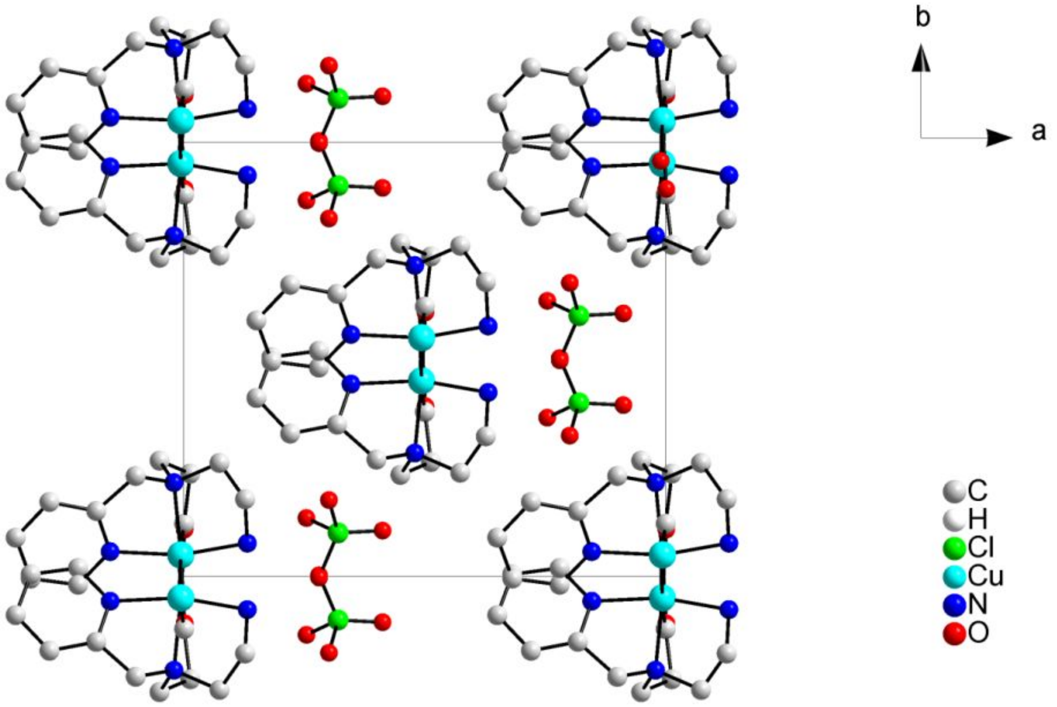

Fig. S7. Polymeric chain packing of $\{[CuPEP]ClO_4\}_n$ along [001] crystallographic direction. View along the crystallographic axis *c*.

Table S3. Structural data for one-dimensional Cu^{II} coordination polymers.

Ligands	Coordination polymer	CO_2-Cu^{II} CM[a]	Cu···Cu (Å)	Cu-O (Å)	C-O (Å)
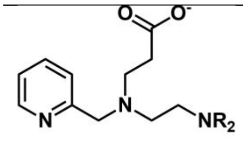 PEP-, R=H [this work]<br>L3OO(-), R=Me [S14]<br>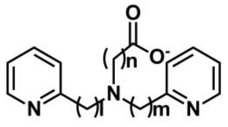	$\{[Cu^{II}(PEP)](ClO_4)\}_n$	V	4.751	1.976; 2.154; 2.696	1.259; 1.277
	$\{[Cu^{II}(L^3OO)][CF_3SO_3]\}_n\cdot H_2O$	III	5.281	1.938; 2.231	1.232; 1.284
	$\{[Cu^{II}(\mu\text{-pmea})](ClO_4)\cdot H_2O\}_n$	III	5.350	1.947; 2.246	1.277; 1.230
	$\{[Cu^{II}(\mu\text{-pmpa})](ClO_4)\cdot 2H_2O\}_n$	III	5.800	1.943; 2.393	1.261; 1.236
	$\{[Cu^{II}(\mu\text{-pmba})(H_2O)](ClO_4)\cdot 3H_2O\}_n$	I	9.363	1.927	1.223; 1.278
	$\{[Cu^{II}(\mu\text{-pmva})(H_2O)](ClO_4)\}_n$	I	9.745	1.969	1.203; 1.285
	$\{[Cu^{II}(\mu\text{-pmca})(H_2O)](ClO_4)\}_n$	I	10.401	1.973	1.227; 1.293
	$\{[Cu^{II}(L^1OO)](ClO_4)\}\cdot 2H_2O$	III	4.579	2.012; 2.133	1.266; 1.243
	$\{[Cu^{II}(L^2)](ClO_4)\}_n$	III	5.257	1.961; 2.203	1.219; 1.276
	$\{[Cu^{II}(L^3)(H_2O)](ClO_4)\}_n$	I	9.405	2.017	1.235; 1.286
	$\{[Cu^{II}(L^3)(ClO_4)]\}_n$	I	9.676	1.991	1.256; 1.200

l	m	n	ligand	Ref.
1	1	1	pmea	S15
1	1	2	pmpa	S15
1	1	3	pmba	S15
1	1	4	pmva	S16
1	1	5	pmca	S16
1	2	2	L1OO-	S17
2	2	2	L2	S18
2	2	3	L3	S18
2	2	4	L4	S18

[a]CM: coordination mode

L^1OO^- = 3-[(2-(pyridine-2-yl)ethyl){2-(pyridine-2-yl)methyl}amino]propionate; [S17]

L^3OO^- = 3-[(2-(pyridine-2-yl)ethyl){(dimethylamino)ethyl}amino]propionate); [S14]

Hpmva: bis(2-pyridylmethyl)amino-5-valeric acid; [S16]

Hpmca: bis(2-pyridylmethyl)amino-6-caproic acid; [S16]

pmea: bis(2-pyridylmethyl)amino-2-ethanoic acid; [S15]

pmpa: bis(2-pyridylmethyl)amino-3-propionic acid; [S15]

pmba: bis(2-pyridylmethyl)amino-4-butyric acid; [S15]

L2: 3-[bis-(2-pyridin-2-yl-ethyl)-amino]-propionic acid; [S18]

L3: 4-[bis-(2-pyridin-2-yl-ethyl)-amino]-butyric acid; [S18]

L4: 6-[bis-(2-pyridin-2-ylethyl)-amino]-hexanoic acid. [S18]

S3.2. X-ray powder diffraction and Rietveld Method

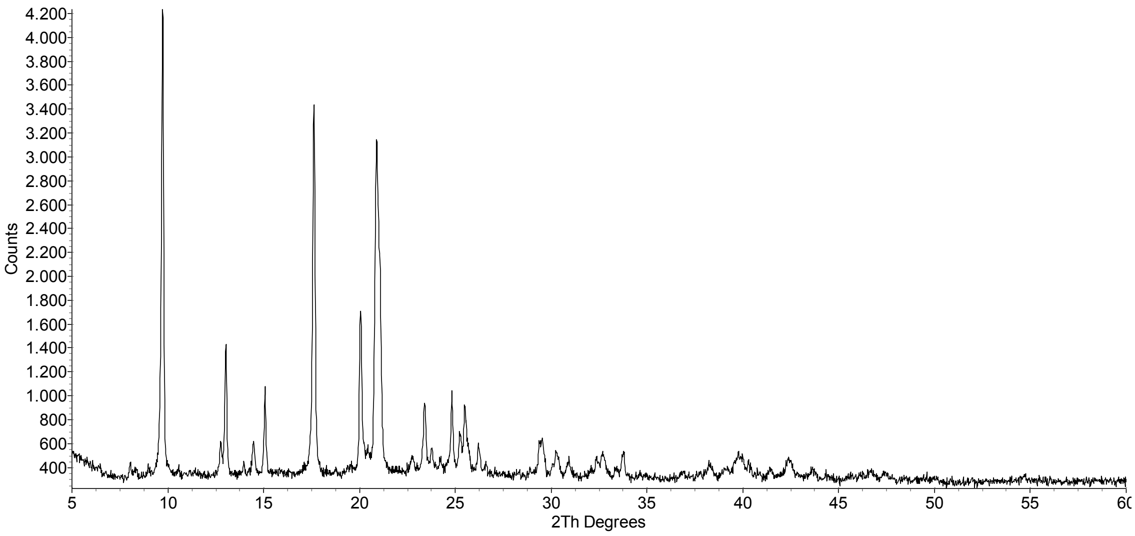

Fig. S8. X-ray standard powder result for $\{[CuPEP]ClO_4\}_n$.

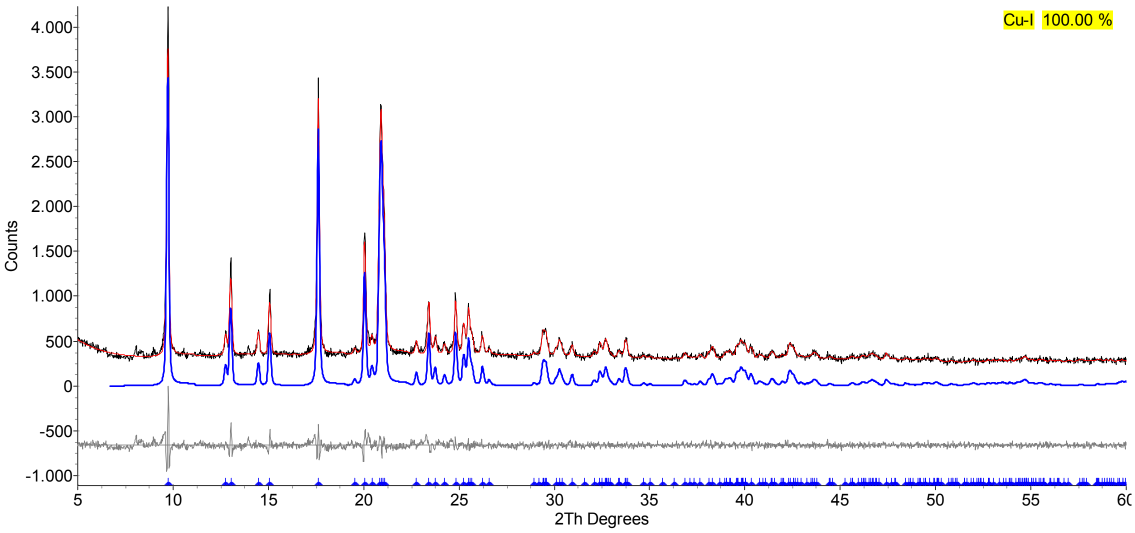

Fig. S9. Show refinement and calculate result for $\{[CuPEP]ClO_4\}_n$.

Table S4. The R-Values calculate.

R-values							
R exp:	4.848	Rwp:	6.932	Rp:	5.387	GOF:	1.430
R exp':	14.245	Rwp':	20.368	Rp':	23.717	DW:	1.203

(' = dash)

S3.3. FTIR spectroscopy

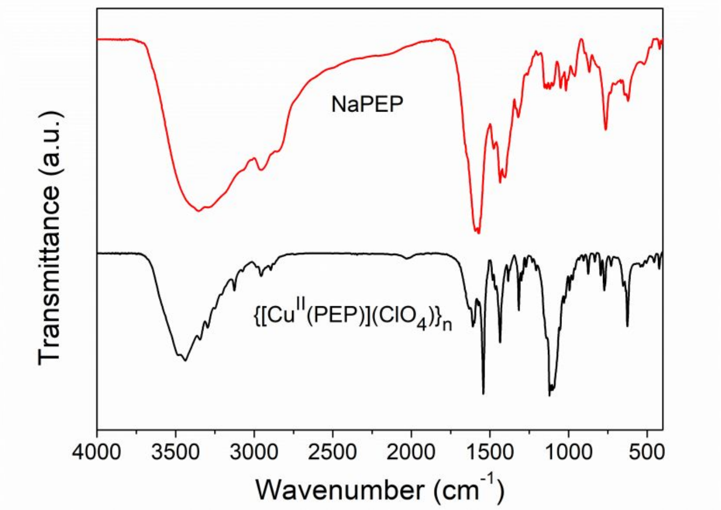

Fig. S10. FTIR of{[CuPEP]ClO_4}$_n$ and NaPEP ligand.

Table S5. Main Infrared bands of the {[CuPEP]ClO_4}$_n$.

ῡ (cm^{-1})	Functional group
3342, 3296	N–H stretching from primary amine
3182, 3075	C–H stretching from aromatic ring
2988, 2961, 2896	C–H stretching from aliphatic group
1542	C=O asymmetric stretching from carboxylate ion
1609, 1599, 1481, 1435	C–C and C–N stretching from aromatic pyridine ring
1385	C=O symmetric stretching from carboxylate ion
1317	C–C(=O)–O stretching
1100	ClO_4^- stretching
774	C–H out-of-plane symmetric bending from aromatic ring

S3.4. UV-VIS electronic spectroscopy

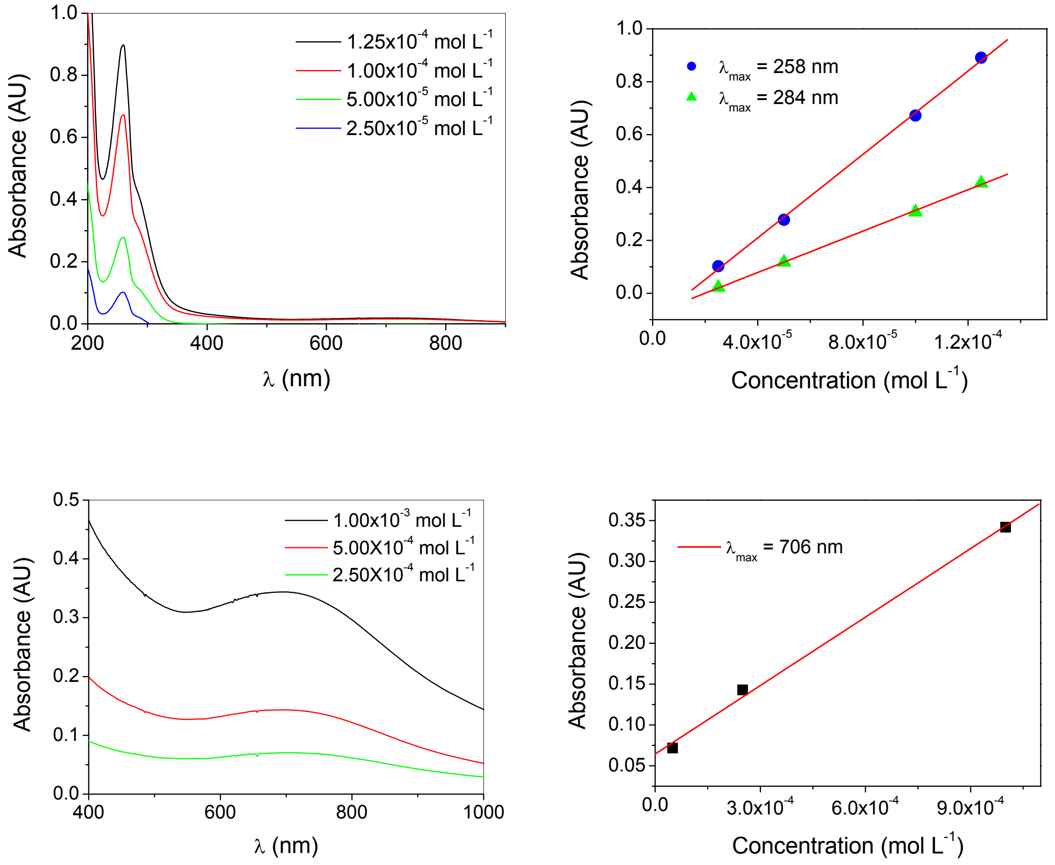

Fig. S11. UV-VIS spectra of $\{[CuPEP]ClO_4\}_n$ in CH_3CN and concentration versus absorbance plot for ε determination. Results: $\varepsilon = 7.83\times10^3$ dm^3 mol^{-1} cm^{-1} at 258 nm; $\varepsilon = 3.00\times10^3$ dm^3 mol^{-1} cm^{-1} at 284 nm; $\varepsilon = 1.31\times10^2$ dm^3 mol^{-1} cm^{-1} at 706 nm.

S3.5. ESI-MS

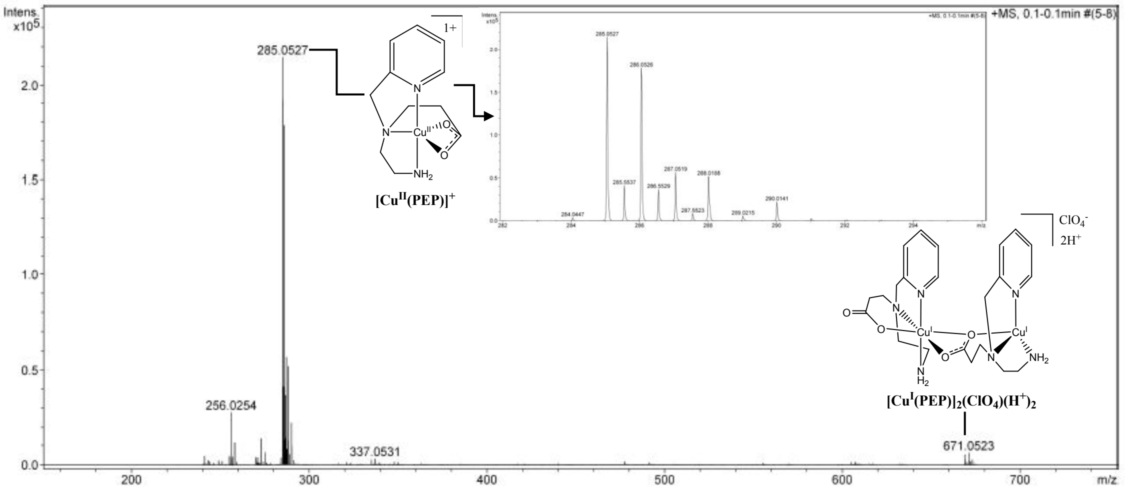

Fig. S12. ESI-MS spectrum of $\{[CuPEP]ClO_4\}_n$. Inlet: isotopic profile of the peak *m/z* 285.0527.

S3.6. Cyclic voltammetry

S3.6.1. CH_3CN solution

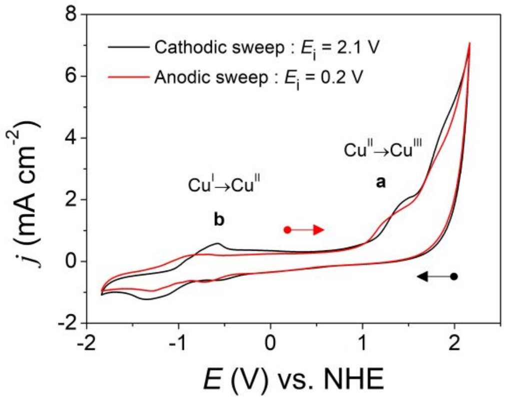

Fig. S13. Cyclic voltammograms of $\{[CuPEP]ClO_4\}_n$ at 1.0×10^{-3} mol L^{-1}, 100 mV s^{-1}, in CH_3CN, for cathodic and anodic sweeps.

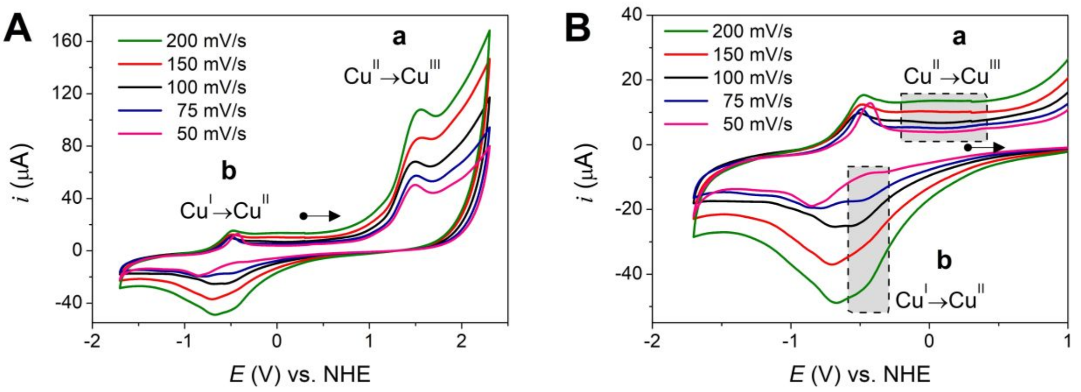

Fig. S14. Cyclic voltammograms of $\{[CuPEP]ClO_4\}_n$ in CH_3CN at different scan rates. Right: zoom.

Table S6. Electrochemical data for $\{[CuPEP]ClO_4\}_n$ process **a** for cyclic voltammetry experiments at CH_3CN from +2.3 V to −1.7 V vs NHE.

Scan rate (mV/s)	E_{pa} (V)	i_{pa} (A)
200	1.528	9.30×10^{-5}
150	1.511	7.46×10^{-5}
100	1.488	5.59×10^{-5}
75	1.483	4.67×10^{-5}
50	1.477	4.10×10^{-5}

Redox couple Fc/Fc^+ at 100 mV/s: ΔE = 100 mV; $E_{1/2}$ = 0.0986 V.

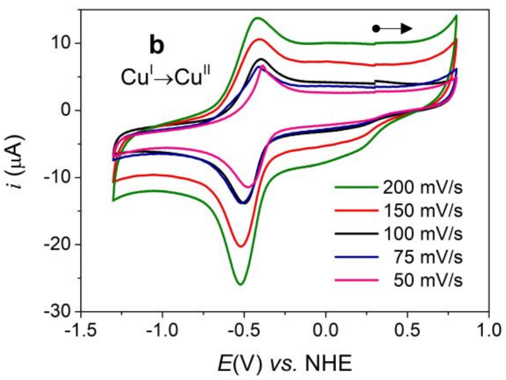

Fig. S15. Cyclic voltammograms of process **b** of $\{[CuPEP]ClO_4\}_n$ in acetonitrile at different scan rates.

Table S7. Electrochemical data for $\{[CuPEP]ClO_4\}_n$ process **b** for cyclic voltammetry experiments at CH_3CN from +0.8 V to −1.3 V vs NHE.

Scan rate (mV/s)	E_{pa} (V)	E_{pc} (V)	$E_{1/2}$ (V)	ΔE (mV)	i_{pa} (A)	i_{pc} (A)	$\lvert i_{pa}/i_{pc}\rvert$
200	−0.419	−0.524	−0.471	105	1.20×10^{-5}	-1.60×10^{-5}	0.75
150	−0.407	−0.521	−0.464	115	9.86×10^{-6}	-1.29×10^{-5}	0.76
100	−0.402	−0.511	−0.456	110	8.07×10^{-6}	-9.50×10^{-6}	0.85
75	−0.411	−0.516	−0.463	105	7.55×10^{-6}	-9.71×10^{-6}	0.78
50	−0.386	−0.475	−0.430	89	7.77×10^{-6}	-8.09×10^{-6}	0.96

Redox couple Fc/Fc$^+$ at 100 mV/s: ΔE = 100 mV; $E_{1/2}$ = 0.0986 V.

Chemical reactions coupled to electron transfer mechanisms:

E_r:$Ox + ne^- \rightleftharpoons Red$ eq S1

E_i:$Ox + ne^- \longrightarrow Red$ eq S2

C_r:$Z \rightleftharpoons O$ eq S3

C_{cat}:$R + Z \xrightarrow{cat} O$ eq S4

Diffusive current – Randles-Sevcik equation:

$$i_d = 0.4463 nFA[Cu]\sqrt{\frac{nFvD_{Cu}}{RT}}$$ eq S5

n is the electron transferred in the noncatalytic reaction, F is Faraday's constant, A is the area of the electrode in cm^2, [Cu] is the bulk concentration of the catalyst in mol cm^3, *v* is the scan rate in V s^{-1}, D_{Cu} is the diffusion coefficient in cm^2 s^{-1}, *R* is the gas constant, and *T* is the absolute temperature.

S3.6.2. Buffer solution

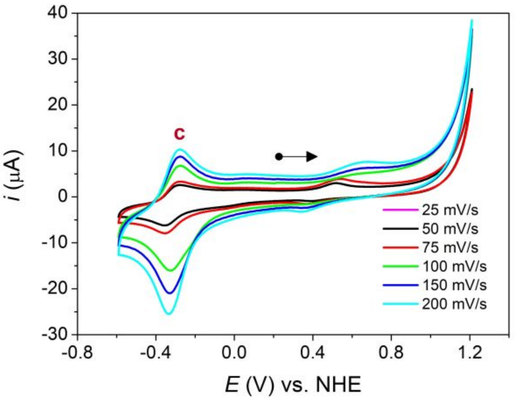

Fig. S16. Cyclic voltammograms of $\{[CuPEP]ClO_4\}_n$ at 1 mM in buffer at pH 12.5 in glassy carbon electrode WE.

Table S8. Cyclic voltammetry data for process **c** of $\{[CuPEP]ClO_4\}_n$ at 1 mol L^{-1} at buffer (0.1 mol L^{-1} phosphate) at different pH 12.5 from +1.22 V to –0.6 V vs NHE, at glassy carbon WE.

E_{pa}(c) (V)	E_{pc}(c) (V)	$E_{1/2}$(c)(V)	ΔE (V)
-0.286	-0.361	-0.323	0.075

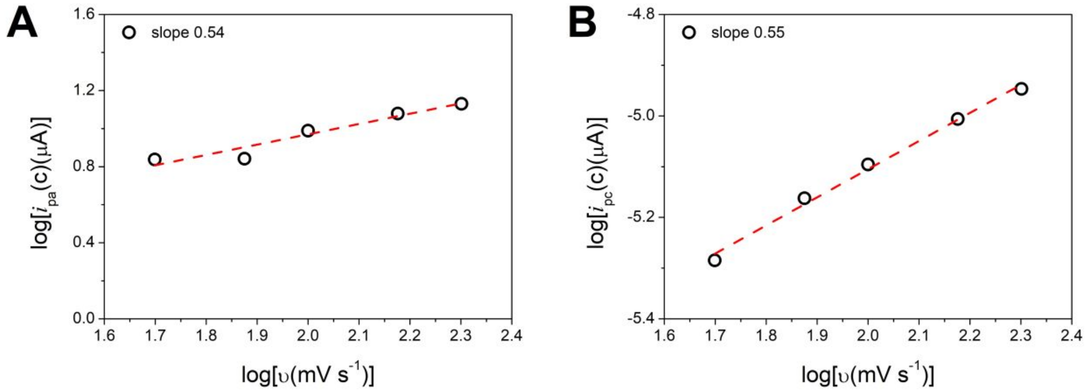

Fig. S17. Electrochemical data for process **c** of $\{[CuPEP]ClO_4\}_n$ in buffer at pH 12.5 and different scan rates. (A) Plot of log(i_{pa}) versus log(v). (B) Plot of log(i_{pc}) versus log(v).

Table S9. Diffusion coefficients for process **c** of $\{[CuPEP]ClO_4\}_n$ extracted from the Randles-Sevcik plot of i_{pa} (A) vs $v^{1/2}$ (V s^{-1}) in buffer at pH 12.5. Electrode area 0.0314 cm^2; $C^0 = 1.0\times10^{-3}$ mol dm^{-3}.

Slope i_{pa}	D (i_{pa}) cm^2 s^{-1}	Slope i_{pc}	D (i_{pc}) cm^2 s^{-1}
3.26×10^{-5}	1.49×10^{-5}	-2.70×10^{-5}	1.02×10^{-5}

Table S10. Potentials in V vs NHE in buffer.

**Compound**	**pH**	E_{pa} **(V)**	E_{pc} **(V)**	$E_{1/2}$ **(V)**	**Ref**
$\{[CuPEP]ClO_4\}_n$ - **c**	12.5	*-0.286*	*-0.361*	*-0.323*	This work
$[(bztpen)Cu](BF_4)_2$	11.5	-	-	-0.12	[S19]
$[Cu(pyalk)_2]$	12.5	-	-	-0.2	[S20]

PEP = *N*-(2-pyridylmethyl)ethylenediamine-*N*-propanoate
pyalk: 2-pyridinyl-2-propoxide
bztpen = *N*-benzyl-*N*,*N'*,*N'*-tris(pyridin-2-ylmethyl)ethylenediamine

S4. Electrocatalytic OER

S4.1. CV curves – Only OER from 0.4 to 1.4 V vs. NHE

Table S11. Overpotential (η) at different current density (j) of OER activity catalyzed by 5.0 mM $\{[CuPEP]ClO_4\}_n$ at FTO in 0.1 mol L^{-1} phosphate buffer at pH 12.5.

			η (mV)		
**E^0 (V)**	***j* (mA/cm^2)**				
	**0.1**	**0.2**	**1.0**	**3.0**	**4.0**
**0.4925**	394	436	543	638	721

Table S12. Overpotential in mV for copper(II) catalysts.

Catalyst	pH	η (mV)	*j* ($mA\ cm^{-2}$)	Ref
$\{[CuPEP]ClO_4\}_n$	12.5	394[a]	0.1	This work
		436[a]	0.2	This work
		488[b]	1	This work
		598[b]	2	This work
		697[b]	3	This work
		938[b]	5	This work
$[(bztpen)Cu](BF_4)_2$	11.5	440	0.2	[S19]
		960	3	[S19]
$[(dbzbpen)Cu(OH_2)](BF_4)_2$	11.5	570	0.2	[S19]
	11.5	900	3	[S19]
$[L1Cu_2(\mu\text{-}OH)](BF_4)_3$	12	770	1	[S21]
	12	980	3	[S21]
$[L2Cu(OH_2)](BF_4)_2$	12	870	1	[S21]
	12	1100	3	[S21]
$[Cu_2\text{-}P_o]_n$	9.2	573	0.1	[S22]
	9.2	644	1	[S22]
	10.2	509	0.1	[S22]
	10.2	563	1	[S22]
Cu-Im	14	500	10	[S23]
Cu-mIm	14	520	10	[S23]
Cu-bIm	14	560	10	[S23]

[a]: calculated from the LSV from 0.4 to 1.4 V vs NHE, at 100 mV s^{-1} (**Fig. S14**, **Table S9**).
[b]: calculated from the LSV from -1.3 to 1.7 V vs NHE, at 100 mV s^{-1} (**Fig. S15**, **Table S10**).
PEP = *N*-(2-pyridylmethyl)ethylenediamine-*N*-propanoate
bztpen = *N*-benzyl-*N,N',N'*-tris(pyridin-2-ylmethyl)ethylenediamine
dbzbpen = *N,N'*-dibenzyl-*N,N'*-bis(pyridin-2-ylmethyl)ethylenediamine
L1 = N,N′-dimethyl-N,N′-bis{2-[bis(2-pyridinylmethyl)amino]ethyl}-ethane-1,2-diamine
L2 = N,N-dimethyl-N′,N′-bis(2-pyridylmethyl)ethane-1,2-diamine)
Imidazole (Im, 99 %), 2-methylimidazole (mIm, 99 %), benzimidazole (bIm, 98 %)

Table S13. Overpotential in mV for heterogeneous copper(II) catalysts.

Catalyst	pH	η (mV)	*j* (mA cm^{-2})	Tafel (mV dec^{-1})	FE	Stability (h)	Ref
$\{[CuPEP]ClO_4\}_n$ Film after 100 CVs	12.5	724	4	448	-	-	This work
$\{[CuPEP]ClO_4\}_n$ Film after 5 mA cm^{-2}/3 h	12.5	768	4	278	-	3	This work
H_2-Cu_2Cat/FTO from Cu_2L1 Heterogeneous copper oxide obtained from electrodeposition of Cu(II) complex	9.2	500	0.1	71	96 %	4 h/+1.2 V vs. Ag/AgCl reaching 0.9 mA/cm2	[S25]
		630	1				
Cu@1/GC RDE Heterogeneous MOF	13	410	1	122	-	-	[S26]
Cu@3/GC RDE Heterogeneous MOF	13	420	1	123	-	-	[S26]
Cu-Im/carbon paper Heterogeneous coordination polymer	14	500	10	215		2 h/10 mA/cm2 reaching η = 520 mV	[S27]
Cu-mIm/carbon paper Heterogeneous coordination polymer	14	520	10	258		2 h/10 mA/cm2 reaching η = 550 mV	[S27]
Cu-bIm/carbon paper Heterogeneous coordination polymer	14	560	10	286		2 h/10 mA/cm2 reaching η = 570 mV	[S27]

L1 = Robson-type macrocyclic
Cu@1 and Cu@3: obtained from postsynthetic transmetallation of the MOFs $\{(Co(L1)(TA)(H_2O)_2)\cdot 2H_2O\}n$ and $\{(Co(L_2)_2(TA))\cdot 4H_2O\}n$, respectively (L1 and L2), aromatic dicarboxylate (1,4-benzendicarboxylate (1,4-bdc) (TA)).
Imidazole (Im, 99 %), 2-methylimidazole (mIm, 99 %), benzimidazole (bIm, 98 %)

S4.2. CV curves – OER and Cu(II)

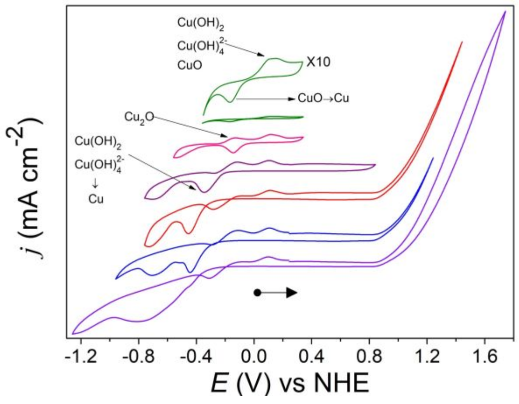

Fig. S18. Cyclic voltammetry of 5.0 mM {[CuPEP]ClO_4}$_n$, FTO WE, 0.1 mol L^{-1} phosphate buffer at pH 12.5, at different potential windows at 100 mV s^{-1}. Assignment according reference [S24].

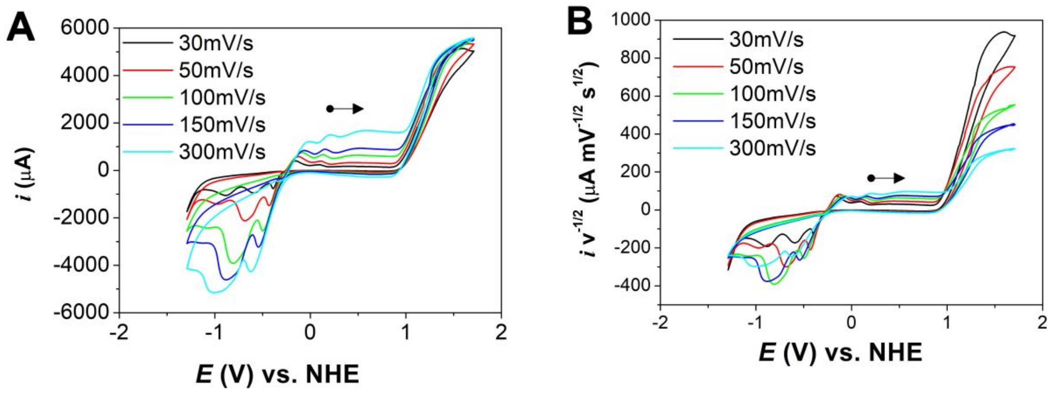

Fig. S19. Cyclic voltammetry (forward scan) of OER activity catalyzed by 5.0 mM {[CuPEP]ClO_4}$_n$ at FTO in 0.1 mol L^{-1} phosphate buffer at pH 12.5.

Table S14. Overpotential (η) at different current density (j) of OER electrocatalysis by 5.0 mM {[CuPEP]ClO_4}$_n$ at FTO in 0.1 mol L^{-1} phosphate buffer at pH 12.5

η (mV)				
j (mA cm^{-2})				
**1**	2	3	4	5
**488**	598	697	784	938

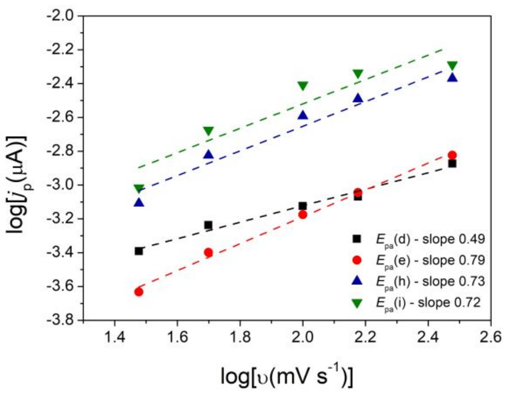

Fig. S20. Electrochemical data for the processes **d, e, h** and **i** of $\{[CuPEP]ClO_4\}_n$ in buffer at pH 12.5 and different scan rates at FTO. Plot of log(i_{pa}) versus log(v).

Table S15. Diffusion coefficients for the processes **d, e, h** and **i** extracted from the Randles-Sevcik plot [i(A) vs v(V/s)] of $\{[CuPEP]ClO_4\}_n$ in buffer at pH 12.5. Electrode area 1.0 cm^2; $C^0 = 5.0\times10^{-3}$ mol dm^{-3}.

Slope (d)	***D*** **(d) cm^2 s^{-1}**	Slope (e)	***D*** **(e) cm^2 s^{-1}**	Slope (h)	***D*** **(h) cm^2 s^{-1}**	Slope (i)	***D*** **(i) cm^2 s^{-1}**
2.38×10^{-3}	3.1×10^{-6}	3.36×10^{-3}	6.3×10^{-6}	9.25×10^{-3}	4.7×10^{-5}	1.11×10^{-2}	6.8×10^{-5}

The catalytic peak current i_{cat} in CV can be described by the model E_rC_{cat} (eq S1 and S4) a reversible electrochemical reaction coupled to a catalytic reaction, as shown in eq S6.

$$i_{cat} = n_{cat}FA[Cu]\sqrt{k_{cat}D_{Cu}} \qquad \text{eq S6}$$

where $n_{cat} = 4$ is the number of electrons transferred in the catalytic reaction (OER), [Cu] is the bulk catalyst concentration (mol L^{-1}), k_{cat} is the apparent first-order rate constant (s^{-1}), D_{Cu} is the diffusion coefficient of the catalyst (cm^2 s^{-1}), and i_{cat} (A) was taken at 1.6 V vs NHE.

The apparent first-order rate constant of the catalytic OER, k_{cat}, is also referred as turnover frequency (TOF) of the catalyst and was calculated from the ratio of eq S6 and eq S5 to give eq 7, where i_d is the plateau current density of the non-catalytic wave Cu(I)/Cu(II) (process **d**), and v is the scan rate in V s^{-1}.

$$i_{cat}/i_d = 1.436(k_{cat}/v)^{1/2} \qquad \text{eq S7}$$

S4.3. Stability tests

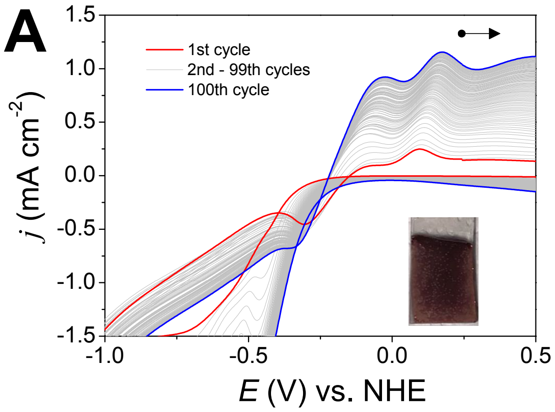

Fig. S21. A: Zoom of **Fig. 12A** of the paper; Inset: picture of the film.

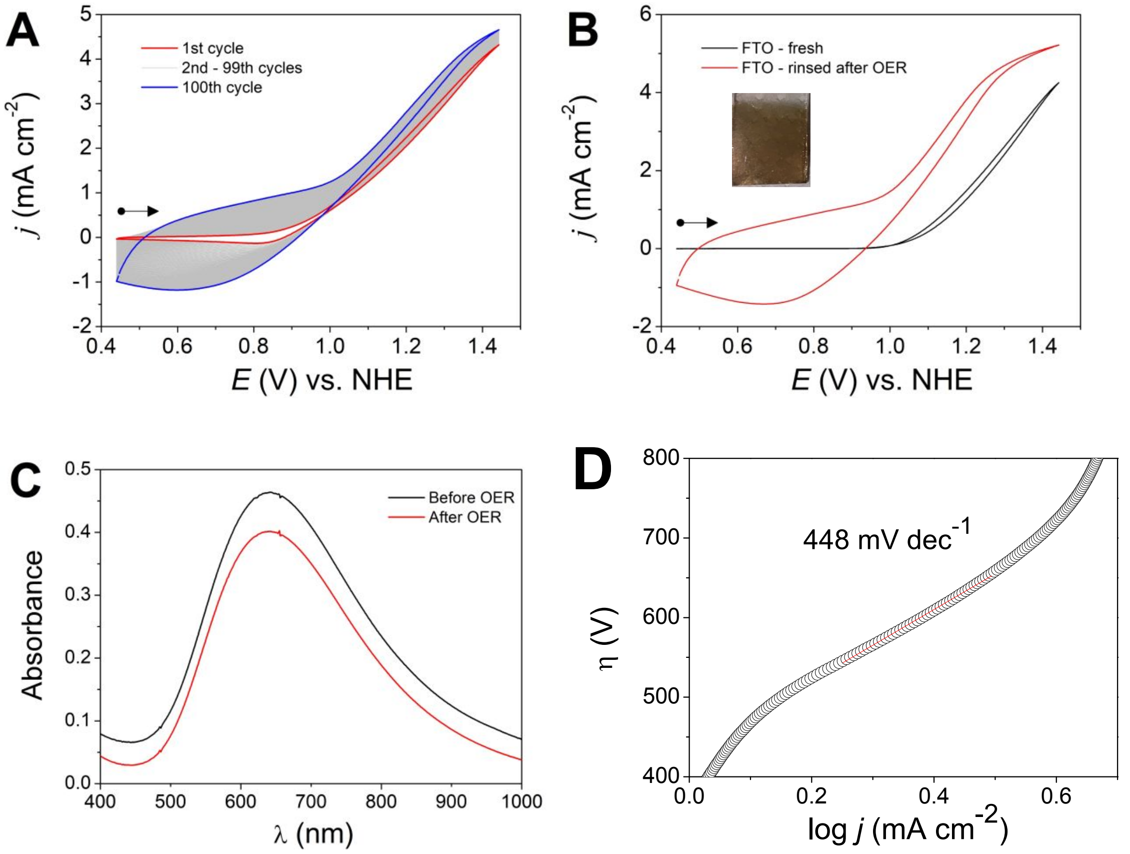

Fig. S22. Cyclic voltammetry in 0.1 mol L^{-1} phosphate buffer at pH 12.5, 100 mV s^{-1} at a narrow potential window. A: 100 cycles of CV of OER activity catalyzed by 5.0×10^{-3} mol L^{-1} $\{[CuPEP]ClO_4\}_n$, FTO WE. B: OER test in pure electrolyte using the rinsed FTO WE after 100 CVs cycles. Inset: picture of the film with white and black background. C: UV-Vis of the electrolyte solution before and after the 100 CVs cycles. B: Tafel plot corresponding LSV shown in **Fig. S22B**.

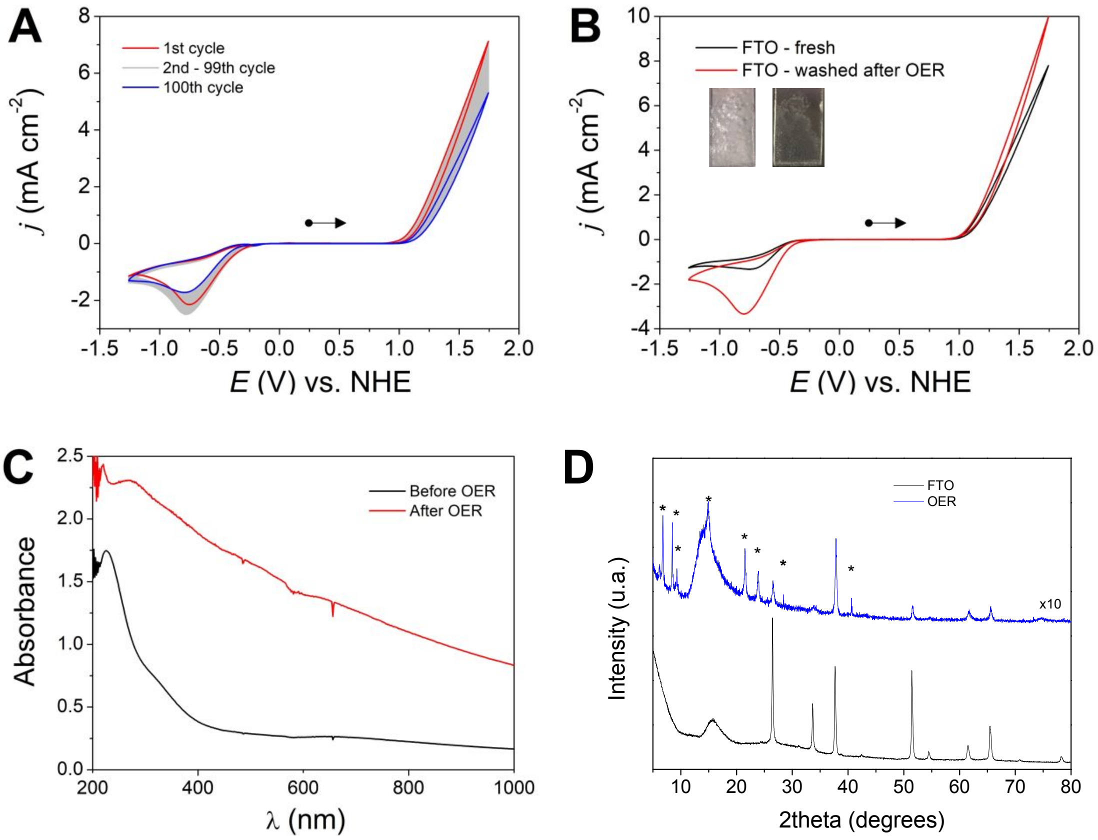

Fig. S23. Cyclic voltammetry in 0.1 mol L^{-1} phosphate buffer at pH 12.5, 100 mV s^{-1} at a wide potential window. A: 100 cycles of CV of OER activity catalyzed by 5.0×10^{-3} mol L^{-1} $Cu(ClO_4)_2$, FTO WE. B: OER test in pure electrolyte using the rinsed FTO WE after 100 CVs cycles. Inset: picture of the film with white and black background. C: UV-Vis of the electrolyte solution after and before 100 CV cycles. D: XRD of the FTO WE film after 100 CV cycles.

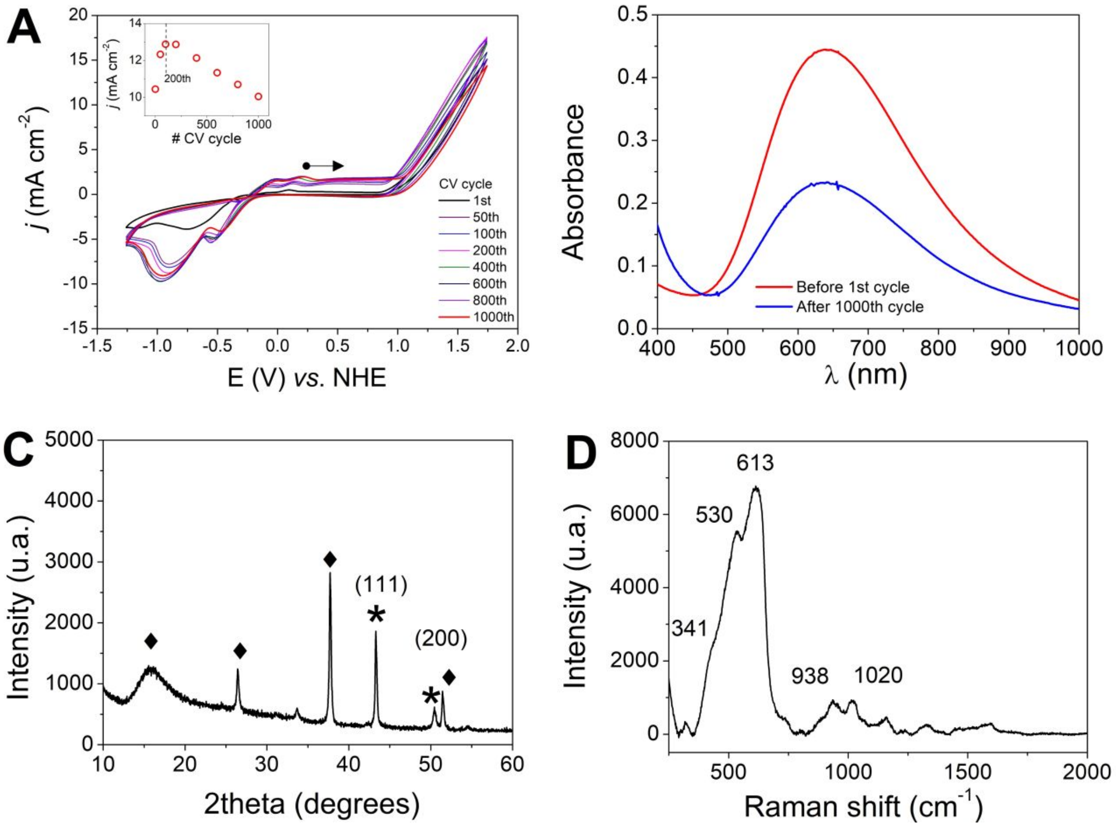

Fig. S24. A: 1000 cycles of cyclic voltammetry of OER activity catalyzed by 5.0×10^{-3} mol L^{-1} $\{[CuPEP]ClO_4\}_n$, FTO WE, 0.1 mol L^{-1} phosphate buffer at pH 12.5, 100 mV s^{-1} at a wide potential window; Inset: current density at 1.5 V in function of cycling. B: UV-Vis of the electrolyte solution after and before 1000 CVs cycles. XR diffractogram (C) and Raman spectra (D) of the FTO films obtained after 1000 cycles of cyclic voltammetry of OER activity catalyzed by 5.0 mM $\{[CuPEP]ClO_4\}_n$, 0.1 mol L^{-1} phosphate buffer at pH 12.5.

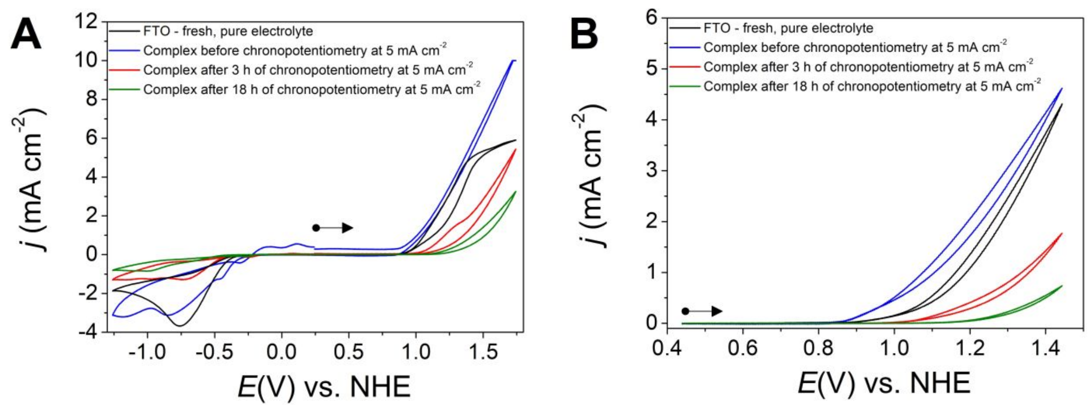

Fig. S25. OER test of the electrolyte solution 5.0 mol L^{-1} $\{[CuPEP]ClO_4\}_n$, FTO WE, 0.1 mol L^{-1} phosphate buffer at pH 12.5, 100 mV s^{-1} before and after the chronopotentiometry at 5 mA cm^{-2} for 3 h and 18 h. A and B are related to the CV in different regions. After the chronopotentiometry test, a fresh FTO was used to measure the LSV.

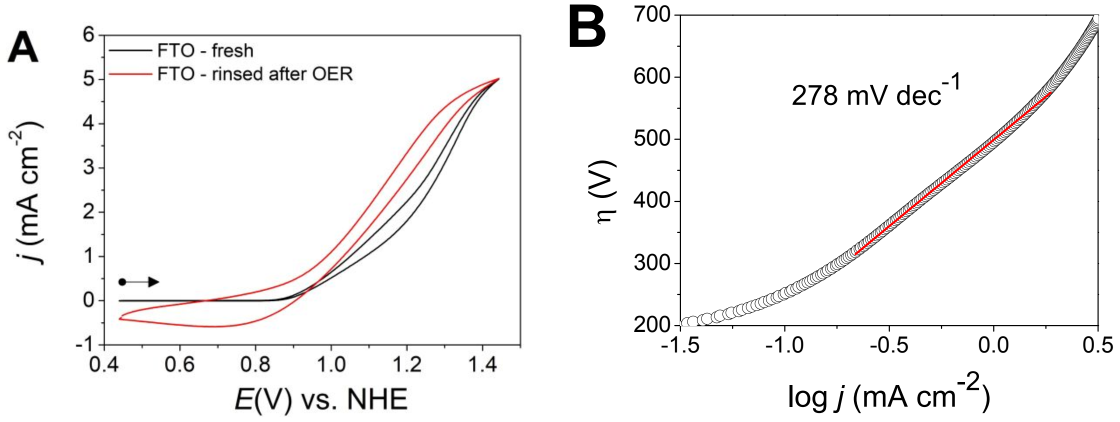

Fig. S26. A: CV at 100 mV s^{-1} of OER test in pure electrolyte using the rinsed FTO WE after the chronopotentiometry at 5 mA cm^{-2} for 3 h, related to **Fig. 13A** of the paper. B: Corresponding Tafel plot.

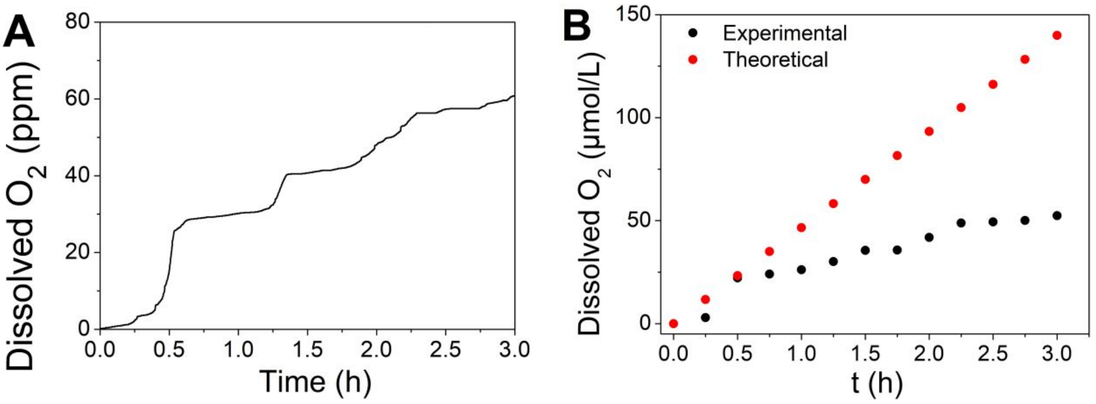

Fig. S27. A: Dissolved oxygen evolution of the chronopotentiometry at 5 mA cm^{-2} for 3 h catalyzed by 5.0 mol L^{-1} {[CuPEP]ClO_4}$_n$, FTO WE, 0.1 mol L^{-1} phosphate buffer at pH 12.5. B: Corresponding Faradaic efficiency.

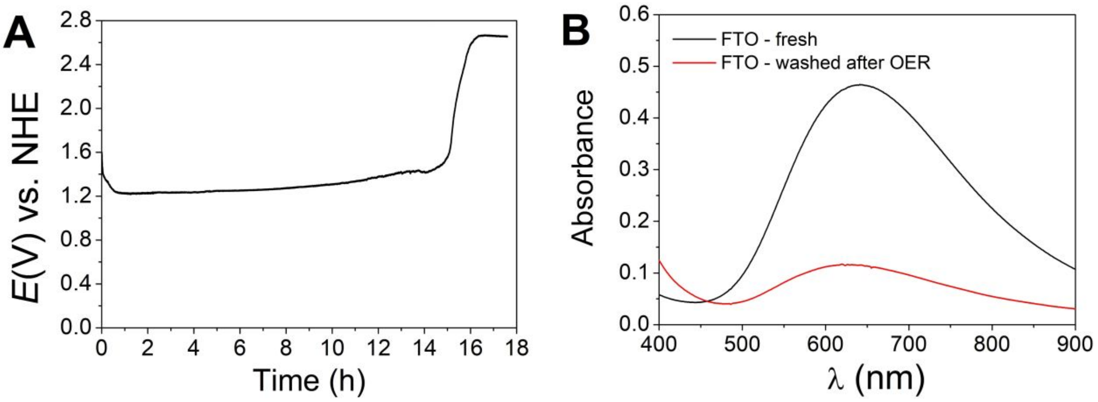

Fig. S28. A: Chronopotentiometry at 5 mA cm^{-2} for 18 h catalyzed by 5.0 mol L^{-1} {[CuPEP]ClO_4}$_n$, FTO WE, 0.1 mol L^{-1} phosphate buffer at pH 12.5. B: UV-Vis of the electrolyte solution after and before the chronopotentiometry.

S4.4. Electrochemical characterization of the film after the stability tests

The electrochemically active surface area (ECSA), mass activity (MA) and specific activity (SA) were calculated for the film after chronopotentiometry at 5 mA cm^{-2}.

ECSA was calculated by cyclic voltammetry (CV) measured in a non-Faradaic region at different scan rates, where the electrochemical double layer capacitance (C_{dl}) was determined using eq S1.

$$i_p[mA] = v[Vs^{-1}] \times C_{dl}[mF] \qquad \text{eq S1}$$

Being i_p the anodic (i_a) or cathodic (i_c) charge current and v the scan rate, the angular coefficient of the straight line generated between i_c or i_a and v will be equivalent to the C_{dl}. The average of the C_{dl} values measured from i_a or i_c was used for the calculation of ECSA, shown in eq S2:

$$ECSA[cm^2] = C_{dl}[mF]/C_s[mF\ cm^{-2}] \qquad \text{eq S2}$$

C_s is the specific charge density, and this value describes the smooth surface of the electrode. In alkaline solutions, a value of 40×10^{-3} mF cm^{-2} can be estimated [28,29].

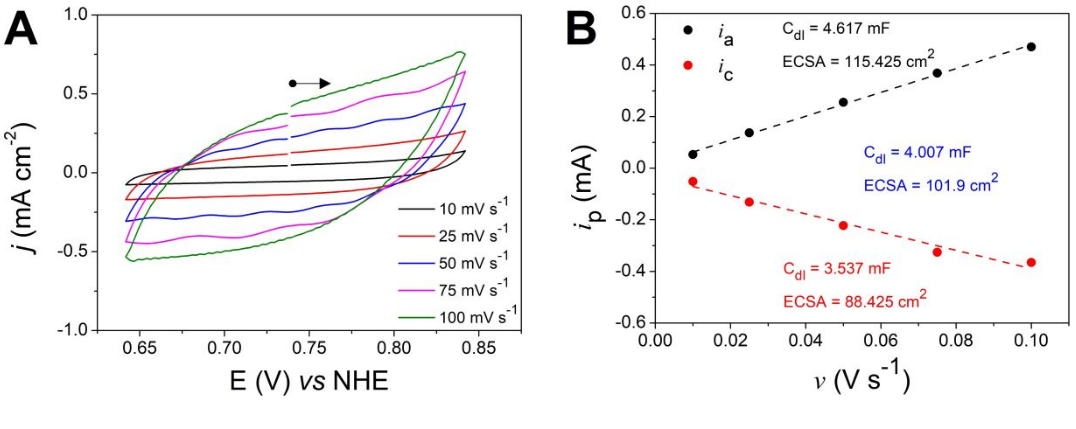

Fig. S29. A: CVs in non-Faradaic region at ±10 mV around OCP at different scan rates in pure 0.1 mol L^{-1} phosphate buffer at pH 12.5, of the film produced by chronopotentiometry at 5 mA cm^{-2} produced from a solution of 5.0×10^{-3} mol L^{-1} {[CuPEP]ClO_4}$_n$, FTO WE, 0.1 mol L^{-1} phosphate buffer at pH 12.5. B: Plot of current versus scan rate for anodic or cathodic waves at 0.75 V vs NHE for the calculation of the C_{dl} and ECSA.

MA values are determined by dividing the measured current density at η = 800 mV (E = 1.3 V vs NHE) from OER LSV (**Fig. S25**) by the Cu loaded at the electrode, m = 2.64×10^{-4} g cm^{-2} determined from AAS. This calculation can be expressed by eq S3:

$$MA[A\ g^{-1}] = j[A\ cm^{-2}]/m[g\ cm^{-2}] \quad \text{eq S3}$$

SA values were determined by dividing the current i (μA) at η = 800 mV (E = 1.3 V vs NHE) from OER LSV by the ECSA. This calculation can be expressed by eq S4 [30]:

$$SA[\mu A\ cm^{2}] = i[_{\mu}A]/ECSA[cm^{2}] \quad \text{eq S4}$$

The Tafel slopes (eq S5) allow verifying the electron transfer kinetics at the anode and the cathode. For Tafel calculations, data was collected from OER LSV.

$$\eta[V] = b[mV\ dec^{-1}] \times \log j[mA\ cm^{-2}] + a \quad \text{eq S5}$$

where j is the current density, η is the overpotential and b is the Tafel slope.

S4.5. Characterization of the film after the stability tests

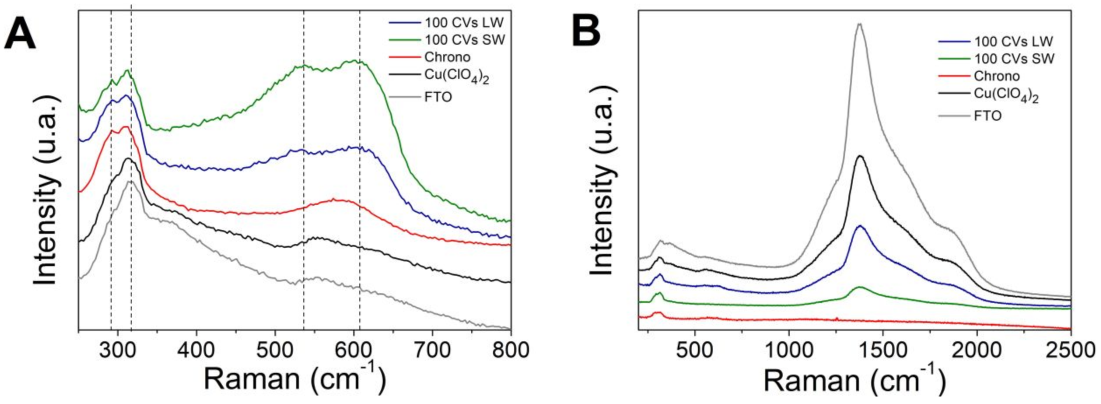

Fig. S30. Raman spectra, of the FTO films obtained after 100 cycles of cyclic voltammetry (Fig. 12) and chronopotentiometry at 5 mA cm^{-2} (Fig. 13) of OER activity catalyzed by 5.0 mM $\{[CuPEP]ClO_4\}_n$, 0.1 mol L^{-1} phosphate buffer at pH 12.5.

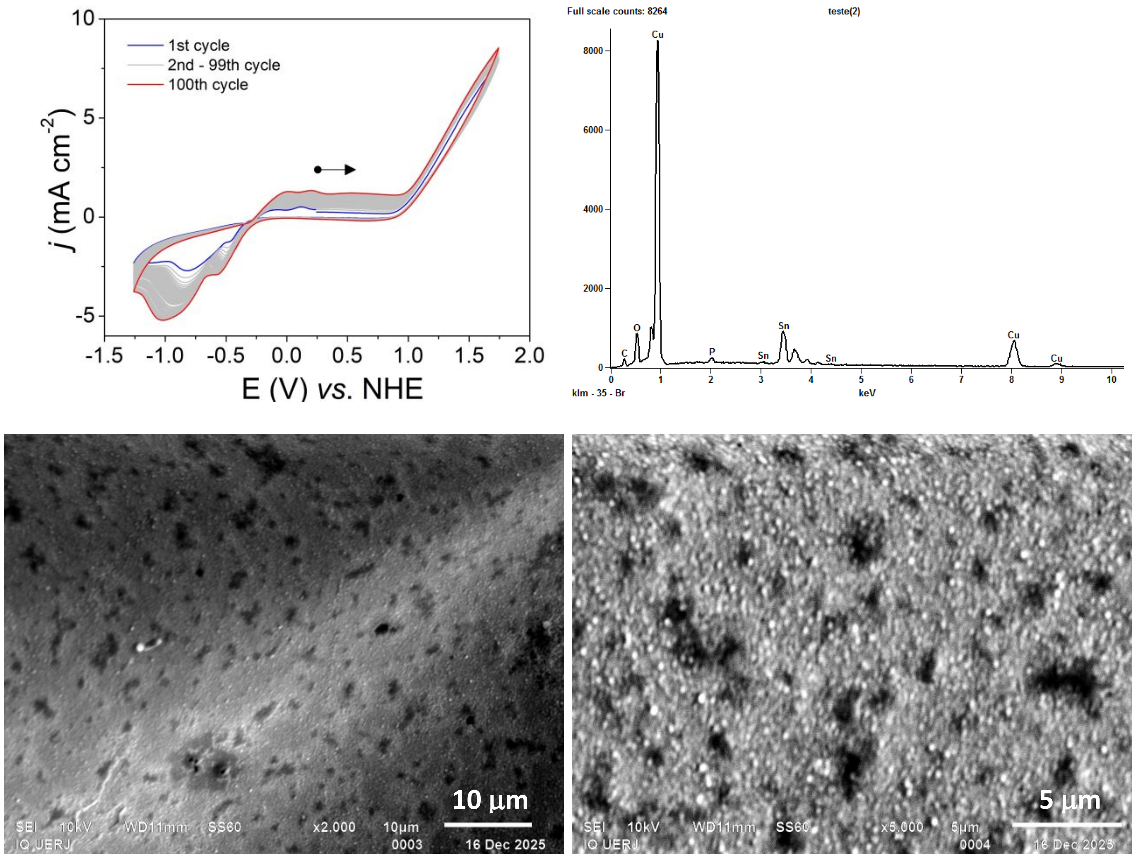

Fig. S31. SEM images and SEM-EDS analysis of the FTO film obtained after 100 cycles of cyclic voltammetry of OER activity catalyzed by 5.0 mM $\{[CuPEP]ClO_4\}_n$, 0.1 mol L^{-1} phosphate buffer at pH 12.5, 100 mV s^{-1}.

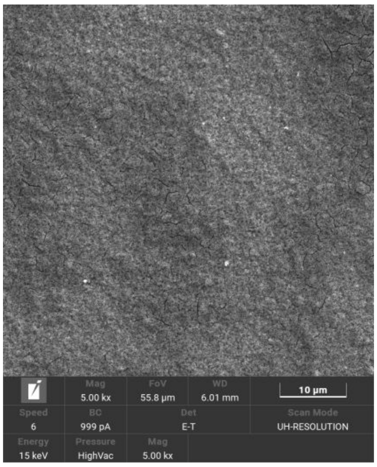
Mag 5.00 kx
FoV 55.8 µm
WD 6.01 mm
10 µm
Speed 6
BC 999 pA
Det E-T
Scan Mode UH-RESOLUTION
Energy 15 keV
Pressure HighVac
Mag 5.00 kx

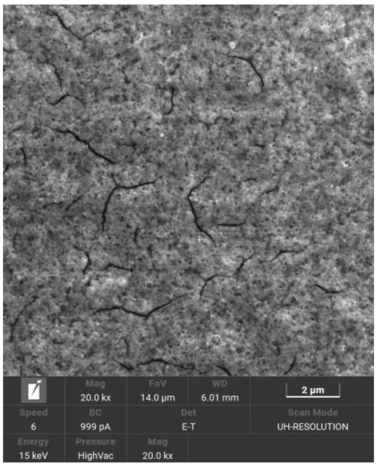
Mag 20.0 kx
FoV 14.0 µm
WD 6.01 mm
2 µm
Speed 6
BC 999 pA
Det E-T
Scan Mode UH-RESOLUTION
Energy 15 keV
Pressure HighVac
Mag 20.0 kx

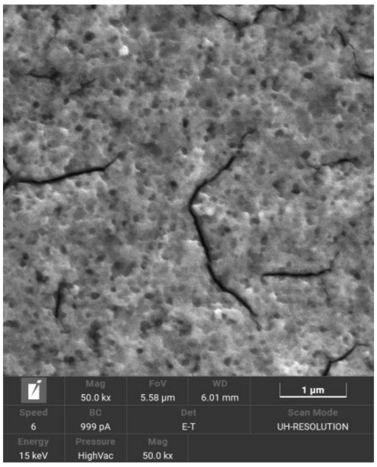
Mag 50.0 kx
FoV 5.58 µm
WD 6.01 mm
1 µm
Speed 6
BC 999 pA
Det E-T
Scan Mode UH-RESOLUTION
Energy 15 keV
Pressure HighVac
Mag 50.0 kx

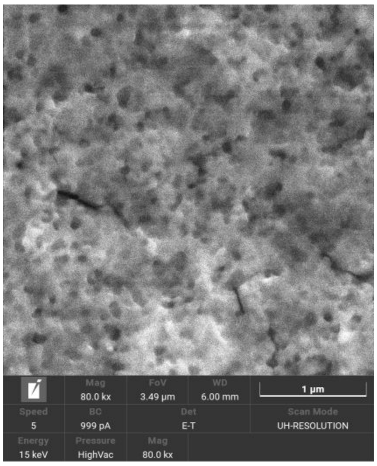
Mag 80.0 kx
FoV 3.49 µm
WD 6.00 mm
1 µm
Speed 5
BC 999 pA
Det E-T
Scan Mode UH-RESOLUTION
Energy 15 keV
Pressure HighVac
Mag 80.0 kx

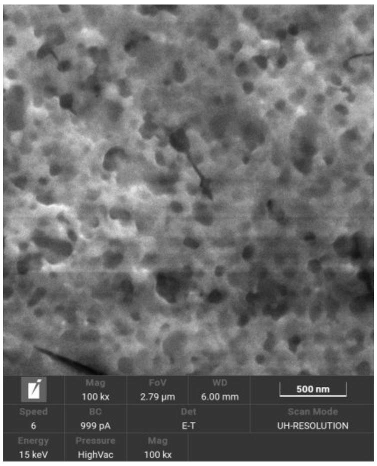
Mag 100 kx
FoV 2.79 µm
WD 6.00 mm
500 nm
Speed 6
BC 999 pA
Det E-T
Scan Mode UH-RESOLUTION
Energy 15 keV
Pressure HighVac
Mag 100 kx

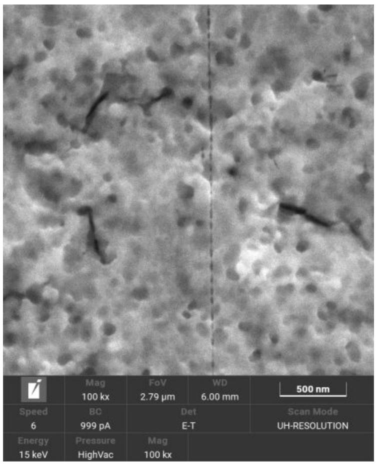
Mag 100 kx
FoV 2.79 µm
WD 6.00 mm
500 nm
Speed 6
BC 999 pA
Det E-T
Scan Mode UH-RESOLUTION
Energy 15 keV
Pressure HighVac
Mag 100 kx

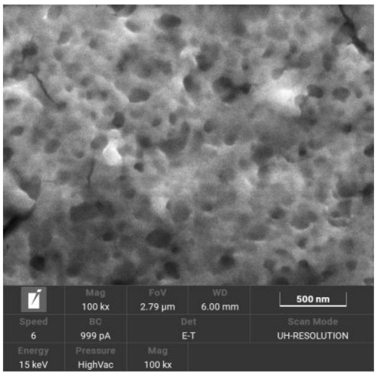
Mag 100 kx
FoV 2.79 µm
WD 6.00 mm
500 nm
Speed 6
BC 999 pA
Det E-T
Scan Mode UH-RESOLUTION
Energy 15 keV
Pressure HighVac
Mag 100 kx

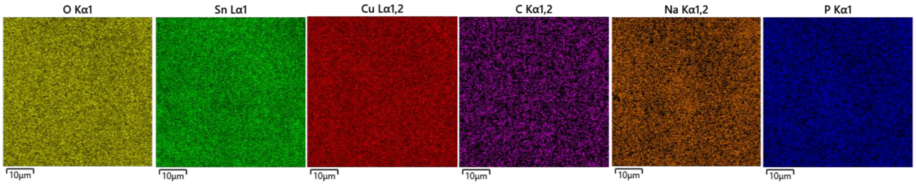
O Kα1
Sn Lα1
Cu Lα1,2
C Kα1,2
Na Kα1,2
P Kα1
10μm
10μm
10μm
10μm
10μm
10μm

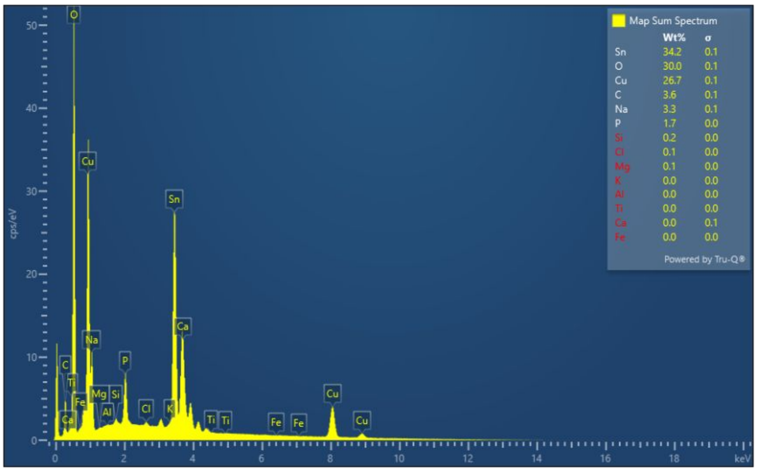

Fig. S32. SEM images and SEM-EDS analysis of the FTO film obtained after chronopotentiometry at 5 mA cm^{-2} for 1h30min catalyzed by 5.0 mM {[CuPEP]ClO$_4$}$_n$, 0.1 mol L^{-1} phosphate buffer at pH 12.5.

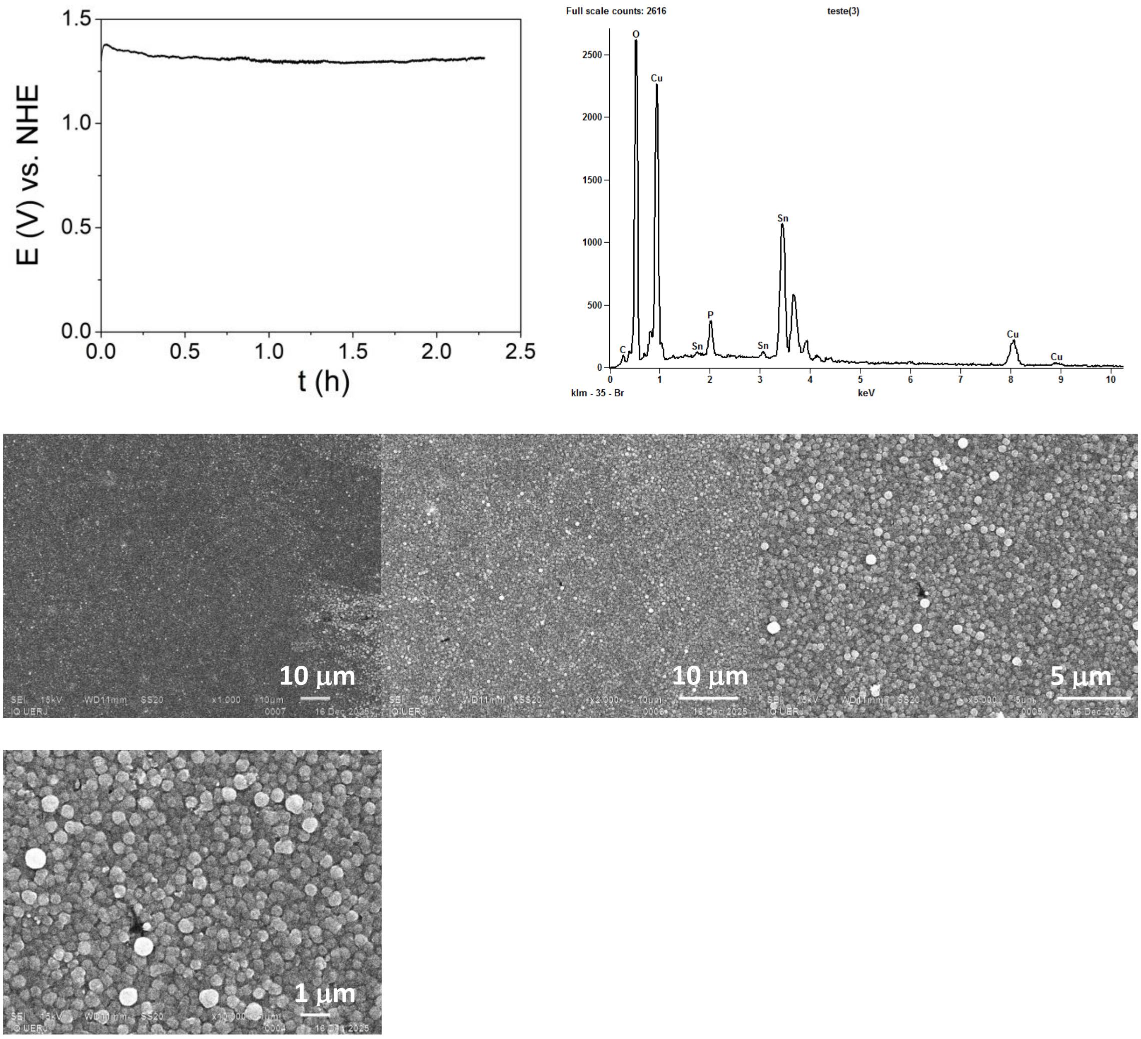

Fig. S33. SEM images and SEM-EDS analysis of the FTO film obtained after chronopotentiometry at 5 mA cm^{-2} for 2h10min catalyzed by 5.0 mM $\{[CuPEP]ClO_4\}_n$, 0.1 mol L^{-1} phosphate buffer at pH 12.5.

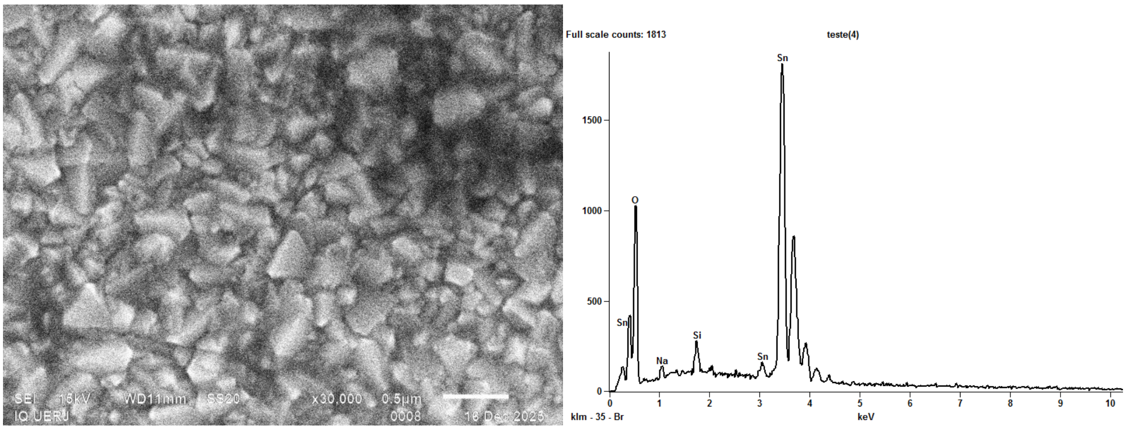

Fig. S34. SEM images and SEM-EDS analysis of the FTO film obtained after chronopotentiometry at 5 mA cm^{-2} for 16 h catalyzed by 5.0 mM $\{[CuPEP]ClO_4\}_n$, 0.1 mol L^{-1} phosphate buffer at pH 12.5.

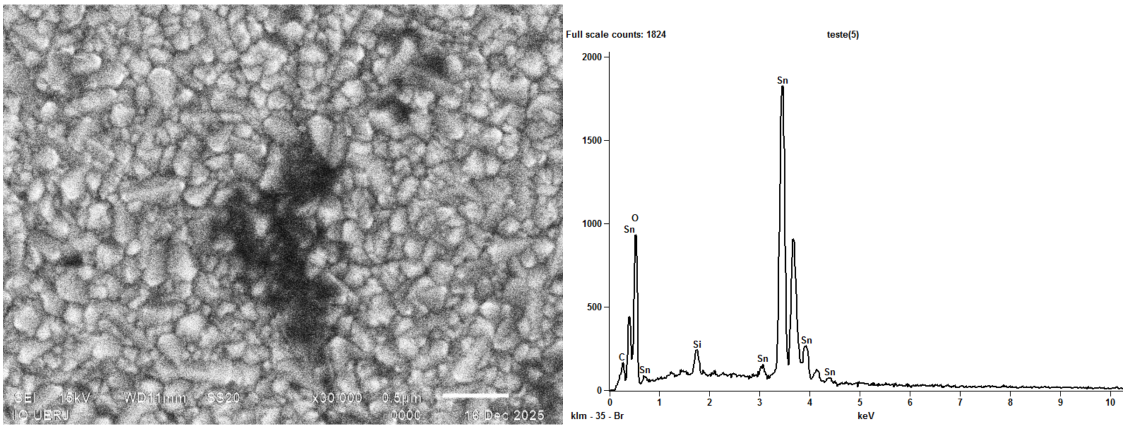

Fig. S35. SEM images and SEM-EDS analysis of a fresh FTO substrate for comparison.

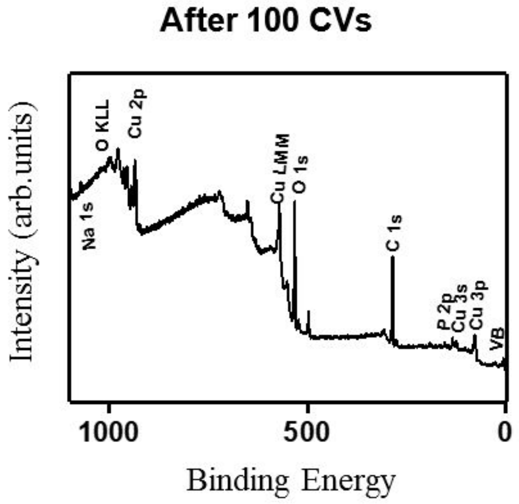

Fig. S36. XPS survey spectra of the samples after the stability OER tests catalyzed by 5.0 mM $\{[CuPEP]ClO_4\}_n$, 0.1 mol L^{-1} phosphate buffer at pH 12.5: (a) 100 cycles of CV and (b) chronopotentiometry at 5 mA cm^{-2} for 3 h. All major elemental contributions are labeled. All spectra were collected at RT under UHV conditions.

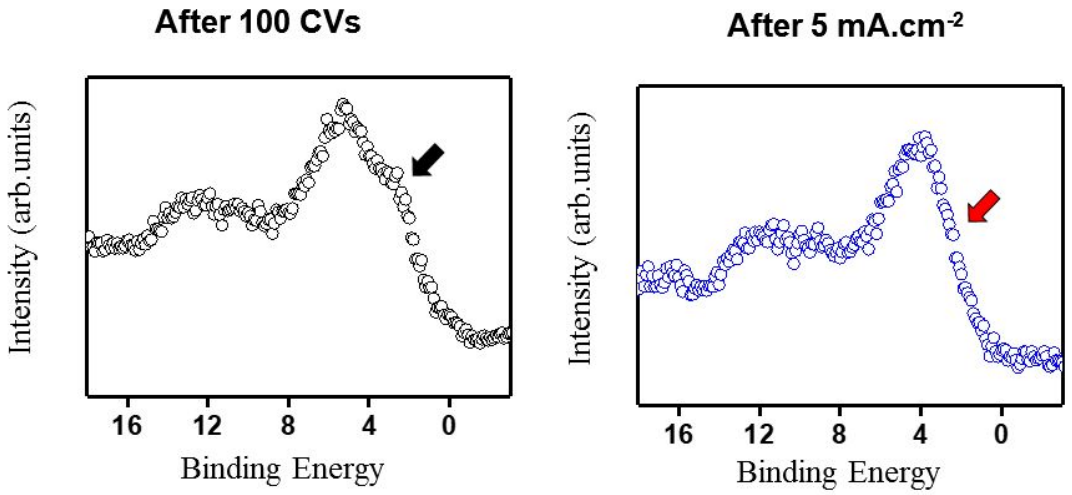

Fig. S37. XPS valence band spectra of the samples after the stability OER tests catalyzed by 5.0 mM $\{[CuPEP]ClO_4\}_n$, 0.1 mol L^{-1} phosphate buffer at pH 12.5: (a) 100 cycles of CV and (b) chronopotentiometry at 5 mA cm^{-2} for 3 h. Black and red arrows highlight differences in the line shape, respectively. All spectra were collected at RT under UHV conditions.

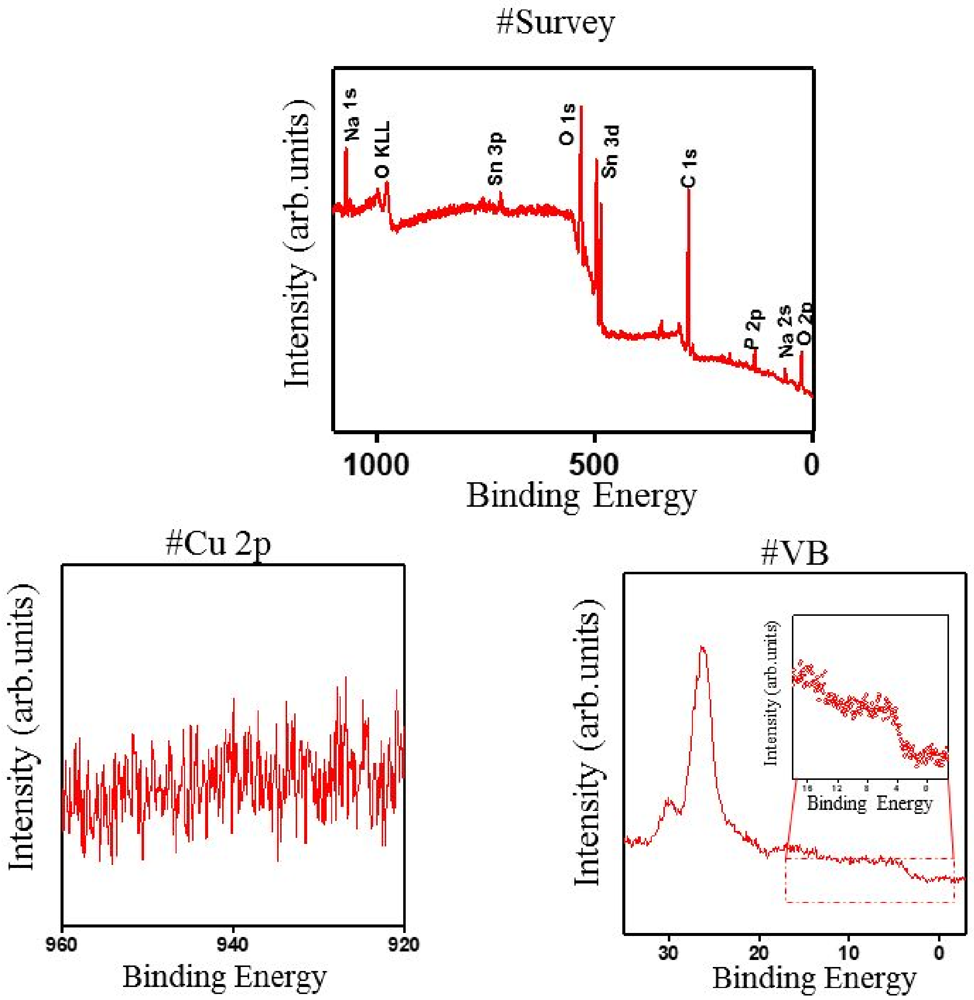

Fig. S38. XPS analysis of the samples after the stability OER tests catalyzed by 5.0 mM $\{[CuPEP]ClO_4\}_n$, 0.1 mol L^{-1} phosphate buffer at pH 12.5 by chronopotentiometry at 5 mA cm^{-2} for 12 h. All major elemental contributions are labeled. All spectra were collected at RT under UHV conditions.

S5. References

[S1] Fairley, N.; Fernandez, V.; Richard-Plouet, M.; Guillot-Deudon, C.; Walton, J.; Smith, E.; Flahaut, D.; Greiner, M.; Biesinger, M.; Tougaard, S. *Appl. Surf. Sci. Adv.* 2021, *5*, 100112.

[S2] Scofield, J. H. J. Electron Spectrosc. Relat. Phenom. 1976, 8, 129– 137.

[S3] Tanuma, S.; Powell, C. J.; Penn, D. R. Surf. Interface Anal. 1994, 21, 165– 176.

[S4] Nonius, B. COLLECT. Delft, The Netherlands 1998.

[S5] Duisenberg, A. J. M.; Hooft, R. W. W.; Schreurs, A. M. M.; Kroon, J. Accurate Cells from Area-Detector Images. J Appl Crystallogr 2000, 33, 893–898.

[S6] Duisenberg, A. J. M. J Appl Crystallogr 1992, 25, 92–96.

[S7] Duisenberg, A. J. M.; Kroon-Batenburg, L. M. J.; Schreurs, A. M. M. An Intensity Evaluation Method: EVAL-14. J Appl Crystallogr 2003, 36, 220–229.

[S8] G. M. Sheldrick. SADABS: Program for Empirical Absorption Correction of Area Detector Data. University of Göttingen, Germany 1996.

[S9] Sheldrick, G. M. SHELXS97: Program for Crystal Structure Solution. University of Göttingen, Germany 1997.

[S10] Sheldrick, G. M. SHELXL97: Program for Crystal Structure Refinement. University of Göttingen, Germany 1997.

[S11] Farrugia, L. J. ORTEP-3 for Windows - a Version of ORTEP-III with a Graphical User Interface (GUI). 1997, No. 30, 565.

[S12] G. B. Deacon, R. J. Phillips, Coord. Chem. Rev. 1980, 33, 227-250.

[S13] K. Nakamoto, 5a Ed., 1997, John Wiley & Sons, Inc., New York.

[S14] H. Arora, F. Lloret, R. Mukherjee, Eur. J. Inorg. Chem. 2009, 3317–3325.

[S15] K.-Y. Choi, Y.-M. Jeon, H. Ryu, J.-J. Oh, H.-H. Lim, M.-W. Kim, Polyhedron 23 (2004) 903–911.

[S16] K.-Y. Choi, S.-Y. Park, Y.-M. Jeon, H. Ryu, Struct. Chem., 2005, 16, 649 - 656.

[S17] H. Arora, F. Lloret, R. Mukherjee, Inorg. Chem., Vol. 48, No. 3, 2009.

[S18] Y. Zhang, H.-C. Liang, L. N. Zakharov, S. K. Das, M. M. Hetu, A. L. Rheingold, Inorg. Chim. Acta 360 (2007) 1691–1701.

[S19] J. Shen, M. Wang, P. Zhang, J. Jiang, L. Sun. Chem. Commun., 2017, 53, 4374-4377.

[S20] K. J. Fisher, K. L. Materna, B. Q. Mercado, R. H. Crabtree, G. W. Brudvig. ACS Catal. 2017, 7, 3384−3387.

[S21] X. Zhang, Y.-Y. Li, J. Jiang, R. Zhang, R.-Z. Liao, M. Wang. Inorg. Chem. 2020, 59, 5424−5432

[S22] R. Mishra, E. Ülker, F. Karadas. ChemElectroChem 2017, 4, 75 – 80.

[S23] K. Lee, S. Vikneshvaran, H. Lee, S.-Y. Lee. Int. J. Hydrogen Energy 2024, 51, 1184-1196.

[S24] S. D. Giri and A. Sarkar. J. Electrochem. Soc. 2016, 163, H252-H259.

[S25] S. Majumder, A. A. Haleem, P. Nagarajua, Y. Naruta. Dalton Trans., 2017, 46, 9131.

[S26] A. Goswami, D. Ghosh, V. V. Chernyshev, A. Dey, D. Pradhan, K. Biradha. ACS Appl. Mater. Interfaces 2020, 12, 33679−33689.

[S27] K. Lee, S. Vikneshvaran, H. Lee, S.-Y. Lee. Int. J. Hydrogen Energy 51 (2024) 1184–1196.

[S28] H. Belhadj, Y. Messaoudi, M.R. Khelladi, A. Azizi, *Int J Hydrogen Energy.* 2022, 47, 20129–20137.

[29] J.S. Sagu, D. Mehta, K.G.U. Wijayantha, *Electrochem Commun.* 2018, 87, 1–4.

[30] Qiu, Q. F.; Chen, C. X.; Zeng, Z.; Wei, Z. W.; Zhu, N. X.; Cao, C. C.; Wang, W.; Wang, D.; Wang, H. P.; Jiang, J. J. *Inorg Chem* 2020, *59* (20), 14856–14860.
